# Molecular mechanisms of dragon’s blood in treating ulcerative colitis based on NF-κb/NLPR3/Caspase-1 pyroptosis signaling pathway

**DOI:** 10.1371/journal.pone.0331570

**Published:** 2025-09-19

**Authors:** Bilian Cai, Xiaohua Qin, Xiaogang Huang, Xiaojin He, Huanying Zhong, Yuan Yu, Jinzhu Cui, Yilei Wen

**Affiliations:** 1 Guangxi University of Chinese Medicine, Nanning, Guangxi, China; 2 First Affiliated Hospital of Guangxi University of Chinese Medicine, Nanning, Guangxi, China; Noorda College of Osteopathic Medicine, UNITED STATES OF AMERICA

## Abstract

**Objective:**

To elucidate the molecular mechanism of dragon’s blood (DB) in the treatment of ulcerative colitis (UC).

**Methods:**

Bioactive metabolites of DB absorbed into the bloodstream were characterized via LC-MS. Network pharmacology and molecular docking were employed to construct a target-pathway interaction model predicting DB’s therapeutic mechanism in UC. A 4% DSS-induced UC mouse model was used for experimental validation.

**Results:**

DB markedly alleviated colonic injury in DSS-induced UC. A total of 255 active compounds were identified, including 6,4’-Dimethoxy-7-hydroxyisoflavone, 7-hydroxy-2-(4-methoxyphenyl) chromen-4-one, and Apigenin. Key molecular targets included NLPR3, MAPK1, TP53, HIF1A, and PTGS2. The NF-κB/NLPR3/Caspase-1 axis was implicated as a central pathway mediating the therapeutic effects of DB.

**Conclusion:**

DB acts through a multi-component, multi-target, and multi-pathway strategy. Inhibiting the NF-κB/NLPR3/Caspase-1 pyroptosis pathway positions NLPR3 as a viable target for DB in UC intervention.

## 1. Introduction

Ulcerative colitis (UC) is a chronic, relapsing inflammatory condition characterized by an unpredictable clinical trajectory and unresolved etiology, involving aberrant regulation of the intestinal mucosal immune system. Clinical symptoms commonly include hematochezia, diarrhea, weight loss, and abdominal discomfort. Lesions typically originate in the rectum and may progress proximally throughout the colon, resulting in compromised mucosal barrier integrity [1,2]. Although therapeutic strategies have evolved, agents such as corticosteroids, aminosalicylates, and Janus kinase inhibitors yield inconsistent symptomatic relief and are constrained by tolerance development, adverse reactions, and partial lesion healing [3,4]. Approximately 40% of patients attain clinical remission within one year, whereas up to 30% remain refractory to pharmacologic intervention and ultimately require colectomy [5,6]. Over time, the risk of complications—including medication intolerance, disease exacerbation, chronic relapse, and malignant transformation of colonic tissue—escalates. The global incidence and prevalence of UC continue to rise, substantially impairing quality of life and exerting significant economic strain, designating the disease as a major public health issue [7]. While intestinal epithelial barrier impairment, immune dysregulation, and genetic susceptibility are implicated in its pathogenesis, the underlying mechanisms remain unclear [8].

Traditional Chinese medicine (TCM) constitutes a well-established complementary therapeutic strategy, recognized for its favorable safety profile and therapeutic efficacy. As a plant-derived remedy, TCM has received increasing scientific attention as a viable intervention for UC [9]. Dragon’s blood/RESINA DRACONIS (DB), a resin extracted from Dracaena species (Asparagaceae), exhibits significant anti-inflammatory, anti-ulcer, antibacterial, hemostatic, and analgesic activities, largely attributable to its rich composition of flavonoids, steroids, and phenolic compounds, at the same time, its efficacy in inflammatory disorders positions it as a strong candidate for UC management [10,11]. Initially identified in Yunnan Province, China, in 1972, this red resin remains a valued component of TCM formulations [12].

Pyroptosis, a pro-inflammatory mode of programmed cell death, significantly contributes to the initiation and progression of UC by mediating immune defense against microbial invasion. When dysregulated, it induces extensive host cell damage and initiates pathological inflammatory cascades. This process proceeds via two principal pathways: Caspase-1-dependent and Caspase-4/5/11-mediated mechanisms. In the former, danger signals engage specific pattern recognition receptors, triggering inflammasome assembly and subsequent Caspase-1 activation. Caspase-1 then promotes the cleavage and activation of GsdmD, a pore-forming effector that disrupts cellular membranes and facilitates the extracellular release of pro-inflammatory mediators [13]. Conversely, Caspase-4/5/11 directly cleave GsdmD, resulting in membrane rupture, cellular swelling, and cytokine discharge [14]. Across both pathways, Caspases regulate GsdmD activation, culminating in its cleavage into N- and C-terminal domains. The GsdmD-N fragment inserts into the plasma membrane, forming pores that enable the release of IL-1β and IL-18, disturb ion and water homeostasis, and precipitate osmotic lysis and cell death [15]. A growing body of evidence [16] indicates a strong correlation between UC pathogenesis and pyroptotic cell death, with inflammatory necrosis recognized as a pathological hallmark. Among the canonical pyroptotic pathways, the NF-κB/NLRP3/Caspase-1 axis is particularly prominent. Nevertheless, the specific molecular mechanisms through which DB exerts therapeutic effects on UC via this pyroptotic pathway remain undefined.

The commonly used UC model is induced via dextran sulfate sodium (DSS). Studies have demonstrated that administering 2% to 5% DSS over a 4–7 day period effectively establishes UC in animal models, with 4% DSS producing the most consistent outcomes regarding model stability and reproducibility [17,18].

Activation of the pyroptosis pore-forming effector protein GsdmD depends on proteolytic cleavage mediated by caspase family members. The pan-caspase inhibitor Z-VAD-FMK (carbobenzoxy-valylalanyl-aspartyl-[O-methyl]-fluoromethylketone, N-benzyloxycarbonyl-val-ala-asp-fluoroacetone) has been shown to efficiently suppress pyroptosis initiation [19].

TCM includes a broad spectrum of chemical constituents, biological pathways, and molecular targets. Its composite pharmacological agents exert coordinated regulatory effects on biomolecular networks, demonstrating significant efficacy in treating multifactorial diseases such as UC [20]. However, the complexity and insufficient characterization of TCM formulations present major barriers to isolating key active constituents and pinpointing precise therapeutic targets, thereby limiting its clinical research potential. Network pharmacology addresses this challenge by integrating molecular interaction networks of drugs and biological systems. By constructing component-target-pathway frameworks, it enables the systematic investigation of multi-target interactions, supports the prediction of therapeutic targets, and has proven effective in identifying bioactive constituents and clarifying the mechanistic basis of TCM interventions [21,22].

In this study, 4% DSS was employed to induce colitis in animal models, while Z-VAD-FMK was utilized to inhibit pyroptosis via the NF-κB/NLRP3/Caspase-1 axis. A DB solution at 0.11 g/ml concentration was prepared. The chemical and bioactive components of DB were profiled using LC-MS analysis. Subsequently, an integrative approach combining network pharmacology and molecular docking was used to construct a multiscale “bioactive component–target–disease” network, with the objective of delineating the molecular mechanisms through which DB modulates UC-related pathophysiology.

## 2. Materials and methods

### 2.1. Materials

#### 2.1.1. Experimental animals.

Thirty-eight male SPF BALB/c mice (6–8 weeks old, 24 ± 2 g) were sourced from Changsha Tianqin Biotechnology Co., Ltd. (approval no. SCXK (Xiang) 2022−0011). Mice were housed under controlled conditions: temperature (23 ± 2) °C, relative humidity (50 ± 5) %, and a natural light/dark cycle, with ad libitum access to standard chow and water. This study did not involve human participants. All animal-related procedures complied with the 2006 Guidelines for the Humane Treatment of Laboratory Animals issued by the Ministry of Science and Technology, People’s Republic of China. This study was approved by the Ethics Committee of Guangxi University of Traditional Chinese Medicine, adhering to principles of animal protection, welfare, and ethics, as well as relevant national regulations on the welfare and ethics of laboratory animals. Approval number: DW20240507−128. All surgeries were performed under sodium pentobarbital anesthesia, and every effort was made to minimize pain. Cervical dislocation was selected as the method of euthanasia. Inadequate anesthetic depth during procedures may exacerbate animal distress. Prior to experimentation, thorough familiarity with murine intraperitoneal injection techniques and procedural steps is essential to enhance anesthetic reliability. Anesthesia was induced via intraperitoneal administration of 3% pentobarbital sodium at 45 mg/kg. To maintain anesthetic depth and reduce nociception, additional doses of pentobarbital (10 mg/kg·h) were delivered intermittently.

The experimental model utilized the intestinal segment spanning from the anus to the ileocecal junction.

#### 2.1.2. Drugs and reagents.

DB (Xishuangbanna Banna Pharmaceutical Co., Ltd., NMPA approval number: Z20063472, batch number: 201113, specification 250 g/bottle), methanol (CNW Technologies, batch number: B1710288), acetonitrile (CNW Technologies, batch number: D925227), hydrochloric acid (Titan, batch number: P2366690), sodium chloride (Sangon Biotech, batch number: F416BA0006), acetic acid (SIGMA-ALDRICH, batch number: MKCK3410), isopropanol (CNW Technologies, batch number: F24O5K113), Z-VAD-FMK (Selleck, batch number: S7023); DSS (Yuanye Biotech, batch number: N06HS200133); mouse IL-1β ELISA kit (Fankew, batch number: F2040-A), IL-18 ELISA kit (Fankew, batch number: F2169-A), TNF-α ELISA kit (Fankew, batch number: F2132-A); p-NF-κB (p65) antibody (BIOSS, batch number: BS0982R), NLRP3 antibody (ABCAM, batch number: AB263899), Caspase-1 antibody (BOSTER, batch number: BA2220), IL-1β antibody (BOSTER, batch number: M00101-3), IL-18 antibody (BOSTER, batch number: A00124-1), TNF-α antibody (BOSTER, batch number: A00002-5), β-actin antibody (Affinity, batch number: AF7018), goat anti-rabbit secondary antibody (ZSGB-BIO, batch number: ZB2301), goat anti-mouse secondary antibody (ZSGB-BIO, batch number: ZB2305); RNA reverse transcription kit (Cwbio, batch number: CW2582M), fluorescent quantitative PCR kit (Monad, batch number: MQ00401S); occult blood kit (Beisuo, batch number: BA2020B); electron microscopy fixative (Solarbio, batch number: P1126).

#### 2.1.3. Instruments.

This study employed a comprehensive suite of instruments from various manufacturers: the Vanquish UHPLC and Orbitrap Exploris 120 high-resolution mass spectrometer (Thermo Fisher Scientific); BSA124S-CW analytical balance (Sartorius); PS-60AL ultrasonic cleaner (Shenzhen Leidebang Electronics Co., Ltd.); JXFSTPRP-24 homogenizer (Shanghai Jingxin Technology Co., Ltd.); LGJ-10C freeze dryer (Foring Technology Development Co., Ltd.); DYCZ-24DN protein electrophoresis apparatus (Beijing Liuyi Company); WSE-4040 semi-dry transfer system (ATTO); DS-11 nucleic acid/protein quantification system (Denovix, USA); 5417R refrigerated high-speed centrifuge (Eppendorf); KZ-II tissue grinder (Servicebio); CFX ConnectTM real-time PCR system (BIO-RAD); QL-902 vortex mixer (Haimen Kylin-Bell Lab Instruments Co., Ltd.); 1510 microplate reader (Thermo Fisher); Q323CW electronic balance (D&T); BX53 microscope (OLYMPUS); HT7800/HT7700 transmission electron microscope (HITACHI); and MDF-U72V ultra-low temperature freezer (SANYO).

### 2.2. Analysis of DB’s blood-entering components

#### 2.2.1. Preparation of DB suspension.

DB powder was homogenized using a blender, passed through a 200-mesh sieve, and 11 g of the filtered powder was weighed. The powder was dissolved in 100 mL of physiological saline to yield a DB suspension at a concentration of 0.11 g/mL.

#### 2.2.2. Preparation of DB drug-containing serum.

After a 7-day acclimation period, 6 BALB/c mice were randomly allocated into three groups: a control group (Group K, n = 3), a DB gavage group (Group H, n = 3), and a DB-treated group (Group YY) for LC-MS analysis. Group H received DB suspension at a dose of 5 g·kg ⁻ ¹ via oral gavage every 12 hours over 7 days. Following a 12-hour fast prior to the final administration, blood was collected via retro-orbital bleeding, centrifuged (4000 rpm, 10 min), and the serum was stored at −80°C for subsequent analysis.

#### 2.2.3. Pre-treatment of DB gavage group samples.

A 200 μL aliquot of serum was transferred to a 2 mL EP tube, followed by the addition of 20 μL of 2 mol/L HCl. The mixture was vortexed for 30 s and sonicated in an ice-water bath for 5 min. Subsequently, 780 μL of acetonitrile was added, vortexed for 30 s, and subjected to an additional 5 min of sonication under identical conditions. The sample was then incubated at −40°C for 30 min and centrifuged (4°C, 12,000 rpm, 13,800 × g, radius 8.6 cm) for 15 min. The supernatant (800 μL) was transferred to a fresh EP tube and evaporated to dryness under a nitrogen stream. The residue was reconstituted in 80 μL of extraction solvent (methanol:acetonitrile:water, 2:2:1, v/v/v) containing an isotope-labeled internal standard. After vortexing for 30 s and sonicating in an ice-water bath for 1 min, the solution was centrifuged again under the same parameters. The resulting supernatant was carefully transferred to a sampling vial for further analysis.

#### 2.2.4. Pre-treatment of DB drug group samples.

A total of 50 mg DB powder was accurately weighed and transferred into a 2 mL EP tube. Two homogenization beads and 500 μL of extraction solvent (methanol: acetonitrile: water, 2:2:1, v/v/v) containing an isotope-labeled internal standard were added. The mixture was vortexed for 30 s and homogenized at 35 Hz for 240 s. Subsequently, ultrasonication was performed in an ice-water bath for 5 min. The homogenization and ultrasonication cycle was repeated three times to ensure thorough disruption. Samples were maintained at −40°C for 30 min, then centrifuged at 4°C and 12000 rpm (13800 × g, 8.6 cm radius) for 15 min to obtain the supernatant. The supernatant was incubated at −40°C for an additional 10 min, followed by a second centrifugation under identical conditions. The final supernatant was filtered through a 0.22 μm microporous membrane and transferred to a sampling vial for subsequent analysis.

#### 2.2.5. Liquid phase detection.

Analysis was performed on a Vanquish UHPLC system equipped with a Phenomenex Kinetex C18 column (2.1 mm × 100 mm, 2.6 μm). The mobile phase consisted of phase A (0.01% acetic acid aqueous solution) and phase B (isopropanol: acetonitrile, 1:1, v/v). The sample tray was maintained at 4°C, with an injection volume of 2 μL. Data acquisition was carried out using an Orbitrap Exploris 120 mass spectrometer, operated via Xcalibur software (version 4.4, Thermo). Instrumental settings were as follows: sheath gas flow rate, 50 Arb; auxiliary gas flow rate, 15 Arb; capillary temperature, 320°C; full MS resolution, 60,000; MS/MS resolution, 15,000; stepped normalized collision energies (SNCE) of 20/30/40; and spray voltage of 3.8 kV (positive mode) or −3.4 kV (negative mode).

### 2.3. Network pharmacology

#### 2.3.1. Databases used in network pharmacology.

1. TCM Systems Pharmacology Database and Analysis Platform (TCMSP): (http://5th.tcmspw.com/tcmsp.php)
2. ChEMBL Database: (https://www.ebi.ac.uk/chembl/)
3. Traditional Chinese Medicine Integrated Database (TCMID): (http://119.3.41.228:8000/tcmid/search/)
4. PubChem: (https://pubchem.ncbi.nlm.nih.gov/)
5. UniProt Database: (https://www.uniprot.org/)
6. STRING Database: (https://string-db.org/)
7. Therapeutic Target Database (TTD): (http://db.idrblab.net/ttd/)
8. Mouse Genome Database (MGD): (http://www.informatics.jax.org)
9. GeneCards Database: (https://www.genecards.org/)
10. DAVID Database: (https://david.ncifcrf.gov/)
11. Gene Ontology (GO) Functional Enrichment: (https://www.geneontology.org)
12. KEGG Enrichment: (https://www.kegg.jp/)

#### 2.3.2. Screening of metabolite blood-entering components and target prediction.

All data were processed using the OSI/SMMS rapid identification platform for automated compound recognition. Candidate compounds were integrated from databases such as TCMSP, ChEMBL, and Traditional Chinese Medicine on Immuno-Oncology (TCMIO), applying the following thresholds: OB ≥ 30%, DL ≥ 0.18, Caco-2 permeability ≥ −0.4, and half-life (HL) ≥ 4. Target prediction for the screened blood-entering metabolites was conducted using component-associated genes from PubChem. Non-human and duplicate entries were removed, refining the list of relevant targets linked to bioavailable metabolites.

#### 2.3.3. Construction of PPI network on the DB metabolite- bioactive component-target.

Predicted targets and corresponding compound names obtained from Section 2.3.1 were collated and filtered for redundancy. Target names were normalized and verified via the UniProt database. A comprehensive metabolite–bioactive component–target interaction network for DB was established using Cytoscape 3.6.1, providing a structural framework to explore molecular mechanisms mediated by the primary metabolites.

#### 2.3.4 Construction of PPI.

Using the STRING 10.5 database with “Homo sapiens” as the designated species and “Ulcerative colitis” as the search term, targets of DB-derived blood-entering metabolites (from Section 2.3.2) were mapped under a medium confidence score threshold (0.4). The resulting interaction data were imported into Cytoscape 3.6.1, where topological analysis based on node degree facilitated the identification of core targets.

#### 2.3.5. Screening of targets related to serum drug chemical components.

Targets corresponding to serum-derived chemical constituents were retrieved from TTD, BEFREE, MGD, and GeneCards databases. Subsequent Venn diagram analysis was employed to illustrate the extent of target convergence across data sources, with each ellipse color-coded to represent a distinct dataset. This diagram quantitatively depicted both shared and unique targets across the datasets.

#### 2.3.6. Functional enrichment analysis of DB bioactive component metabolites.

Functional annotation of blood-circulating DB metabolites targeting UC was performed using GO and KEGG enrichment analyses via the DAVID database. Visualizations were generated on the Wei Sheng Xin platform. GO results were presented as bar graphs, with target proteins prioritized by degree value to identify the top 10 core genes in each GO category. Higher degree values reflected greater functional prominence in UC-associated mechanisms. KEGG analysis outcomes were similarly displayed, with pathway rankings determined by P-value magnitude—the lower the P-value, the stronger the enrichment. The top 10 pathways in each KEGG classification were emphasized. Shared targets across datasets were subjected to further integrative analysis using the R package [23].

#### 2.3.7. Construction of the interaction network of DB metabolites: “bioactive components-target-pathway” and core target screening.

Target genes corresponding to the bioactive metabolites of DB were extracted via the TCMSP database and cross-referenced with UC-related genes to identify overlapping targets. These were imported into Cytoscape 3.6.1 to generate a “bioactive components–target–pathway” network. Core targets were prioritized using degree centrality metrics via the Network Analyzer plugin.

#### 2.3.8. Molecular docking.

Molecular docking analysis was conducted to evaluate the interaction between DB-derived bioactive metabolites and the identified core target proteins. Receptor protein crystal structures were retrieved from the RCSB PDB database and prepared via structural refinement in PyMOL. Corresponding 2D chemical structures of the metabolites were sourced from PubChem and converted into compatible formats using Open Babel. Ligand conformational sampling within the active binding sites was executed using AutoDock. Binding affinities and docking interactions were evaluated through Vina software, with lower affinity scores reflecting enhanced binding stability. The most energetically favorable conformation was selected for structural visualization using PyMOL.

Regarding databases: RCSB Protein Data Bank (PDB): (https://www.rcsb.org/)

### 2.4. Animal model verification

#### 2.4.1. Drug preparation.

A 4% DSS solution was prepared by dissolving 40 g of DSS in distilled water and adjusting the final volume to 1000 mL. DB powder was pulverized using a blender and passed through a 200-mesh sieve. Subsequently, 11 g of the sieved powder was dissolved in 100 mL of normal saline to yield a 0.11 g/mL DB suspension. For Z-VAD-FMK, 45 mg was dissolved in 9.63 mL of DMSO to obtain a 1 mM solution, which was stored at −20 °C until further use. Based on preliminary studies, the DB was administered via oral gavage at 0.2 mL/10 g body weight, while Z-VAD-FMK was administered intraperitoneally at 15 mg/kg [24,25].

#### 2.4.2. Experimental design.

After 7 days of acclimatization, 32 mice were randomly divided into four groups (control, model, DB, and Z-VAD-FMK; n = 8 per group) and treated over a 6-day period. The control group received water ad libitum, while the remaining groups were administered 4% DSS to induce UC. Mice in the DB group were gavaged daily with the prepared DB suspension, whereas those in the model and Z-VAD-FMK groups received distilled water. The Z-VAD-FMK group was additionally subjected to daily intraperitoneal injections of the compound at the specified dose, while the other groups were injected with normal saline. Body weight, stool consistency, and fecal occult blood were assessed daily to calculate DAI scores.

#### 2.4.3. Specimen processing.

After the final administration, mice underwent a 12-hour fasting period without water. Blood was collected via retro-orbital sampling into EP tubes and allowed to stand for 30 minutes before centrifugation at 4°C, 3000 rpm for 15 minutes. The resulting supernatant was harvested and stored at −80°C. Euthanasia was performed by cervical dislocation, followed by laparotomy. The intestinal tract from the ileocecal junction to the anus was excised, measured, and photographed for archival purposes. A segment of the colon was fixed in 4% paraformaldehyde, and a smaller portion was preserved in 2.5% electron microscopy fixative. Remaining colon tissues were snap-frozen and stored at −80°C for subsequent analyses.

#### 2.4.4. DAI score.

Body weight, stool form, and fecal occult blood were assessed and recorded daily. The Disease Activity Index (DAI) [26] was calculated as the mean of the individual scores for weight loss, stool characteristics, and occult blood: DAI = (weight loss score + stool score + occult blood score)/3. Higher values reflected increased severity of colonic inflammation. Detailed scoring criteria are outlined in Table 1.

**Table 1:** Disease activity index (DAI) score.

Score	Weight loss rate/%	Stool characteristics	Stool occult blood
0	0	Normal	Occult blood test (-)
1	1-5	Slightly soft	Occult blood test (+)
2	6-10	Soft	Occult blood test (++)
3	11-15	Loose	Occult blood test (+++)
4	>15	Watery	Gross bloody stool

#### 2.4.5. Colon length measurement.

Following anesthesia, mice were euthanized via cervical dislocation. The intestinal tract from the ileocecal junction to the anus was excised in its entirety. Segment length was determined using a steel ruler positioned on white paper, and photographic documentation was obtained.

#### 2.4.6. Colonic histological score and alterations in mucosal goblet cells.

Colon samples were fixed in 4% paraformaldehyde for 24 h, followed by standard dehydration, paraffin embedding, microtomy, hematoxylin–eosin staining, and coverslipping to generate 3 μm-thick sections. Inflammatory cell infiltration, goblet cell depletion, and lesion penetration were evaluated microscopically based on reference criteria [27], and histological scores were assigned accordingly. Detailed scoring parameters are summarized in Table 2.

**Table 2:** Colon histological scoring criteria.

Score	Degree of inflammatory cell infiltration	Goblet cell damage	Lesion depth
0	No inflammatory cell infiltration	No goblet cell damage	No
1	Inflammatory cell infiltration at local mucosal layer or several sites	Local goblet cell loss was observed	Limited to mucosal layer
2	Inflammatory cell infiltration continuously distributed in the mucosal layer, extending to the submucosal layer	Loss of goblet cells at multiple sites, with normal epithelium	Mucosal layer and submucosa
3	Marked inflammatory cell infiltration in the mucosal and submucosal layers, continuously distributed in the submucosal layer	Numerous goblet cells disappear	Involving muscular layer
4	Severe inflammatory cell infiltration in all layers	Goblet cells completely damaged	All layers

Goblet cells were visualized by Periodic Acid-Schiff (PAS) staining. Paraffin-embedded sections were dewaxed, hydrated, and incubated in periodic acid for 10 min, followed by thorough rinsing and Schiff reagent application for 10 min. After additional rinsing, hematoxylin counterstaining (3 min) was performed. Sections were then dehydrated, cleared, mounted, and observed under a light microscope.

#### 2.4.7. ELISA of serum inflammatory factor expression.

Serum levels of IL-1β, IL-18, and TNF-α in mice were quantified according to the manufacturer’s ELISA protocol.

#### 2.4.8. Western blot analysis of p-NF-κB, NLRP3, Caspase-1, IL-1β, IL-18, and TNF-α protein expression in colon tissue.

Colon tissues were homogenized in 1000 μL lysis buffer supplemented with 10 μL PMSF per 100 mg tissue using an electric homogenizer for 30 s on ice. Lysates were centrifuged at 12,000 rpm for 10 min at 4°C, and supernatants were collected for protein analysis. Protein concentrations were determined with a nucleic acid and protein quantification system. Equal amounts (20 μg) of protein were combined with 5 × electrophoresis loading buffer, denatured at 95°C for 10 min, and cooled on ice. Samples were resolved by SDS-PAGE, transferred to PVDF membranes, and blocked for 1 h. Membranes were incubated overnight at 4°C with primary antibodies against p-NF-κB, NLRP3, Caspase-1, IL-1β, IL-18, and TNF-α (1:1000), and β-actin (1:6000) as the internal control. After three washes with 1 × TBST (10 min each), membranes were incubated in the secondary antibody solution (1:1000) at room temperature in the dark for 60 min, followed by additional TBST washes. Chemiluminescent detection was performed using ECL reagents, and membranes were developed and fixed in a darkroom. Protein bands were visualized via scanning, and signal intensities were quantified using ImageJ.

#### 2.4.9. Detection of relative mRNA expressions of NF-κB, NLRP3, Caspase-1, IL-1β, IL-18, and TNF-α in colon tissue by fluorescent quantitative PCR.

Colon tissue (100 mg) was frozen at −80°C and homogenized. Total RNA was then extracted using Trizol reagent. A 2 μL sample of RNA was used to evaluate both concentration and quality. RNA was reverse transcribed into cDNA following the manufacturer’s instructions. The reaction volume was 20 μL, with incubation at 42°C for 15 min, followed by 85°C for 5 min. β-actin was used as the internal control, and amplification was performed using a quantitative fluorescent PCR system. The PCR conditions were as follows: pre-denaturation at 95°C for 15 min, denaturation at 95°C for 10 s, annealing at 58°C for 30 s, and extension at 72°C for 30 s for 40 cycles. Relative gene expression was quantified using the 2^-△△CT^ method. Primer sequences are listed in Table 3.

**Table 3:** Primer sequences.

Primers	Sequence (5′-3′)	Length/bp
NF-κB	Upstream: GAAGCAACAGCTCACGGAGGA	128
	Downstream: TGTTCTGGAAGTTGAGGAAGGCC	
NLRP3	Upstream: ATTACCCGCCCGAGAAAGG	141
	Downstream: TCGCAGCAAAGATCCACACAG	
Caspase-1	Upstream: CCTTCCTTGTATTCATGTCTCA	152
	Downstream: GATAACCTTGGGCTTGTCTT	
IL-1β	Upstream: GCAACTGTTCCTGAACTCAACT	89
	Downstream: ATCTTTTGGGGTCCGTCAACT	
IL-18	Upstream: GACTCTTGCGTCAACTTCAAGG	169
	Downstream: CAGGCTGTCTTTTGTCAACGA	
TNF-α	Upstream: CCCTCACACTCAGATCATCTTCT	61
	Downstream: GCTACGACGTGGGCTACAG	
β-actin	Upstream: TCATCACTATTGGCAACGAGC	399
	Downstream: AACAGTCCGCCTAGAAGCAC	

#### 2.4.10. Morphological changes of pyroptosis under transmission electron microscopy.

Colon mucosal tissue (1 mm³) was fixed in electron microscopy fixative for 6 hours, followed by three 15-minute washes with 0.1 M phosphate buffer (pH 7.4). Samples were then fixed with 1% osmium tetroxide at room temperature in the dark for 2 hours and subsequently washed with phosphate buffer. Dehydration was performed using a graded ethanol and acetone series at room temperature, followed by infiltration and embedding. Ultrathin sections (60–80 nm) were prepared and stained. Morphological alterations, including damage to the cell membrane, changes in mitochondrial membrane integrity, and mitochondrial swelling, were observed under a transmission electron microscope.

### 2.5. Statistical processing

The data acquisition system consisted of a Vanquish UHPLC and Orbitrap Exploris 120 high-resolution mass spectrometer. After converting the raw data to mzXML format using ProteoWizard software, metabolite identification was conducted with the co-programmed R package. Databases used for analysis included Biotree TCM (V 1.0) and BT-HERB (V 1.0). Visual analysis was then performed using a custom R package.

### 2.6. Statistical methods

Quantitative data are presented as $\bar{x}$ ± s. Statistical analysis was performed using SPSS 21.0 software. One-way ANOVA was employed for comparisons between multiple groups, with a *P* value of < 0.05 considered statistically significant.

## 3. Results

### 3.1. Chemical component analysis of DB metabolites and screening results

**Fig 1:**
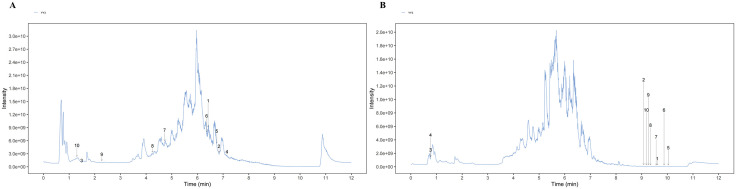
Overlay of total ions detected by mass spectrometry for QC samples, (A) Mass spectrometry YY3 positive ion, (B) Mass Spectrometry YY3 Negative Ions.

The data were imported into ProteoWizard software and converted into mzXML format. Metabolites were identified using a custom R package, with mass spectrometry data processing involving peak extraction and alignment. Visual analysis was performed with a self-developed R package, where the horizontal axis represented retention time and the vertical axis indicated the mass-to-charge ratio (m/z). Qualitative matching accuracy improved as the value approached zero. A total of 1,742 bioactive metabolites from DB were identified, and the top 10 metabolites were selected based on their TIC peak mapping scores, as shown in Fig 1. The TIC peak mapping results for DB bioactive metabolites in both positive and negative ion modes are summarized in Tables 4 and 5 (compounds listed in these tables correspond to the top 10 metabolites ranked by MS2 scores, as annotated in Fig 1: Overlay of total ions detected by mass spectrometry for QC samples).

**Table 4:** TIC peak of DB bioactive component metabolites in positive ion mode.

Number	Substance	Molecular formula	Median mass-to-charge ratio	Median retention time
1	7-hydroxy-3-phenyl-chromen-4-one	C15H10O3	239.0699	385
2	4’-Methoxyflavone	C16H12O3	253.0856	410.5
3	phenethanolamine	C8H11NO	120.0806	89.4
4	(E)-3-Hydroxy-5-methoxystilbene	C15H14O2	227.1063	429.1
5	Rubiadin-1-methyl ether	C16H12O4	269.0805	405.3
6	6,7-dimethoxy-3-(4-methoxyphenyl) chromen-4-one	C18H16O5	313.1066	381.7
7	L-allo-isoleucine	C6H13NO2	132.1015	283.4
8	Harmine	C13H12N2O	213.1019	255
9	2-Piperidone	C5H9NO	100.0754	136.3
10	Hypoxanthine	C5H4N4O	137.0455	78.2

**Table 5:** TIC peak of DB bioactive component metabolites in negative ion mode.

Number	Substance	Molecular formula	Median mass-to-charge ratio	Median retention time
1	cis-11.14-Eicosadienoic acid	C20H36O2	307.2642	576.6
2	(1S,4aR,10aR)-1,4a,7-trimethyl-7-vinyl-3,4,4b,5,6,9,10,10a-octahydro-2H-phenanthrene-1-carboxylic acid	C20H30O2	301.2172	544.6
3	Sarcosine	C3H7NO2	88.0404	46.1
4	Alanine	C3H7NO2	88.0404	46.1
5	Nonadecanoic acid	C19H38O2	297.2798	602.3
6	trans-11-Eicosenoic acid	C20H38O2	309.2798	592.4
7	trans-Vaccenic acid	C18H34O2	281.2484	574
8	cis-8,11,14-Eicosatrienoic acid	C20H34O2	305.2485	560.7
9	Linoleic acid	C18H32O2	279.2328	555.6
10	cis-9-Palmitoleic acid	C16H30O2	253.2172	550.8

### 3.2. Identification of DB chemical components and UC-related targets

**Fig 2:**
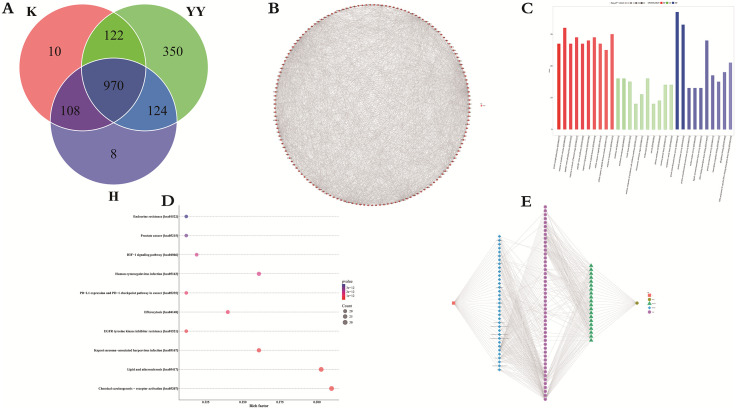
Network pharmacology analysis, (A) shared targets, (B) PPI network, (C) GO bubble diagram, (D) KEGG enrichment analysis, and (E) “component-target-pathway” network.

The targets of blood-circulating components and their therapeutic relevance to UC were analyzed using a Venn diagram (Fig 2A). Target analysis across the three groups identified 1,210 targets in the blank group, 1,566 in the DB drug group, and 1,210 in the DB gavage group. The DB gavage group shared 1,078 targets with the blank group and 1,094 with the DB drug group, with 970 targets common to all three groups. Additionally, the DB gavage group contained 8 unique targets. The shared targets predominantly impacted MAPK1, TP53, HIF1A, STAT3, and the NLRP3 inflammasome, which influenced intestinal mucosal tissues or cells, either directly or indirectly, to modulate inflammatory cytokine levels and promote mucosal repair and regeneration.

### 3.3. Construction of PPI network and identification of core targets

A total of 255 bioactive component metabolites from DB, which entered the bloodstream, were selected based on the criteria: OB ≥ 30%, DL ≥ 0.18, Caco-2 ≥ −0.4, and HL ≥ 4. These metabolites were retrieved from the ChEMBL and TCMIO databases. The top five metabolites and their respective targets are listed in Table 6. The corresponding PPI network is shown in Fig 2B. Table 7 provides detailed information about the network nodes, including the top 20 targets with the highest degree centrality. These findings suggest that the identified compounds and their core target proteins may play a central role in DB’s therapeutic effects in UC treatment.

**Table 6:** DB bioactive component metabolites and their targets.

Substance	OB	DL	Gene Name
Apigenin	73.246	0.216	AKR1B1, AR, KCNMA1, ADORA3, ESR2, ESR1, CCNB2, CDK6, OPRM1, LCK, EGFR, PTGS2, PTGS1, XDH, ABCC2, TOP2A, ABCC1, NR3C1, MAP2K1, MAPK1, MAPK14, MAPKAPK2, MAPKAPK5, PRKCA AKT1, SGK1, RPS6KB1, GSK3B, ROCK2, CHEK1, CDK2, LYN, DYRK1A, CYP2C19, HIF1A, TP53, CYP3A4, CYP2C9, ABCG2, ABCB1, HSPA5, BRCA1, PARP1, CA9
7-hydroxy-2-(4-methoxyphenyl) chromen-4-one	95.063	0.263	CYP2C19, TP53, CYP3A4, CYP2C9, HSPA5, SMPD1, BRCA1, PARP1, CA9, AR, MAPK1, HIF1A, NLRP3
6,4’-Dimethoxy-7-hydroxyisoflavone	100	0.383	TP53, SMPD1, BRCA1, NLRP3, PTGS2, AR, MAPK1
Isoformononetin	100	0.259	ESR1, TP53, CA9, PTGS2, ADORA3, PTGS1, MAPK1
Rubiadin-1-methyl ether	87.090	0.207	MAPK1, PTGS1, PTGS2, IDO1, BCL2, TP53

**Table 7:** PPI topology analysis of DB bioactive component metabolites (top 20 targets ranked by degree value).

Protein Name	Average Shortest Path Length (ASPL)	Betweenness Centrality (BC)	Closeness Centrality (CC)	Clustering Coefficient (CC)	Degree
TP53	1.36	0.09	0.74	0.29	99
AKT1	1.43	0.07	0.70	0.31	90
STAT3	1.47	0.04	0.68	0.37	81
HSP90AA1	1.50	0.05	0.67	0.36	77
SRC	1.51	0.07	0.66	0.34	77
ESR1	1.51	0.05	0.66	0.35	77
EGFR	1.52	0.03	0.66	0.38	76
BCL2	1.51	0.02	0.66	0.40	76
CTNNB1	1.53	0.03	0.66	0.39	75
NFKB1	1.53	0.03	0.66	0.40	74
PPARG	1.55	0.04	0.65	0.38	71
MAPK3	1.57	0.02	0.64	0.41	67
HIF1A	1.58	0.02	0.64	0.45	66
PTGS2	1.59	0.04	0.63	0.41	64
MTOR	1.64	0.02	0.61	0.51	57
MAPK1	1.66	0.01	0.60	0.47	55
JAK2	1.66	0.01	0.60	0.50	55
GSK3B	1.68	0.01	0.60	0.52	53
PARP1	1.69	0.01	0.59	0.50	53
CXCL8	1.70	0.02	0.58	0.51	48

### 3.4. GO and KEGG enrichment analyses

The DAVID database was utilized to analyze the bioactive components and metabolites of DB associated with UC treatment. Enrichment analysis identified key GO categories: in BP, protein autophosphorylation, xenobiotic stimulus response, and peptidyl-serine phosphorylation were predominant; in CC, enrichment was observed in raft, membrane microdomains, and vesicle lumen; and in MF, protein serine kinase activity and protein serine/threonine/tyrosine kinase activity were prominent. All *P*-values remained below 0.05 after Bonferroni correction. Table 8 and Fig 2C display the results, with GO terms on the horizontal axis and the count of differentially expressed proteins per category on the vertical. Color coding—red, green, and blue—denotes BP, CC, and MF, respectively, while color intensity reflects *P*-value magnitude, with darker shades representing stronger statistical significance. These results indicate that DB metabolites exert therapeutic effects against UC through engagement with multiple functional categories and biological processes.

**Table 8:** GO enrichment analysis of targets of DB bioactive component metabolites for UC.

GO analytical code	GO analytical classification	P	Count
GO: 0046777	protein autophosphorylation (BP)	4.24E-20	27
GO: 0009410	response to xenobiotic stimulus (BP)	2.53E-20	32
GO: 0018105	peptidyl-serine phosphorylation (BP)	1.70E-20	27
GO: 0002237	response to molecule of bacterial origin (BP)	4.00E-20	29
GO: 0018209	peptidyl-serine modification (BP)	5.25E-20	27
GO: 0032496	response to lipopolysaccharide (BP)	1.01E-19	28
GO: 0043434	response to peptide hormone (BP)	2.64E-18	29
GO: 1901653	cellular response to peptide (BP)	7.84E-18	27
GO: 0071375	cellular response to peptide hormone stimulus (BP)	1.05E-17	25
GO: 0031667	response to nutrient levels (BP)	1.25E-17	30
GO: 0045121	membrane raft (CC)	1.16E-9	16
GO: 0098857	membrane microdomain (CC)	1.22E-9	16
GO: 0031983	vesicle lumen (CC)	5.36E-8	15
GO: 0031234	extrinsic component of cytoplasmic side of plasma membrane (CC)	9.25E-8	8
GO: 0000781	chromosome, telomeric region (CC)	1.24E-7	11
GO: 0016607	nuclear speck (CC)	2.28E-7	16
GO: 0005901	caveola (CC)	2.62E-7	8
GO: 0044853	plasma membrane raft (CC)	2.74E-7	9
GO: 0034774	secretory granule lumen (CC)	3.03E-7	14
GO: 0060205	cytoplasmic vesicle lumen (CC)	3.39E-7	14
GO: 0004674	protein serine/threonine kinase activity (MF)	7.73E-27	37
GO: 0106310	protein serine kinase activity (MF)	1.12E-24	33
GO: 0004712	protein serine/threonine/tyrosine kinase activity (MF)	4.51E-17	13
GO: 0004879	nuclear receptor activity (MF)	3.72E-16	13
GO: 0098531	ligand-activated transcription factor activity (MF)	3.72 E-16	13
GO: 0140297	DNA-binding transcription factor binding (MF)	6.43 E-16	28
GO: 0004713	protein tyrosine kinase activity (MF)	2.83 E-15	17
GO: 0001221	transcription coregulator binding (MF)	3.70 E-14	15
GO: 0019902	phosphatase binding (MF)	4.03 E-14	18
GO: 0061629	RNA polymerase II-specific DNA-binding transcription factor binding (MF)	1.78 E-12	21

KEGG pathway analysis revealed 10 significantly enriched pathways (*P* *<* 0.05), including Chemical carcinogenesis – receptor activation, Lipid and atherosclerosis, PD-L1 expression and PD-1 checkpoint pathway in cancer, EGFR tyrosine kinase inhibitor resistance, and HIF-1 signaling. Fig 2D presents the corresponding bubble chart, where the X-axis represents the enrichment factor, the Y-axis shows the pathway name, bubble size corresponds to the number of involved genes, and color encodes *P*-value significance. Literature evidence indicates that these pathways directly or indirectly regulate inflammatory mediators such as MAPK1, AKT1, BCL2, PTGS2, ESR2, MAPK14, EGFR, TP53, NFKB1, and STAT3. A comprehensive summary of gene targets and their associated pathways is provided in Table 9. These data collectively imply that DB-derived metabolites may contribute to UC therapy by modulating inflammation through these enriched signaling networks.

**Table 9:** KEGG enrichment pathways related to UC.

ID	Description	Gene name	Count	P
hsa05207	Chemical carcinogenesis – receptor activation	MAPK1/BCL2/CYP3A4/AR/ESR2/ESR1/EGFR/MAP2K1/PRKCA/AKT1/RPS6KB1/PPARA/CYP1A2/CYP1A1/NFKB1/RXRA/VDR/UGT1A1/UGT1A8/AHR/MAP2K2/MAPK3/PRKACA/RPS6KA3/EPHX2/JAK2/STAT3/SRC/MTOR/HSP90AA1	30	3.52E-20
hsa05417	Lipid and atherosclerosis	MAPK1/BCL2/TP53/CYP2C9/HSPA5/NLRP3/MAPK14/PRKCA/AKT1/GSK3B/ROCK2/LYN/PPARG/CYP1A1/NFKB1/NFE2L2/RXRA/CXCL8/CYBB/CAMK2A/CAMK2G/MAPK3/JAK2/STAT3/IRF3/SRC/HSP90AA1/CASP7/CASP1	29	6.30E-19
hsa05167	Kaposi sarcoma-associated herpesvirus infection	MAPK1/PTGS2/TP53/HIF1A/CDK6/MAP2K1/MAPK14/MAPKAPK2/AKT1/GSK3B/LYN/SYK/NFKB1/CXCL8/JAK1/MAP2K2/MAPK3/JAK2/STAT3/IRF3/SRC/MTOR/CTNNB1	23	6.66E-14
hsa01521	EGFR tyrosine kinase inhibitor resistance	MAPK1/BCL2/EGFR/MAP2K1/PRKCA/AKT1/RPS6KB1/GSK3B/JAK1/MAP2K2/MAPK3/PDGFRB/JAK2/STAT3/SRC/MTOR	16	1.16E-13
hsa04148	Efferocytosis	MAPK1/PTGS2/HIF1A/MAP2K1/MAPK14/MAPKAPK2/SGK1/PPARG/ALOX15/RXRA/PPARD/ALOX5/CAMK2A/CAMK2G/MAP2K2/MAPK3/PTPN6/JAK2/CASP7/CASP1	20	7.47E-13
hsa05235	PD-L1 expression and PD-1 checkpoint pathway in cancer	MAPK1/HIF1A/LCK/EGFR/MAP2K1/MAPK14/AKT1/RPS6KB1/NFKB1/JAK1/MAP2K2/MAPK3/PTPN6/JAK2/STAT3/MTOR	16	8.25E-13
hsa05163	Human cytomegalovirus infection	MAPK1/PTGS2/TP53/CDK6/EGFR/MAP2K1/MAPK14/PRKCA/AKT1/RPS6KB1/GSK3B/ROCK2/NFKB1/CXCL8/JAK1/MAP2K2/MAPK3/PRKACA/STAT3/IRF3/SRC/MTOR/CTNNB1	23	1.60E-12
hsa04066	HIF-1 signaling pathway	MAPK1/BCL2/HIF1A/EGFR/MAP2K1/PRKCA/AKT1/RPS6KB1/NFKB1/CYBB/CAMK2A/CAMK2G/MAP2K2/MAPK3/INSR/STAT3/MTOR	17	1.73E-12
hsa05215	Prostate cancer	MAPK1/BCL2/TP53/AR/EGFR/MAP2K1/AKT1/GSK3B/CDK2/NFKB1/MAP2K2/MAPK3/PDGFRB/MTOR/HSP90AA1/CTNNB1	16	3.31E-12
hsa05207	Chemical carcinogenesis – receptor activation	MAPK1/BCL2/CYP3A4/AR/ESR2/ESR1/EGFR/MAP2K1/PRKCA/AKT1/RPS6KB1/PPARA/CYP1A2/CYP1A1/NFKB1/RXRA/VDR/UGT1A1/UGT1A8/AHR/MAP2K2/MAPK3/PRKACA/RPS6KA3/EPHX2/JAK2/STAT3/SRC/MTOR/HSP90AA1	30	3.52E-20

### 3.5 Construction of DB bioactive component metabolites “bioactive components-target-pathway” interaction network and screening of core targets

The interaction network linking DB-derived bioactive metabolites, targets, and pathways associated with UC treatment was established using Cytoscape 3.6.1, as illustrated in Fig 2E. Distinct node shapes were employed to improve interpretability of the regulatory relationships. Specifically, the orange-red square indicated the original TCM formulation; blue diamonds represented 35 DB metabolites; pink circles denoted 50 protein targets; green triangles marked 20 pathways shared between the targets and UC; and the yellow-brown circle referred to the disease entity. The resulting network comprised 107 nodes and 940 edges, with an average shortest path length of 2.44, a mean node degree of 8.79, a centrality proximity of 0.41, and a network centrality of 0.01. These metrics reflect that the 35 bioactive metabolites act upon 50 protein targets through direct or indirect interactions, collectively modulating 20 signaling pathways implicated in intestinal mucosal protection and ulcer repair via multifaceted mechanisms.

### 3.6. Molecular docking of DB bioactive component metabolites and core target proteins

**Fig 3:**
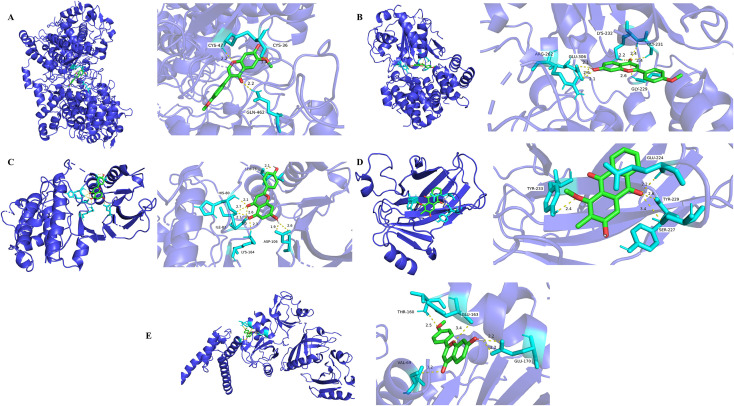
Molecular docking of DB bioactive component metabolites and core targets.

Five UC-associated targets—MAPK1, TP53, HIF1A, PTGS2, and NLRP3—were prioritized based on PPI network degree ranking and literature validation. Corresponding bioactive metabolites selected for docking included 6,4’-Dimethoxy-7-hydroxyisoflavone, 7-hydroxy-2-(4-methoxyphenyl) chromen-4-one, Apigenin, Rubiadin-1-methyl ether, and Isoformononetin. Docking outcomes are detailed in Table 10. Binding affinities below –5 kcal·mol ⁻ ¹ were interpreted as indicative of strong ligand–target interactions, with more negative values reflecting greater stability. Afrormosin (6,4’-Dimethoxy-7-hydroxyisoflavone) exhibited stable binding to PTGS2 via multiple hydrogen bonds involving cysteine and glutamine residues (Fig 3A). Pratol (7-hydroxy-2-(4-methoxyphenyl) chromen-4-one) engaged NLRP3 through hydrogen bonds with arginine, glutamic acid, lysine, and glycine (Fig 3B). Apigenin formed interactions with MAPK1 through leucine, histidine, lysine, isoleucine, and aspartic acid residues (Fig 3C). Rubiadin-1-methyl ether established hydrogen bonding with TP53 via tyrosine, glutamic acid, and serine (Fig 3D). Pratol also bound HIF1A through hydrogen bonds involving threonine, valine, and glutamic acid residues (Fig 3E).

**Table 10:** Molecular docking of DB bioactive component metabolites and core targets.

Compound\Affinity(kcal·mol^ − 1^)\Target	MAPK1	NLRP3	TP53	HIF1A	PTGS2
6,4’-Dimethoxy-7-hydroxyisoflavone (Afrormosin)	−5.29	1.19	−6.32	1.19	−7.51
Pratol(7 − hydroxy−2−(4 − methoxyphenyl) chromen−4 − one)	−6.2	−7.61	−6.34	−7.09	−7.92
Apigenin	−6.8	−5.93	−6.6	−6.52	−7.43
Rubiadin-1-methyl ether	−5.88	−6.56	−6.93	−6.56	−7.51
Isoformononetin	−6.13	−6.39	136.53	–	−7.08

### 3.7. DB had a protective effect on colon tissue in UC induced by 4%DSS

**Fig 4:**
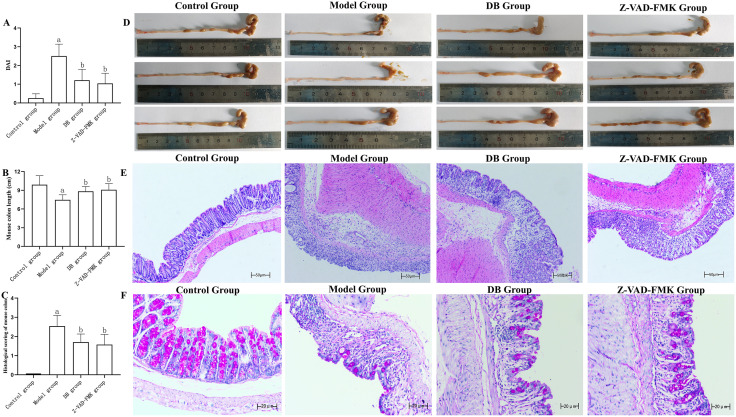
Effect of DB on UC colon tissue. **(A)** DAI score, (B) colon length, (C) colon histology score, (D) colon length measurement, (E) colon tissue, **(F)**Representative images of colonic goblet cells by Periodic Acid-Schiff (PAS) staining.

The reparative capacity of DB in 4% DSS-induced UC was evaluated using DAI scores, colon length measurements, and histopathological assessment. Post-treatment observations (Fig 4A) revealed no signs of colonic inflammation in the control group. In contrast, the model group exhibited marked weight loss, loose stools, and positive occult blood tests (*P* *< *0.01). Compared to the model group, mice treated with DB or Z-VAD-FMK demonstrated reduced stool looseness, limited weight loss (1–5%), and attenuated occult blood positivity (*P* *< *0.01). Colon length served as an indirect marker of tissue integrity. Model group mice showed significant colon shortening (*P* *< *0.01), whereas treatment with DB or Z-VAD-FMK led to substantial recovery, with colon lengths significantly exceeding those of the model group (*P* *< *0.05) (Fig 4B, D). Histological analysis (Fig 4C, E) further characterized tissue alterations. The model group presented with significantly elevated histological scores (*P* *< *0.01) relative to controls, indicating pronounced mucosal disruption. DB and Z-VAD-FMK administration markedly reduced these scores (*P* *< *0.05), reflecting structural preservation. Control mice displayed intact mucosal structure with no inflammatory infiltration or goblet cell damage. Conversely, the model group showed diffuse inflammatory cell infiltration extending from the mucosa to the submucosa, accompanied by severe goblet cell depletion. DB-treated tissues exhibited confined inflammatory infiltration limited to the mucosal layer and focal goblet cell loss, while regions of intact epithelium remained preserved. Similar findings were observed in the Z-VAD-FMK group, with patchy mucosal infiltration and region-specific goblet cell reduction(Fig 4F). Collectively, the data indicate that both DB and Z-VAD-FMK attenuate DSS-induced colonic damage and enhance mucosal repair in UC.

### 3.8. DB effectively inhibited the release of inflammatory factors and cell membrane structural damage

**Fig 5:**
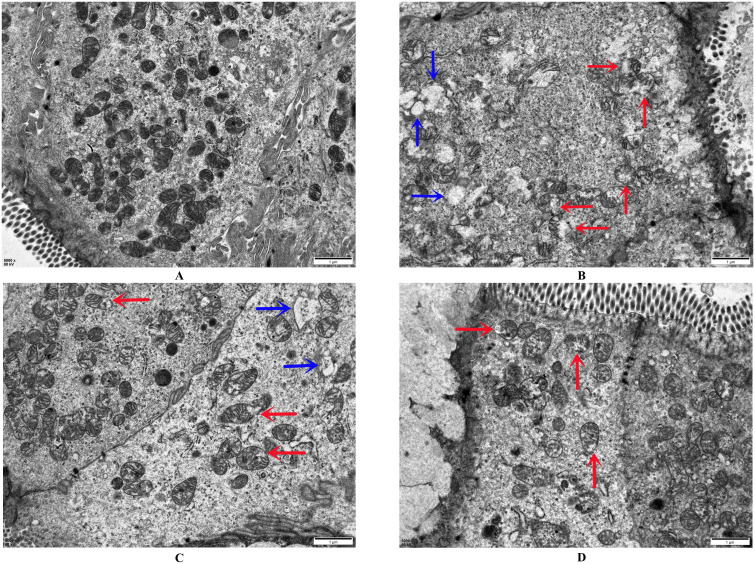
Pyroptosis of colon mucosal tissue in each group (transmission electron microscopy, × 5000), A Control group, B Model group, C DB group, D Z-VAD-FMK group.

To evaluate the inhibitory effect of DB on pyroptosis, ELISA and transmission electron microscopy were employed to quantify inflammatory cytokine levels and assess ultrastructural changes in cellular membranes. ELISA results (Table 11) demonstrated significantly increased serum levels of IL-1β, IL-18, and TNF-α in the model group compared to the control group (*P* *<* 0.01). Treatment with DB and Z-VAD-FMK markedly reduced the levels of these cytokines relative to the model group (*P* *<* 0.01). Transmission electron microscopy (Fig 5) showed intact cellular and mitochondrial membranes in the control group, without evidence of mitochondrial swelling, disruption, or blebbing. In contrast, the model group exhibited pronounced mitochondrial membrane rupture and swelling (red arrows), accompanied by extensive membrane blebbing (blue arrows). The DB group displayed partially preserved mitochondrial integrity, though some organelles exhibited swelling and rupture (red arrows), with limited blebbing (blue arrows). In the Z-VAD-FMK group, the cell membrane remained intact, while a subset of mitochondria showed swelling and rupture (red arrows), without detectable blebbing. Collectively, the data indicate that DB suppresses systemic inflammatory responses in UC mice and alleviates structural damage to cellular and mitochondrial membranes.

**Table 11:** Expression of serumIL-1β, IL-18, and TNF-α in mice ($\bar{x}$ ± s, n = 8).

Group	IL-1β (pg/ml)	IL-18 (pg/ml)	TNF-α (pg/ml)
Control Group	10.01 ± 1.72	10.77 ± 1.73	13.14 ± 2.00
Model Group	70.49 ± 4.24^*^	113.08 ± 5.42^*^	508.80 ± 21.33^*^
DB Group	53.95 ± 3.02^#^	84.34 ± 3.60^#^	343.78 ± 16.18^#^
Z-VAD-FMK Group	47.48 ± 3.67^#^	72.94 ± 2.98^#^	245.20 ± 17.54^#^

Note: Compared with the control group, *P* *< 0.01; compared with the model group, #*P* *< *0.01.

### 3.9. DB inhibited pyroptosis through the NF-κB/NLRP3/Caspase-1 signaling pathway

**Fig 6:**
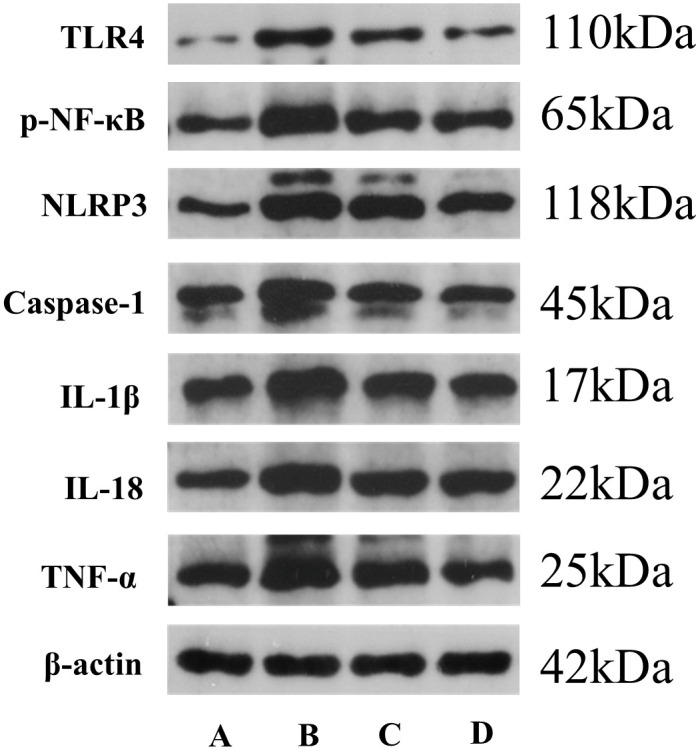
Electrophoresis of TLR4, p-NF-κB, NLRP3, Caspase-1, IL-1β, IL-18, and TNF-α protein expression in mouse colon tissue. Note: A: control group, B: model group, C: DB group, and D: Z-VAD-FMK group.

To elucidate the mechanism by which DB mitigates pyroptosis, Western blot and quantitative PCR analyses were conducted to evaluate the expression levels of p-NF-κB, NLRP3, Caspase-1, IL-1β, IL-18, and TNF-α at both the protein and mRNA levels in murine colonic tissue. Western blot results (Table 12 and Fig 6) demonstrated a marked upregulation of p-NF-κB, NLRP3, Caspase-1, IL-1β, IL-18, and TNF-α proteins in the model group compared to controls (*P* *<* 0.01). Treatment with DB and Z-VAD-FMK significantly attenuated the protein expression of p-NF-κB, NLRP3, IL-1β, IL-18, and TNF-α (*P* *<* 0.01). Caspase-1 protein levels were also reduced in the DB group (*P* *<* 0.05), with a more pronounced decrease observed in the Z-VAD-FMK group (*P* *<* 0.01). Quantitative PCR data (Table 13) revealed significant transcriptional upregulation of NF-κB, NLRP3, Caspase-1, IL-1β, IL-18, and TNF-α mRNA in the model group (*P* *<* 0.01). DB and Z-VAD-FMK treatments significantly downregulated the mRNA levels of NF-κB, Caspase-1, IL-1β, IL-18, and TNF-α relative to the model group (*P* *<* 0.01). NLRP3 mRNA expression was also suppressed in the DB group (*P* *<* 0.05), with a more substantial reduction observed following Z-VAD-FMK administration (*P* *<* 0.01). Collectively, the results indicate that DB suppresses pyroptotic activity by modulating the NF-κB/NLRP3/Caspase-1 signaling axis.

**Table 12:** Expression levels of TLR4, NF-κB, NLRP3, Caspase-1, IL-1β, IL-18, and TNF-α proteins in mouse colon tissues ($\overset{―}{x}$±s, n = 3).

Group	TLR4(pg/ml)	p-NF-κB(pg/ml)	NLRP3(pg/ml)	Caspase-1(pg/ml)	IL-1β(pg/ml)	IL-18(pg/ml)	TNF-α(pg/ml)
Control Group	0.03 ± 0.01	0.32 ± 0.13	0.26 ± 0.09	0.55 ± 0.16	0.63 ± 0.26	0.43 ± 0.07	0.58 ± 0.20
Model Group	0.89 ± 0.12^*^	1.99 ± 0.56^*^	1.82 ± 0.40^*^	2.31 ± 0.58^*^	3.10 ± 0.80^*^	2.43 ± 0.34^*^	2.54 ± 0.72^*^
DB Group	0.37 ± 0.09^#^	0.85 ± 0.52^#^	0.77 ± 0.31^#^	1.21 ± 0.54^▲^	1.31 ± 0.46^#^	1.25 ± 0.27^#^	1.24 ± 0.42^#^
Z-VAD-FMK Group	0.19 ± 0.06^#^	0.52 ± 0.23^#^	0.49 ± 0.23^#^	0.89 ± 0.69^#^	0.99 ± 0.46^#^	0.76 ± 0.23^#^	0.79 ± 0.60^#^

Note: Compared with the control group, *P* *< 0.01; compared with the model group, ▲*P* *< *0.05, #*P* *< *0.01.

**Table 13:** mRNA expression of NF-κB, NLRP3, Caspase-1, IL-1β, IL-18, and TNF-α in mouse colon tissue ($\bar{x}$ ± s, n = 5).

Group	NF-κB	NLRP3	Caspase-1	IL-1β	IL-18	TNF-α
Control Group	1.01 ± 0.13	1.24 ± 0.17	1.17 ± 0.23	1.17 ± 0.20	1.12 ± 0.18	1.10 ± 0.21
Model Group	5.78 ± 0.57^*^	7.51 ± 0.94^*^	6.00 ± 0.70^*^	7.46 ± 0.87^*^	6.98 ± 0.89^*^	5.43 ± 0.69^*^
DB Group	4.37 ± 0.96^#^	6.60 ± 0.45^▲^	4.65 ± 0.93^#^	4.88 ± 0.53^#^	5.50 ± 0.47^#^	4.14 ± 0.51^#^
Z-VAD-FMK Group	3.20 ± 0.47^#^	5.27 ± 0.84^#^	3.59 ± 0.57^#^	4.08 ± 0.69^#^	4.43 ± 0.86^#^	2.86 ± 0.30^#^

Note: Compared with the control group, *P* *< 0.01; compared with the model group, ▲*P* *< *0.05, #*P* *< *0.01.

## 4. Discussion

This study presents four principal conclusions: (1) NLPR3 functions as a therapeutic target of DB for the prevention and treatment of UC. At a concentration of 0.11 g/ml, DB appears to suppress pyroptosis by modulating the NF-κB/NLPR3/Caspase-1 signaling pathway, thereby contributing to UC mitigation. (2) Z-VAD-FMK effectively inhibits Caspase family activity and demonstrates therapeutic potential in UC models induced by 4% DSS. (3) DB operates through a multi-component, multi-target, and multi-pathway framework, potentially attenuating pyroptosis indirectly by downregulating inflammatory mediators and interfering with pyroptosis-initiating signals.

As shown in Fig 4, 4% DSS triggered intestinal injury and UC development in mice, while treatment with 0.11 g/ml DB and Z-VAD-FMK enhanced goblet cell production, promoted epithelial hyperplasia, and alleviated intestinal pathology.

UC is characterized by compromised intestinal barrier integrity, manifested as goblet cell depletion, diminished mucin secretion, and elevated bacterial translocation [28]. Under physiological conditions, a diverse microbiota colonizes the mucosal surface, maintaining symbiosis with the host. However, in pathological contexts, this microbial community may transition into a pathogenic state, contributing to intestinal dysfunction. The intestinal mucus barrier is instrumental in protecting host tissues from microbial invasion and preserving epithelial cell integrity, particularly that of goblet cells [29]. In the colon, this barrier comprises a bilayered mucus structure: an inner dense layer that physically separates bacteria from epithelial cells and an outer layer that is less compact, serving as a microbial adhesion site. Goblet cells, as specialized epithelial secretory cells, are responsible for mucin synthesis and play a vital role in mediating host–microbiota interactions and sustaining intestinal equilibrium [30].

Previous research has linked disruption of the intestinal mucosal barrier to heightened activity of colonic immune cells, immune dysregulation, and microbial imbalance—hallmarks of pyroptosis-associated injury in UC [31,32]. Pyroptosis is initiated by the recognition of PAMPs and DAMPs, which act as primary molecular cues. Intestinal immune cells expressing PRRs, including Toll-like receptors (TLRs), detect these signals. Upon epithelial damage, barrier integrity is impaired, increasing permeability to Gram-negative bacteria and bacterial components such as lipopolysaccharides. These elements subsequently activate macrophage-expressed PRRs. Specifically, engagement of TLR4 by pathogen-associated ligands triggers a pyroptotic signaling pathway and enhances the synthesis and secretion of proinflammatory mediators. Upregulated TLR4 promotes activation of MyD88 and NF-κB, leading to transcriptional upregulation of cytokines such as TNF-α, IL-1β, and IL-6, thereby intensifying mucosal inflammation and aggravating barrier dysfunction [33], Simultaneously, macrophages and neutrophils are activated and undergo Caspase-1-dependent pyroptosis, releasing proinflammatory cytokines and reactive oxygen and nitrogen species. These cytotoxic mediators inflict further tissue injury, increase mucosal permeability, and drive the translocation of luminal contents, creating a self-perpetuating cycle of inflammation and epithelial damage that complicates therapeutic intervention [34,35]. Following epithelial disruption, dysregulated immune cell activity within colonic tissue induces aberrant inflammatory responses and reinforces pyroptotic signaling via receptor-mediated activation of NF-κB. Notably, DB demonstrates the capacity to attenuate colonic damage induced by 4% DSS and supports epithelial regeneration and tissue repair.

To elucidate the molecular mechanism underlying DB-mediated intervention in UC, a multi-level approach integrating network pharmacology, molecular docking, and in vivo experimentation (refer to Tables 10 and 12–13, Figs 3 and 6) identified NLRP3 as a potential molecular target. Subsequent murine studies confirmed that DB administration markedly attenuated protein expression of NF-κB, NLRP3, and Caspase-1. These results indicate that DB alleviates UC by modulating the pyroptosis-associated signaling pathway, specifically the NF-κB/NLRP3/Caspase-1 axis, with NF-κB and NLRP3 serving as principal molecular targets

NF-κB, a nuclear transcription factor integral to pyroptosis regulation, functions upstream in the Caspase-1 signaling pathway and regulates NLRP3 inflammasome formation. It also acts as a signal amplifier by promoting the synthesis and secretion of inflammatory mediators [36].

Elevated NF-κB activity has been documented in UC patients. As a core regulator of inflammation, NF-κB modulates the release of proinflammatory cytokines, chemokines, and adhesion molecules [37,38]. Within the Caspase-1-mediated pyroptotic pathway, NF-κB initiates the signaling sequence. Upon PAMP stimulation, it governs transcriptional upregulation and secretion of downstream effectors, including IL-1β, IL-18, and NLRP3, which subsequently trigger the second signal required for pyroptosis—activation via damage-associated stimuli [39,40].

NLRP3, a cytoplasmic PRR, is composed of a pyrin domain (PYD), a nucleotide-binding and oligomerization domain (NACHT), and a leucine-rich repeat (LRR) sequence [41]. Its transcription is regulated by NF-κB, and upon sensing danger signals, it initiates inflammasome assembly. This process activates Caspase-1, leading to the maturation of inflammatory cytokines and induction of pyroptosis [42]. To investigate the molecular mechanism by which DB targets the NF-κB/NLRP3/Caspase-1 axis, Fig 3 and Table 10 demonstrate that Pratol (7-hydroxy-2-(4-methoxyphenyl) chromen-4-one), a bioactive metabolite of DB, interacts with NLRP3 primarily through multiple hydrogen bonds, potentially modulating inflammasome formation. Previous studies [43,44] have established that NLRP3 inflammasome assembly depends on NLRP3 oligomerization. Upon activation, the LRR domain binds to specific ligands, promoting NACHT domain exposure and subsequent oligomerization. Concurrently, the PYD domain associates with the PYD of apoptosis-associated speck-like protein (ASC), enabling the recruitment of ASC and Pro-Caspase-1 into a multiprotein complex defined as the NLRP3 inflammasome. Evidence suggests that small-molecule inhibitors can disrupt this oligomerization by forming hydrogen bonds with NLRP3, thereby preventing ASC binding and impairing inflammasome assembly [45]. These inhibitors may further interfere with NLRP3-associated signaling interactions, ultimately attenuating pyroptotic responses. Following inflammasome formation, Pro-Caspase-1 undergoes proteolytic cleavage into its active form [46], which subsequently activates GsdmD as well as proinflammatory cytokine precursors, including pro-IL-1β and pro-IL-18. This activation results in pore formation within the cell membrane and the subsequent release of inflammatory intracellular contents [47,48].

Network pharmacology offers a predictive framework for hypothesizing compound–target–pathway interactions by integrating computational target identification with pathway mapping; however, it lacks the capacity to confirm biological target engagement or functional activity. In this study, NLRP3 emerged as a candidate target of DB-derived active constituents, implying possible modulation of NLRP3-mediated inflammatory responses. Subsequent in mouse experiments demonstrated that DB administration significantly reduced the protein expression levels of NF-κB, NLRP3, and Caspase-1, thereby suppressing pyroptosis and alleviating UC-related pathology. In addition, related studies have also shown that NF-κB is an effective target for the treatment of UC with DB [9]. These findings substantiate DB’s capacity to inhibit the NF-κB/NLRP3/Caspase-1 axis at multiple nodes, affirming both NF-κB and NLRP3 as functionally relevant targets. The integrated rationale relies on progressive validation: literature-based mechanistic insights provide foundational support, network pharmacology delineates potential targets, and animal models validate systemic biological outcomes. Collectively, the data indicate that DB mediates its anti-UC effects via coordinated suppression of the NF-κB/NLRP3/Caspase-1 signaling pathway, with NF-κB and NLRP3 serving as upstream regulatory elements within this pathway.

As illustrated in Fig 5 and detailed in Table 11, 4% DSS exposure induced mitochondrial membrane disruption and elevated levels of inflammatory markers, including IL-1β, IL-18, and TNF-α, in colonic tissues. Previous research has demonstrated impaired antioxidant defense in the colonic mucosa of UC patients, characterized by sustained oxidative injury and aggravated inflammation that perpetuates a pathological cycle [49]. Mitochondria serve as the principal source of ROS generation, and the pyroptosis effector GSDMD-NT impairs mitochondrial integrity by disrupting both inner and outer membranes, thereby impairing mitochondrial function, diminishing regenerative capacity, and promoting excessive mitochondrial reactive oxygen species (mtROS) release [46,50]. Acting as a DAMP signal, mtROS not only exacerbates oxidative injury but also enhances inflammasome assembly [51,52]. In addition, ROS contributes to NLRP3 induction and functions as a strong activator of NF-κB, thereby accelerating pyroptotic signaling pathways [53]. DB treatment markedly reduced pro-inflammatory mediator expression, consistent with previous reports on its regulatory effects [54]. Ultrastructural analysis via transmission electron microscopy revealed a decline in membrane blebbing in colonic epithelial cells of DB-treated mice, alongside restoration of mitochondrial morphology.

This study also demonstrated that Z-VAD-FMK suppressed Caspase-1 expression and concurrently downregulated upstream pyroptosis-related molecules, including NF-κB and NLRP3, thereby enhancing colonic tissue regeneration and epithelial repair. This outcome aligns with earlier studies [55]. IL-1β, TNF, and ROS have been reported to activate NF-κB through interrelated pathways, with a documented positive correlation among them [56–58]. Inhibition of Caspase-1 not only disrupts the pyroptotic cascade but also prevents pro-inflammatory cytokine release, limits oxidative damage, and reduces ROS production. The intrinsic regenerative capacity of colonic tissue likely contributes to the downregulation of additional inflammatory mediators in the Z-VAD-FMK group beyond Caspase-1. Comparative analysis between Z-VAD-FMK and DB treatments indicated that Z-VAD-FMK exerted a more significant regulatory effect, likely attributable to its targeted inhibition of caspase activity. GsdmD activation depends on cleavage by the Caspase family; Z-VAD-FMK blocks both Caspase-1 and Caspase-4/5/11, thereby suppressing both classical and non-classical pyroptosis pathways. Nevertheless, due to its broad-spectrum inhibitory profile, Z-VAD-FMK lacks therapeutic suitability for UC [15,59].

This study further indicated that DB exerted effects comparable to Z-VAD-FMK. Considering its favorable safety profile, cost-effectiveness, and low toxicity, DB represents a promising candidate for UC treatment.

This study utilized the caspase-selective inhibitor Z-VAD-FMK to delineate differential suppression profiles of key nodal proteins within the NF-κB/NLRP3/Caspase-1 pyroptosis axis, revealing a bidirectional regulatory mechanism. Although Z-VAD-FMK directly inhibits Caspase-1, it concurrently reduces upstream NF-κB and NLRP3 activity, suggesting a negative feedback loop that propagates inhibitory signals from downstream effectors toward upstream regulators, thereby mitigating pyroptosis and alleviating UC-related pathology. In contrast, DB attenuates Caspase-1 expression indirectly by targeting NF-κB and NLRP3 with higher binding affinity. Experimental data indicate that DB preferentially interacts with NF-κB and NLRP3 rather than Caspase-1, a finding supported by network pharmacology analysis identifying NLRP3 as the central target, consistent with existing evidence implicating NF-κB in UC pathogenesis. These observations imply that DB exerts its regulatory effect through top-down modulation of the pathway, initiating inhibition at proximal nodes and transmitting suppression downstream. Although both agents converge on the NF-κB/NLRP3/Caspase-1 axis to suppress pyroptotic signaling, their regulatory trajectories differ fundamentally: Z-VAD-FMK elicits a feedback-mediated, bottom-up inhibition, whereas DB enforces suppression through a feedforward, upstream-directed mechanism

## 5. Conclusion

DB constitutes a promising therapeutic option for UC, exerting its efficacy through a multi-component, multi-target, and multi-pathway mode of action. Its pharmacological profile demonstrates both precision and safety. The therapeutic mechanism primarily involves the suppression of pyroptosis via downregulation of the NF-κB/NLRP3/Caspase-1 signaling pathway, thereby contributing to the prevention and treatment of UC. As a representative form of intestinal inflammation, UC is characterized by dysregulated mucosal immunity and frequent compromise of the intestinal epithelial barrier. DB enhances colonic tissue regeneration and suppresses pyroptotic activity, supporting barrier restoration and mitigating pyroptosis-associated injury. In addition, the bioactive constituents of DB directly interact with NLRP3, attenuating inflammasome activation and downregulating NF-κB signaling to disrupt pyroptotic progression. DB also reduces DAMPs recruitment by limiting the release of proinflammatory mediators and promoting mitochondrial membrane stabilization, further contributing to the inhibition of pyroptosis.

### Limitations

The present study exclusively examines the NF-κB/NLPR3/Caspase-1 signaling pathway in the context of UC. Subsequent research will incorporate additional molecular markers to clarify the broader mechanistic basis of DB’s preventive and therapeutic actions against UC.

### Highlights

1. DB mitigates UC primarily by suppressing pyroptosis via the NF-κB/NLPR3/Caspase-1 axis.
2. NF-κb and NLPR3 serve as the key targets of DB.
3. Suppression of Caspase family expression effectively inhibits pyroptosis.

## Supporting information

S1 File Bar Chart. (ZIP)

S2 File Colon image. (ZIP)

S3 File Network pharmacology original diagram. (ZIP)

S4 File Original image selected by PAS. (ZIP)

S5 File PAS selected marked scale image. (ZIP)

S6 File S1_raw_images. (PDF)

S7 File Ulcerative colitis HE image 40x magnification. (ZIP)

## Abbreviations

DB: Dragon’s blood
UC: Ulcerative colitis
SS: Dextran sulfate sodium
