# Supplementary figures and images for: Molecular mechanisms of dragon’s blood in treating ulcerative colitis based on NF-κb/NLPR3/Caspase-1 pyroptosis signaling pathway
Source: PLoS One. 2025 Sep 19;20(9):e0331570. doi: 10.1371/journal.pone.0331570 (PMC12448351; doi:10.1371/journal.pone.0331570)

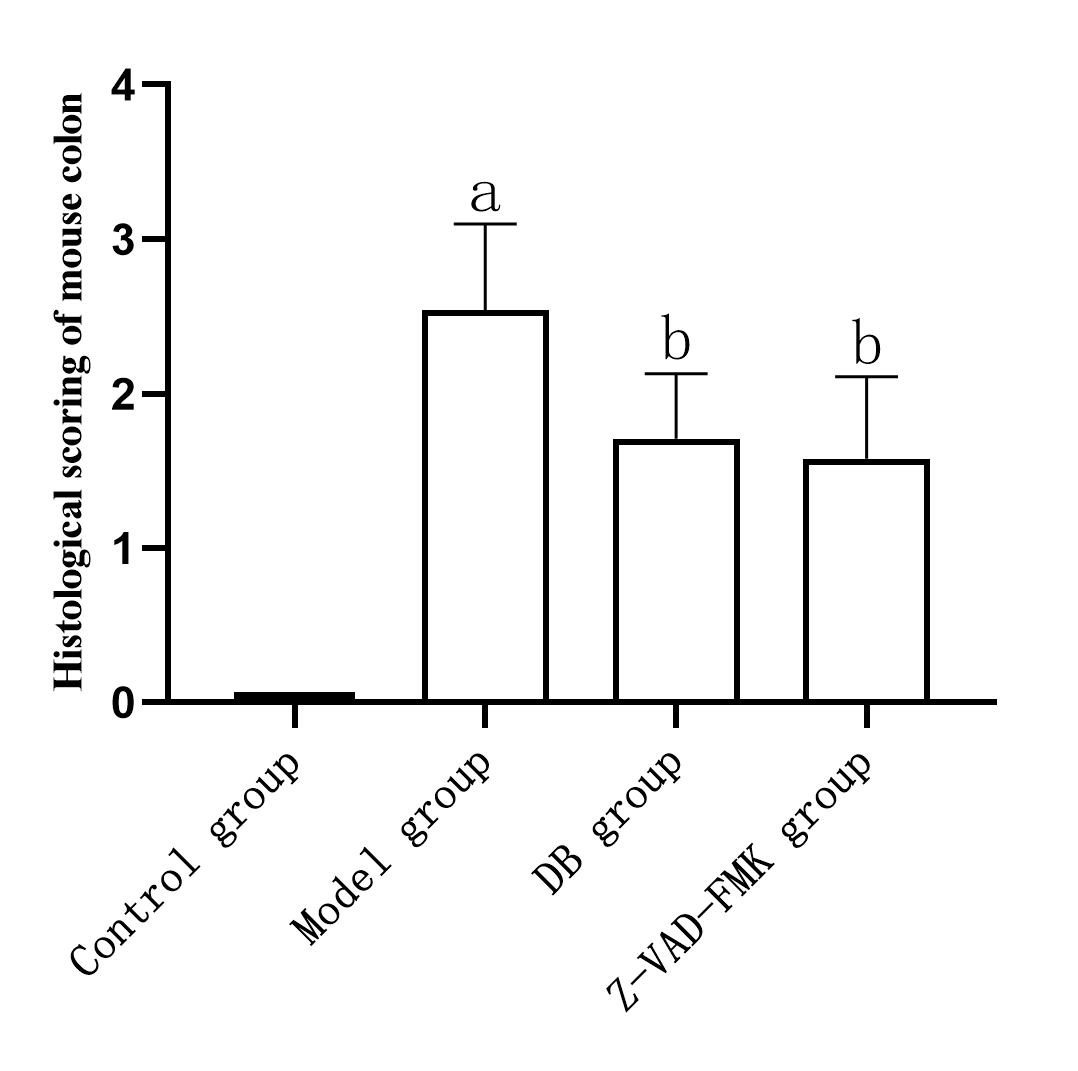

Supplement: S1 File — (ZIP) [file pone.0331570.s001.zip › Bar Chart/colon histology score.bmp]

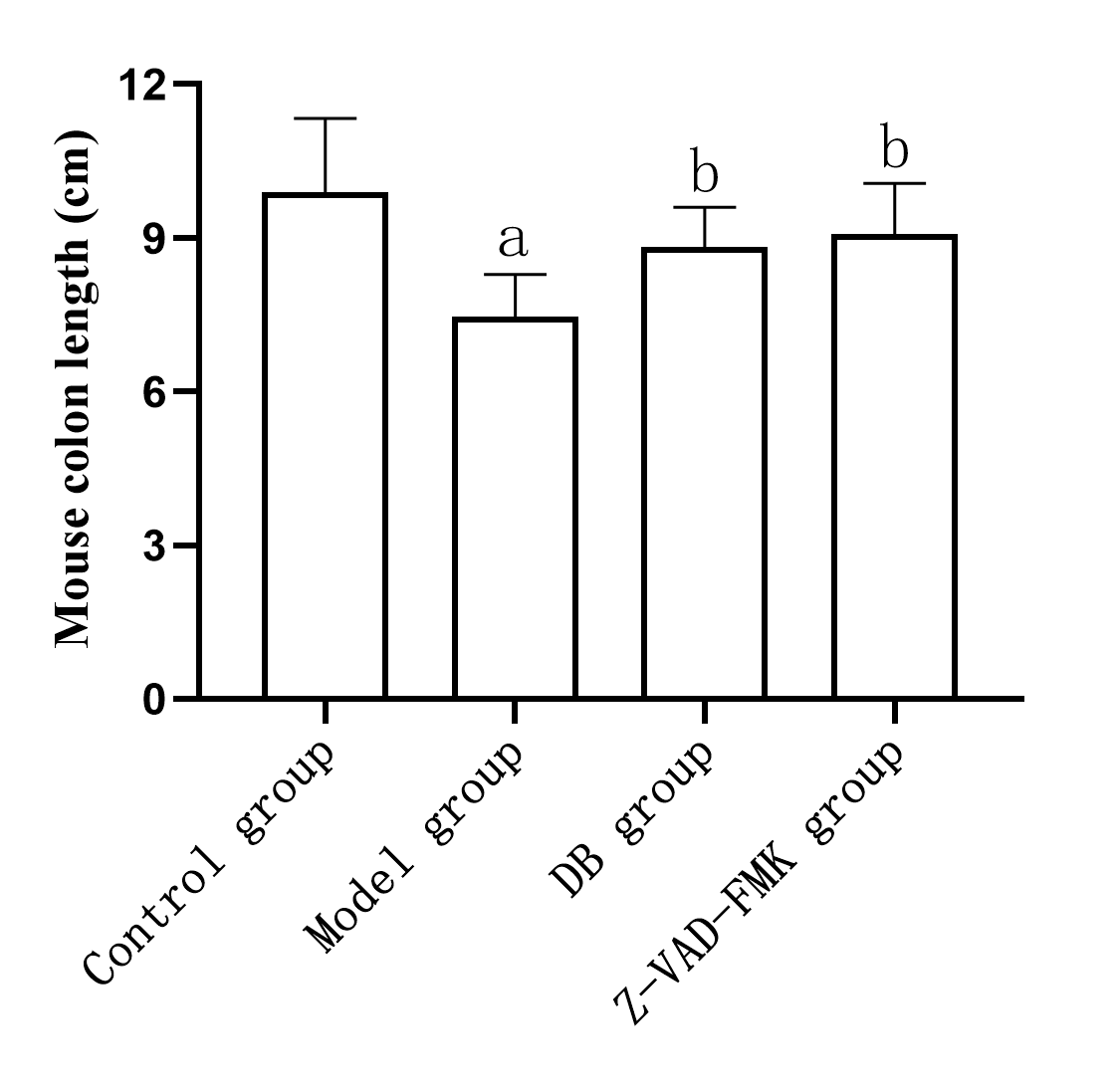

Supplement: S1 File — (ZIP) [file pone.0331570.s001.zip › Bar Chart/colon length.bmp]

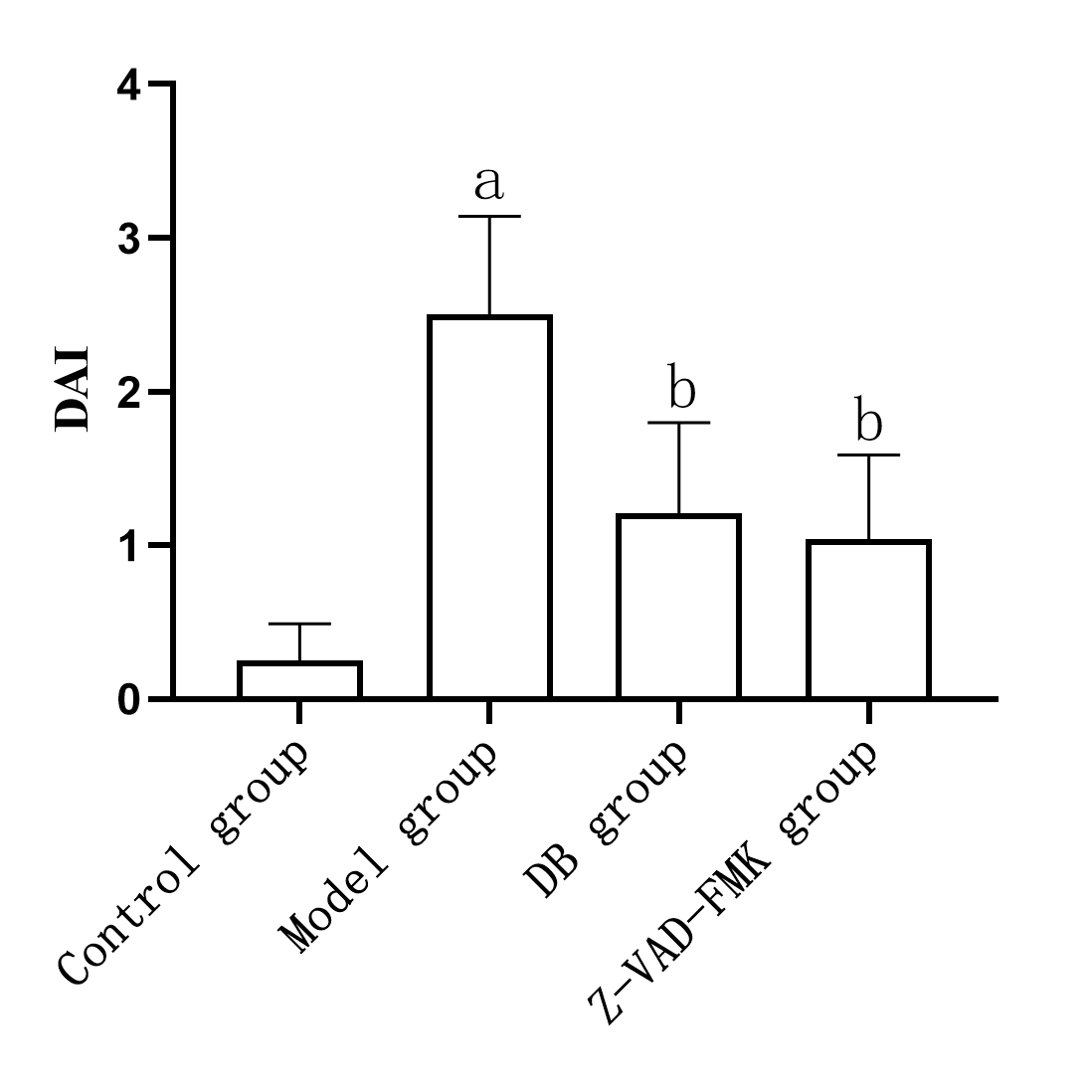

Supplement: S1 File — (ZIP) [file pone.0331570.s001.zip › Bar Chart/DAI score.bmp]

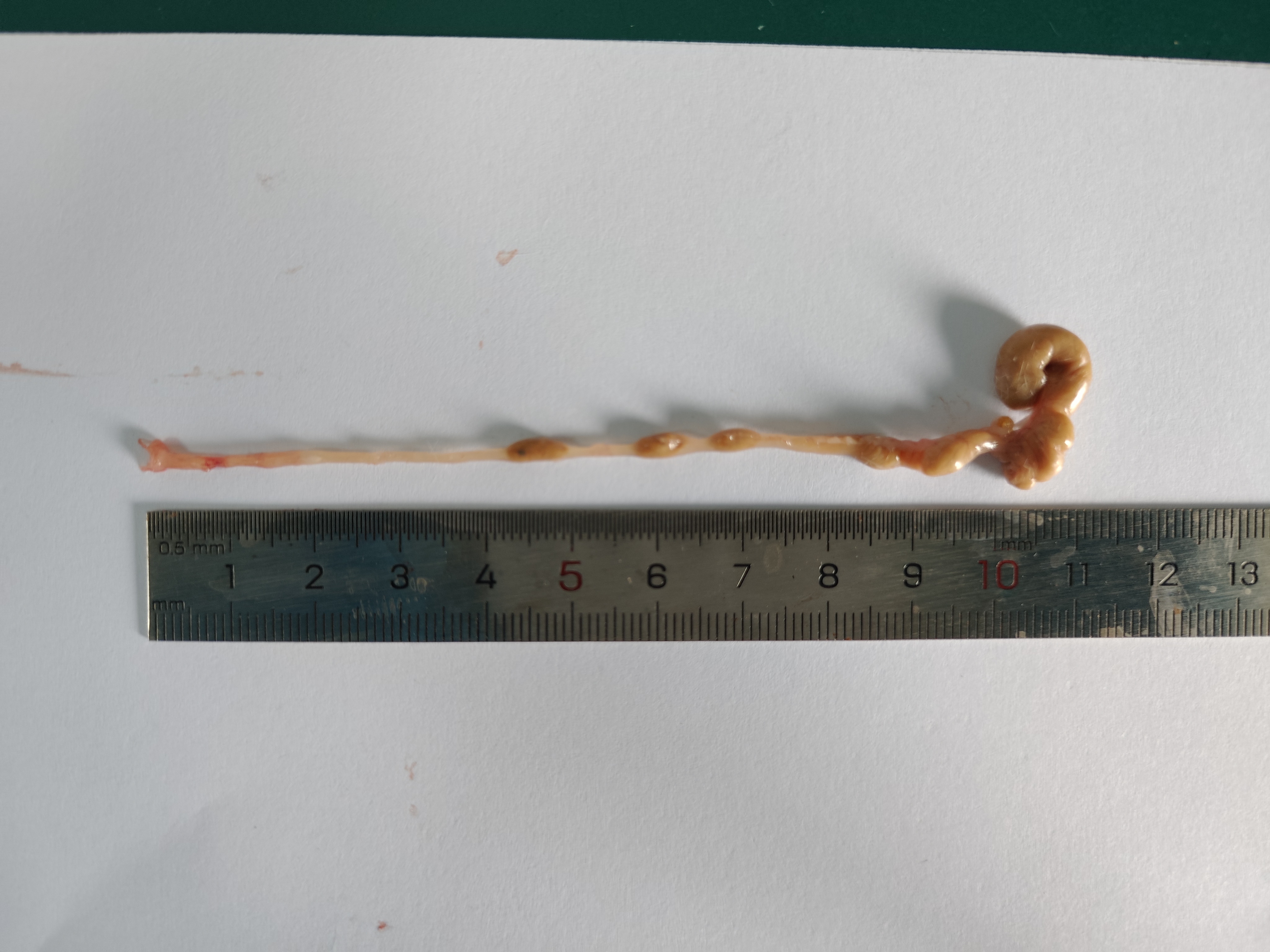

Supplement: S2 File — (ZIP) [file pone.0331570.s002.zip › Colon image/ControlGroup/1(10.1cm).jpg]

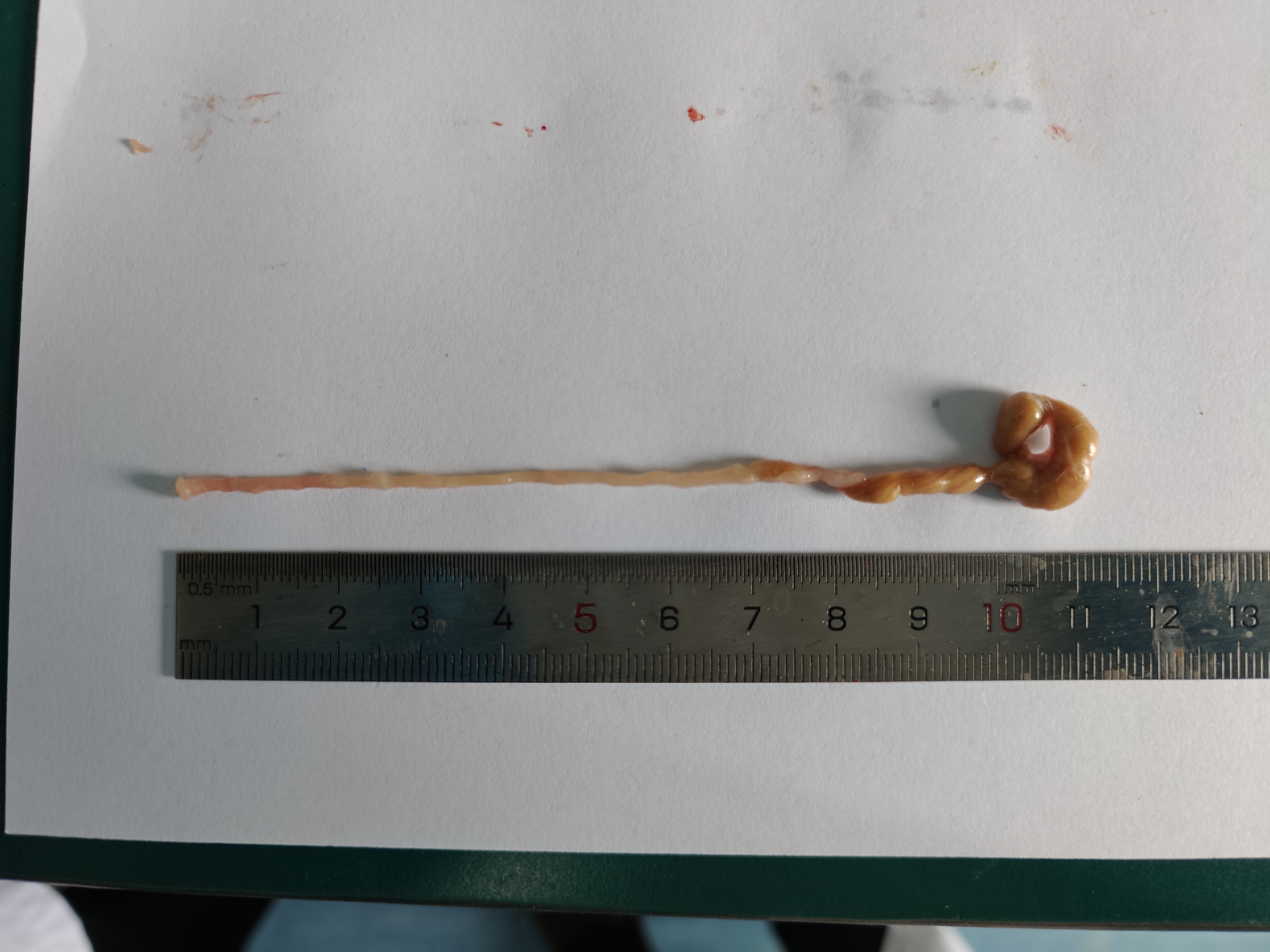

Supplement: S2 File — (ZIP) [file pone.0331570.s002.zip › Colon image/ControlGroup/2(10cm).jpg]

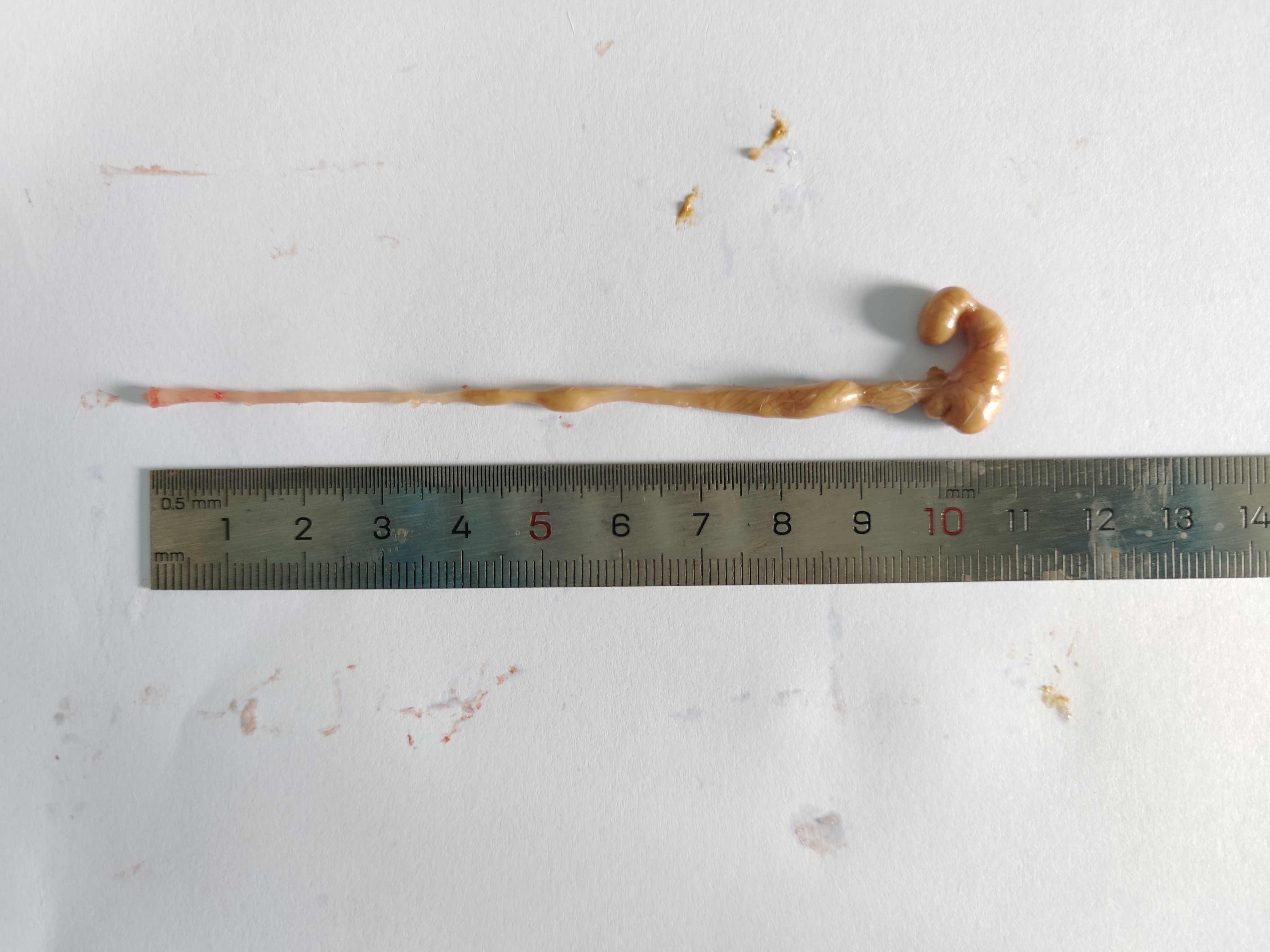

Supplement: S2 File — (ZIP) [file pone.0331570.s002.zip › Colon image/ControlGroup/3(9.8cm).jpg]

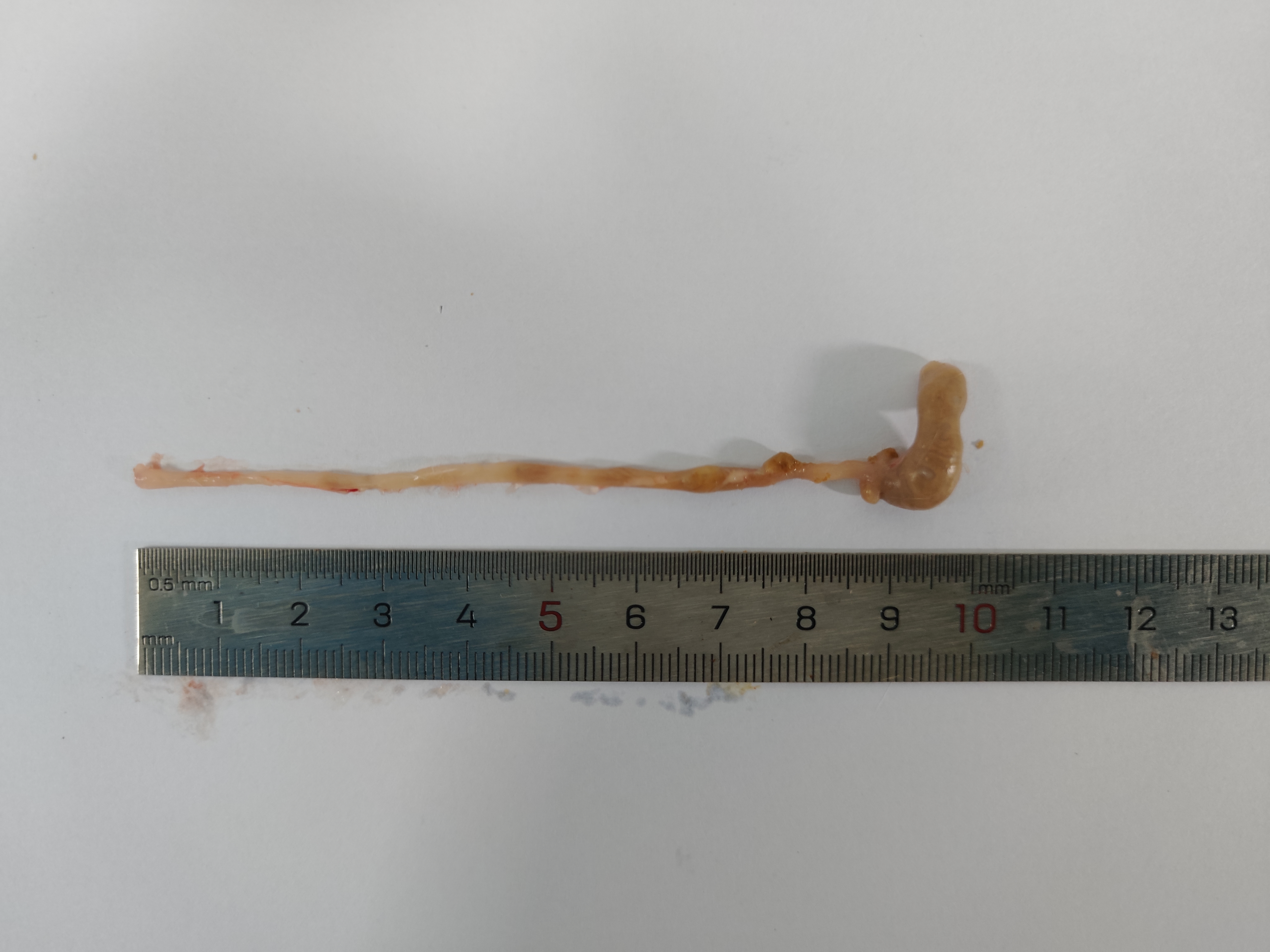

Supplement: S2 File — (ZIP) [file pone.0331570.s002.zip › Colon image/DBGroup/1(8.6cm).jpg]

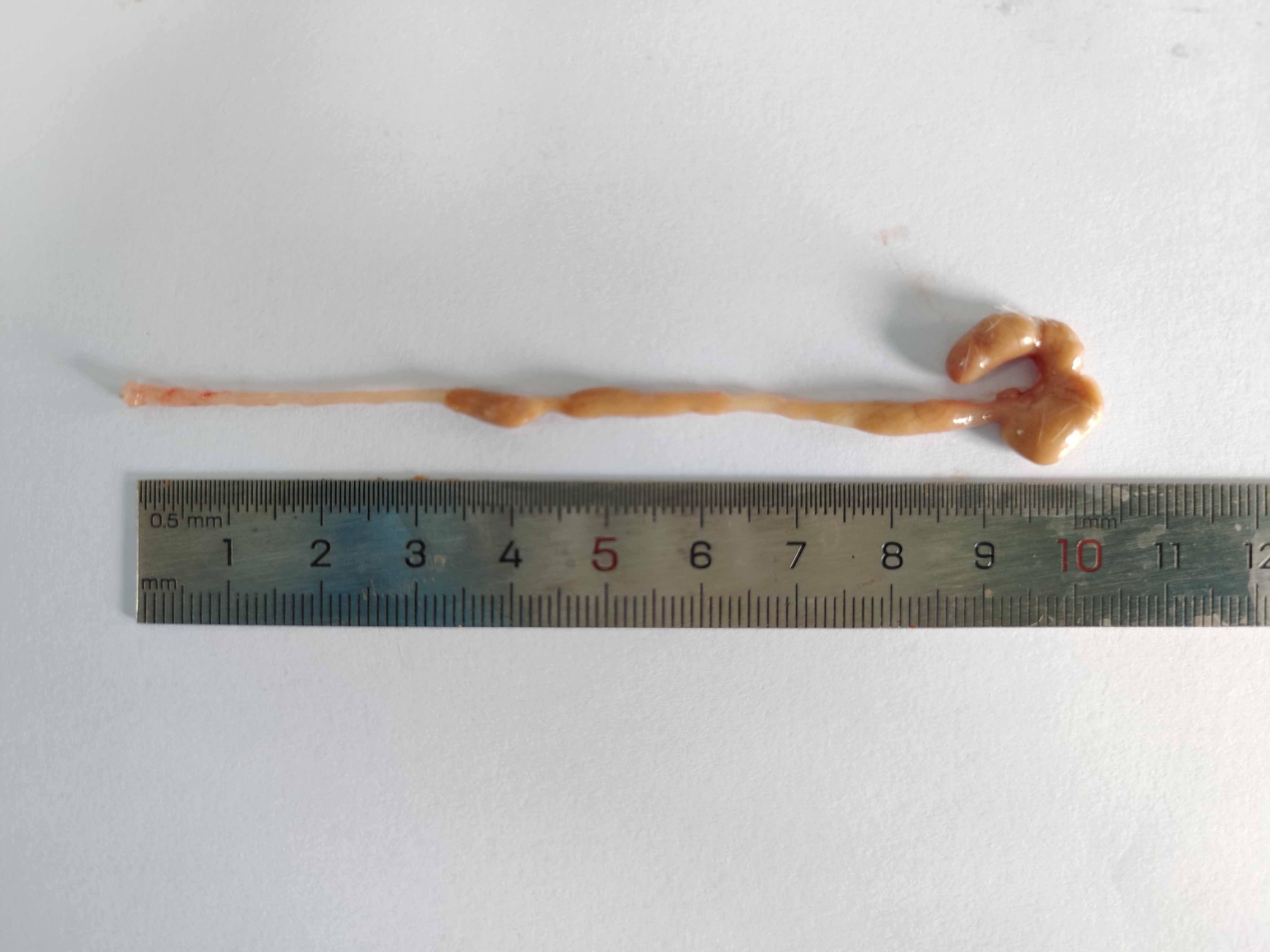

Supplement: S2 File — (ZIP) [file pone.0331570.s002.zip › Colon image/DBGroup/2(9cm).jpg]

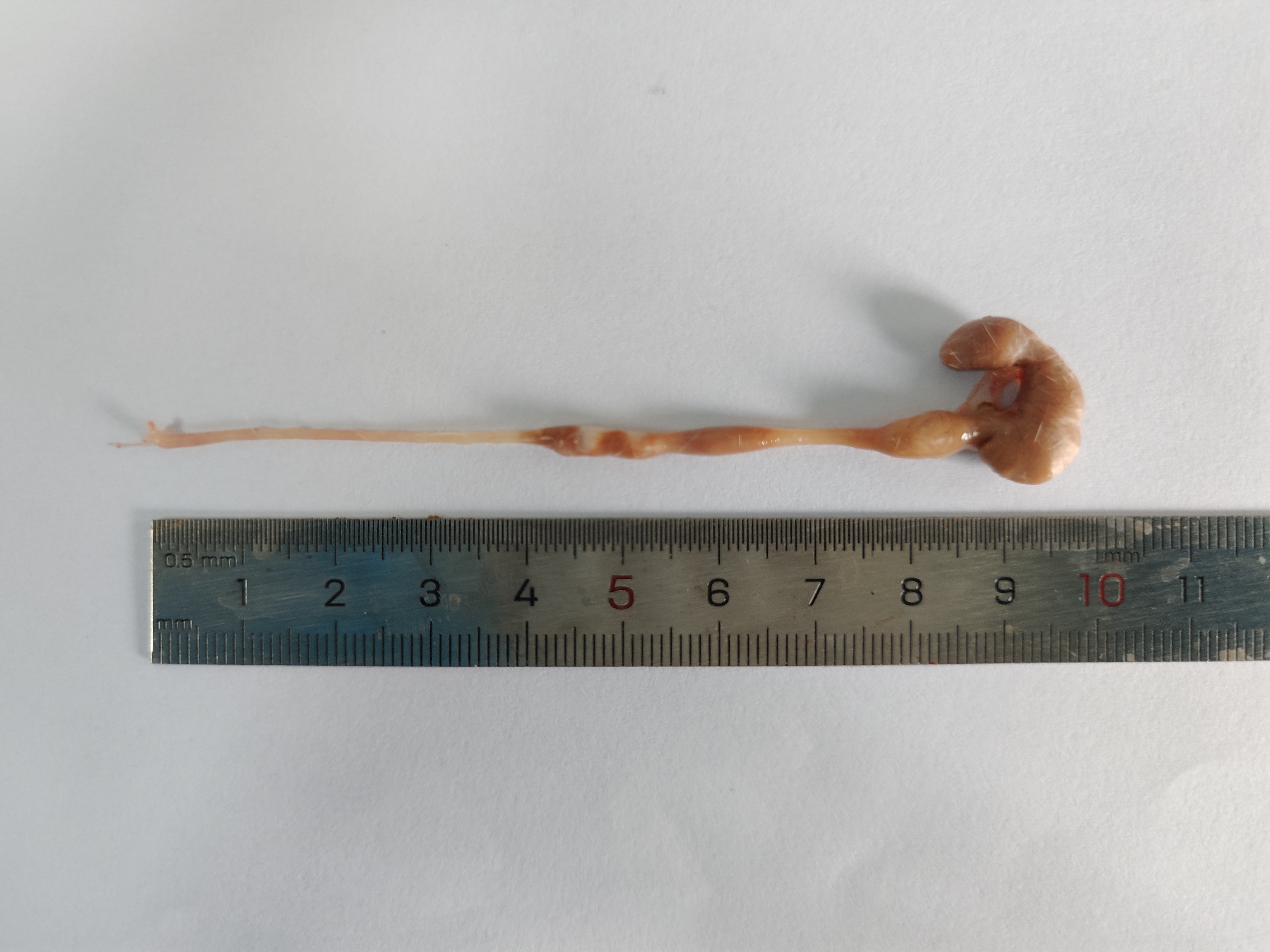

Supplement: S2 File — (ZIP) [file pone.0331570.s002.zip › Colon image/DBGroup/3(8.7cm).jpg]

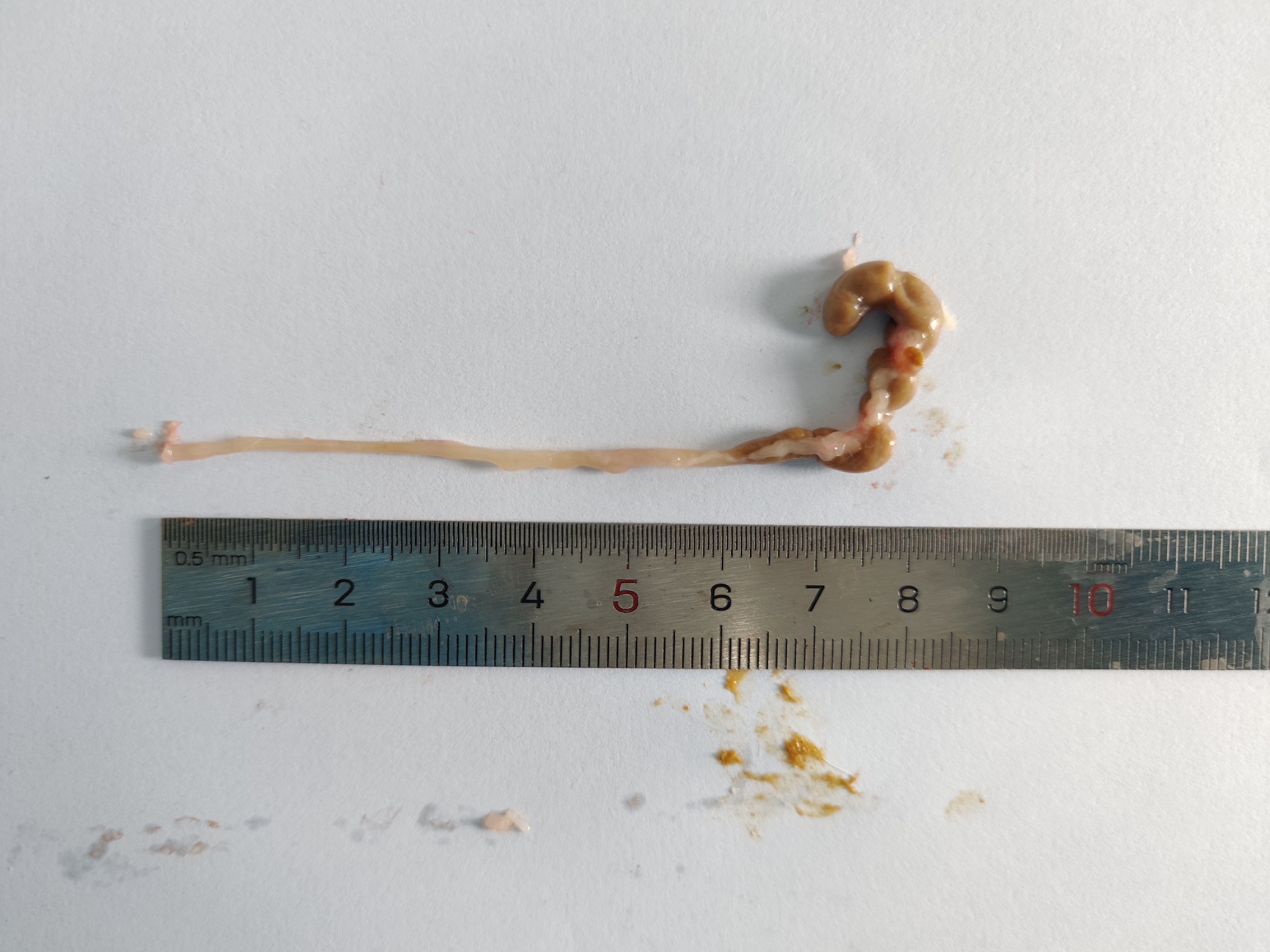

Supplement: S2 File — (ZIP) [file pone.0331570.s002.zip › Colon image/ModelGroup/1(7cm).jpg]

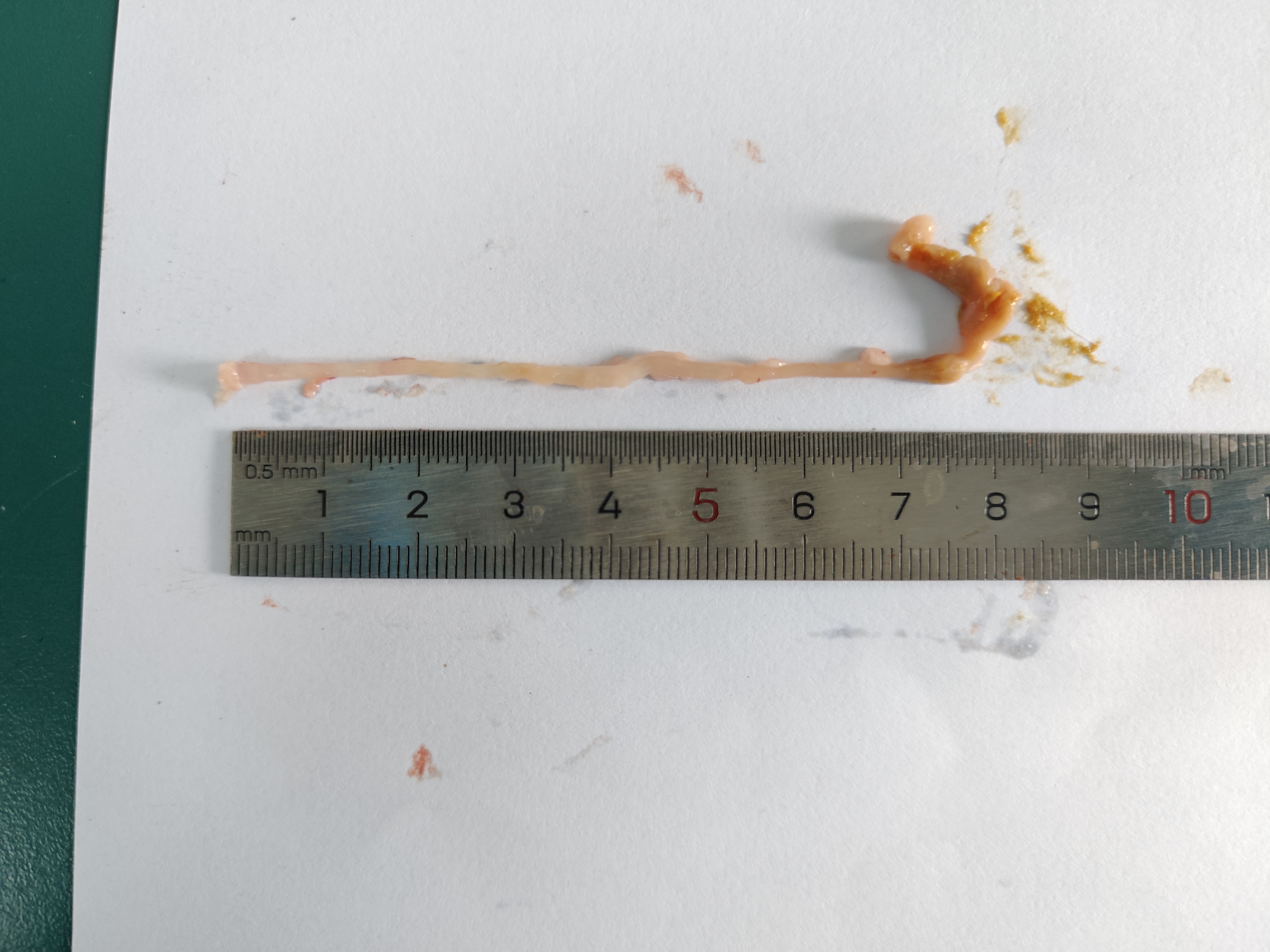

Supplement: S2 File — (ZIP) [file pone.0331570.s002.zip › Colon image/ModelGroup/2(7.5cm).jpg]

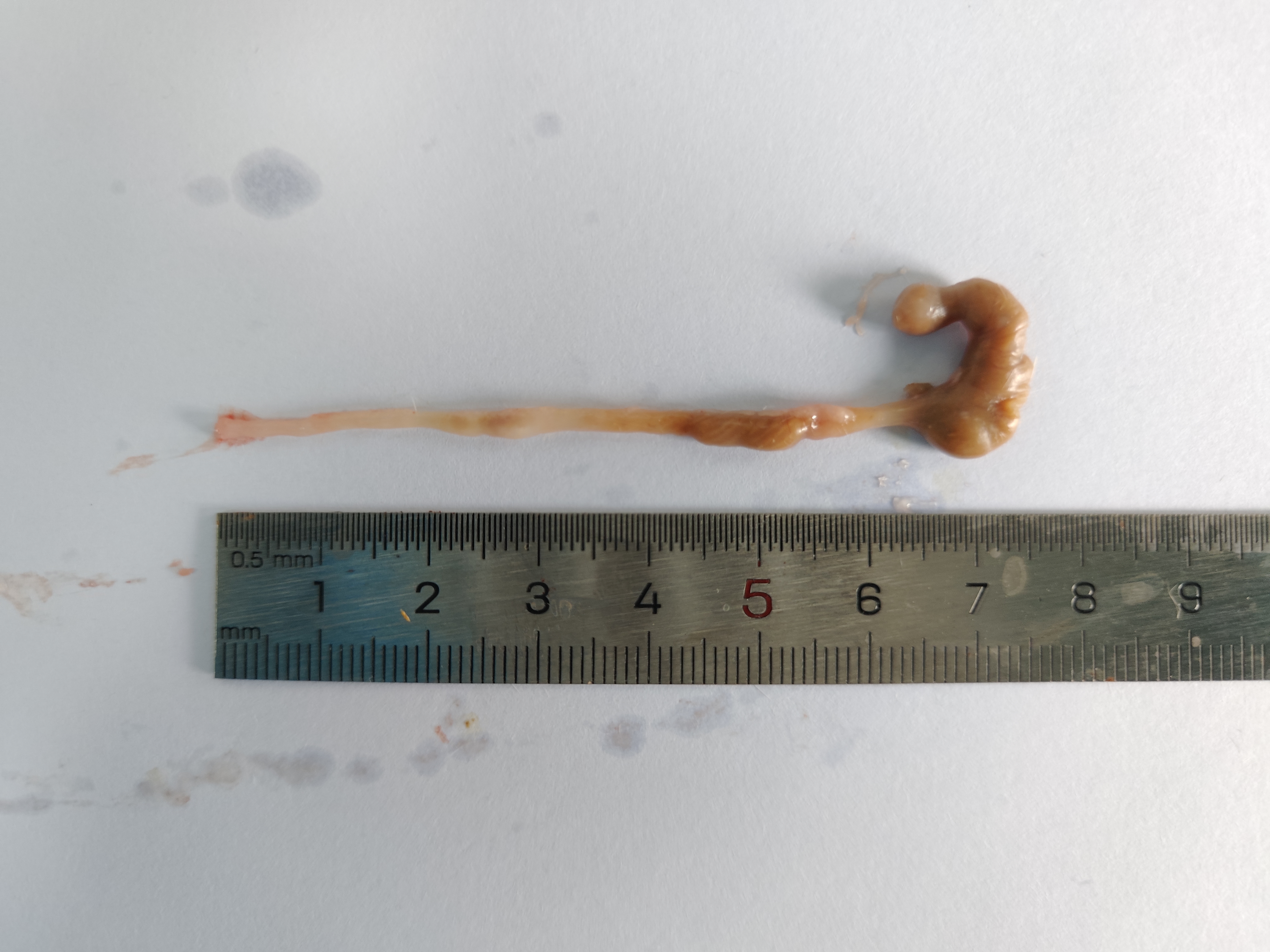

Supplement: S2 File — (ZIP) [file pone.0331570.s002.zip › Colon image/ModelGroup/3(6.5cm).jpg]

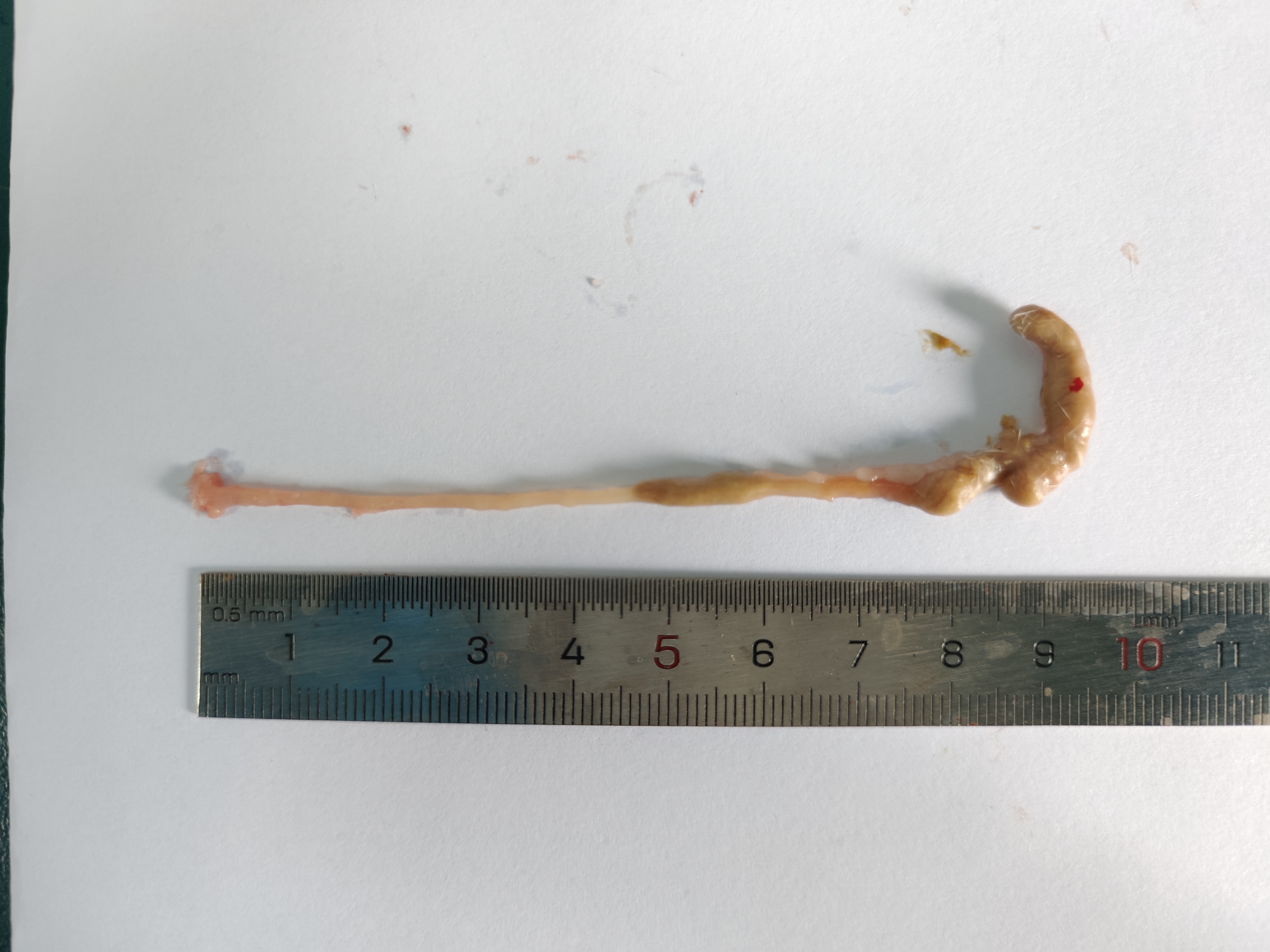

Supplement: S2 File — (ZIP) [file pone.0331570.s002.zip › Colon image/ZVADFMKGroup/1(8.6cm).jpg]

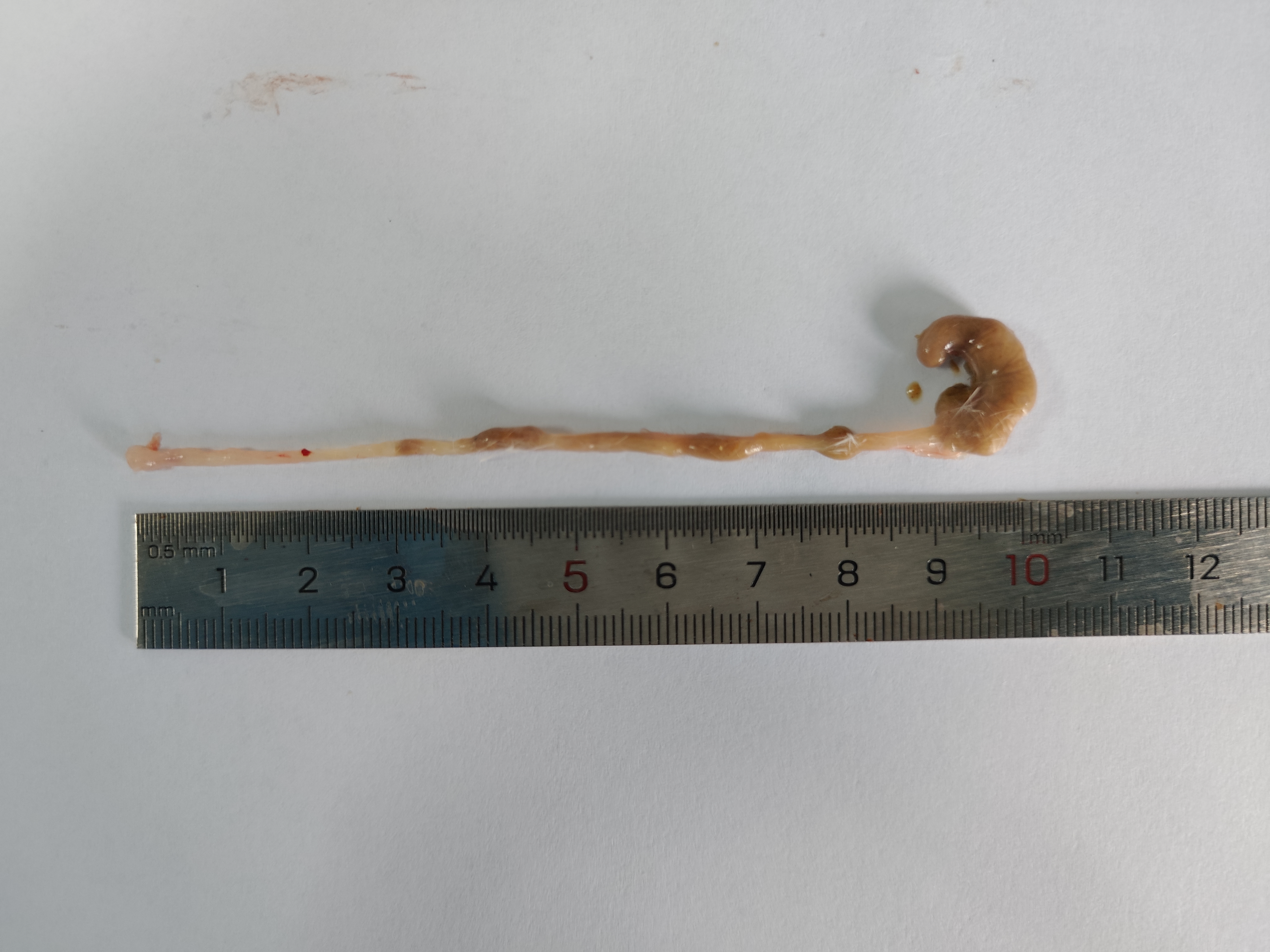

Supplement: S2 File — (ZIP) [file pone.0331570.s002.zip › Colon image/ZVADFMKGroup/2(9.2cm).jpg]

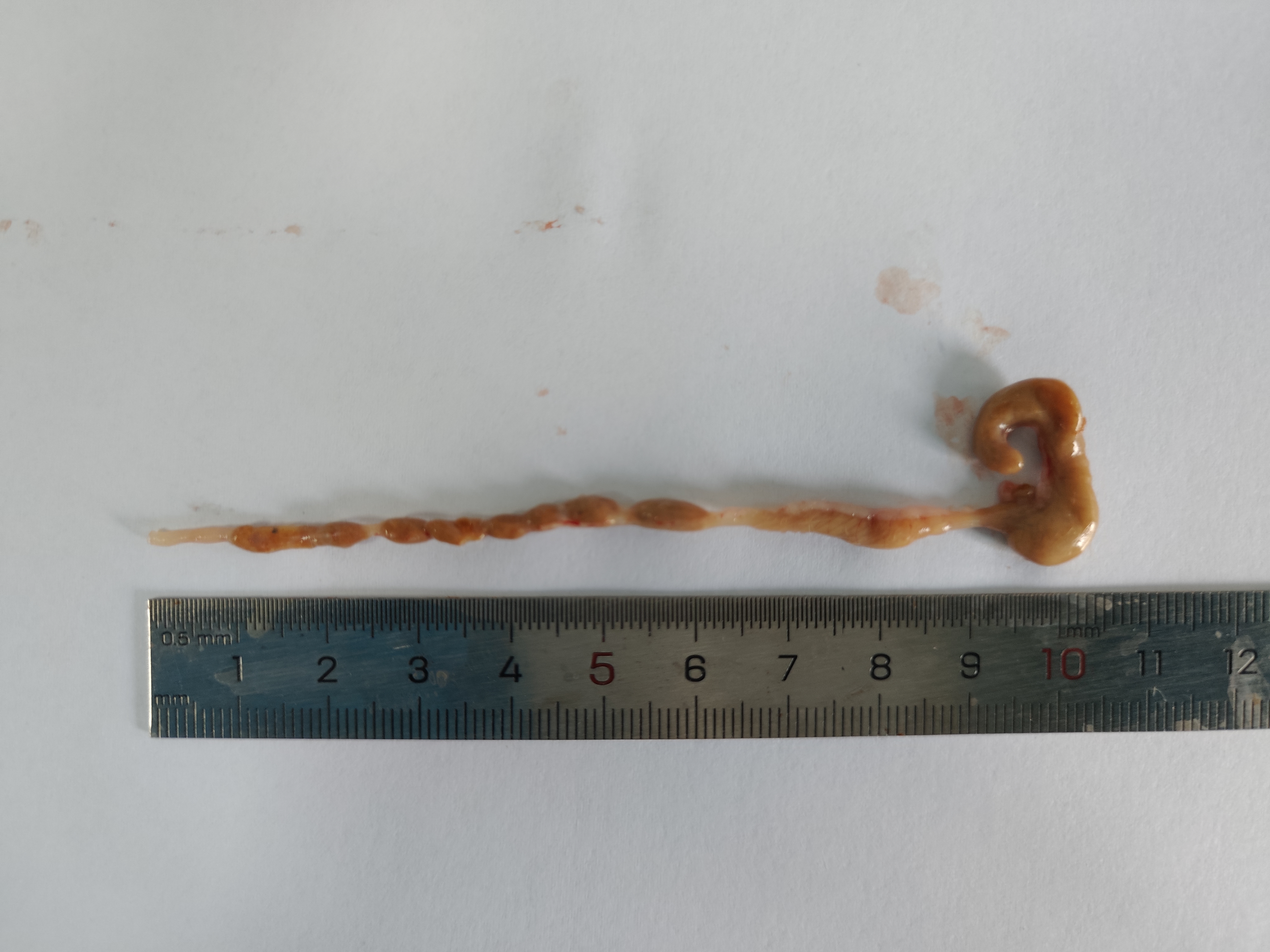

Supplement: S2 File — (ZIP) [file pone.0331570.s002.zip › Colon image/ZVADFMKGroup/3(9.4cm).jpg]

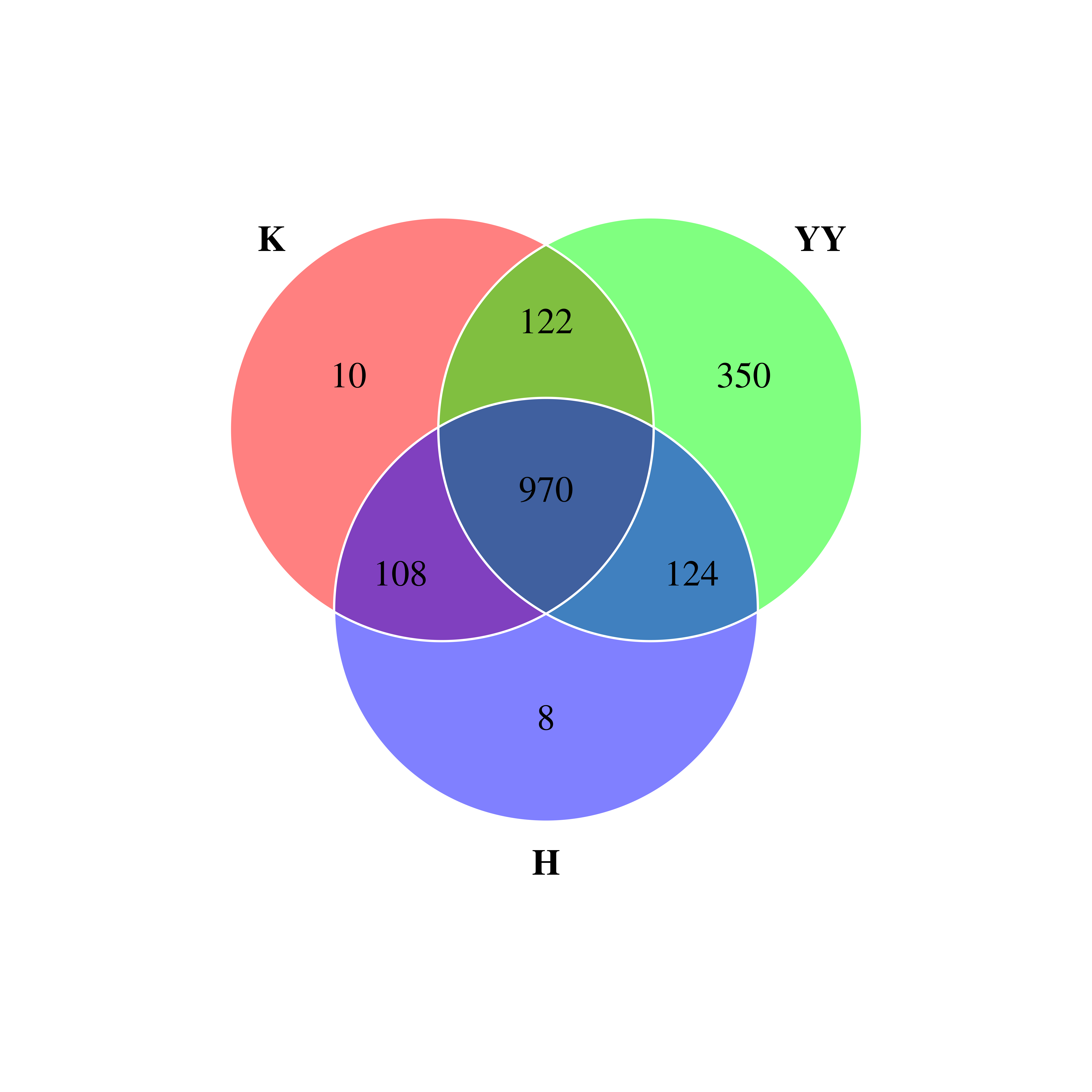

Supplement: S3 File — (ZIP) [file pone.0331570.s003.zip › Network pharmacology original diagram/Fig 2A.png]

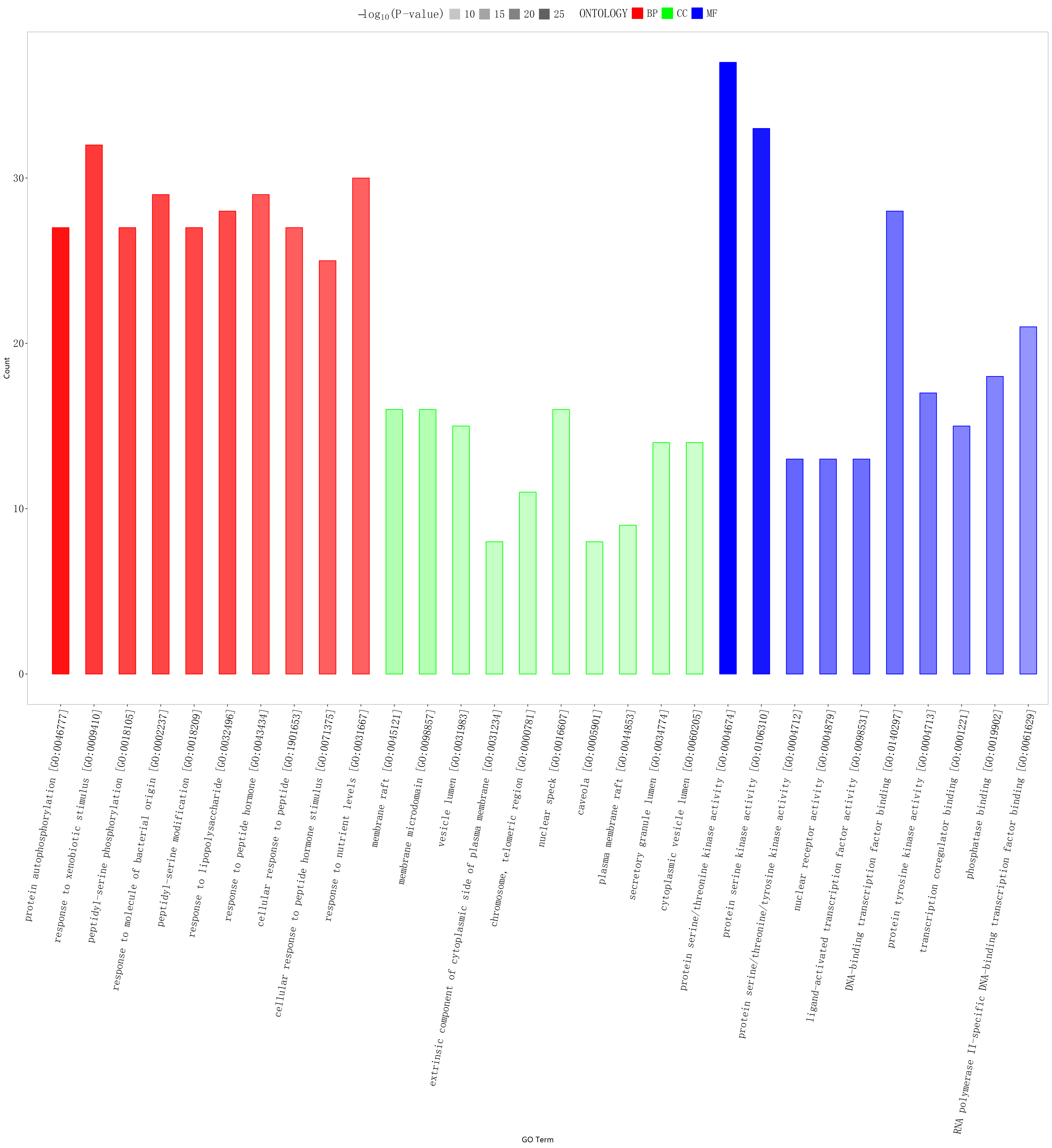

Supplement: S3 File — (ZIP) [file pone.0331570.s003.zip › Network pharmacology original diagram/Fig 2C.jpg]

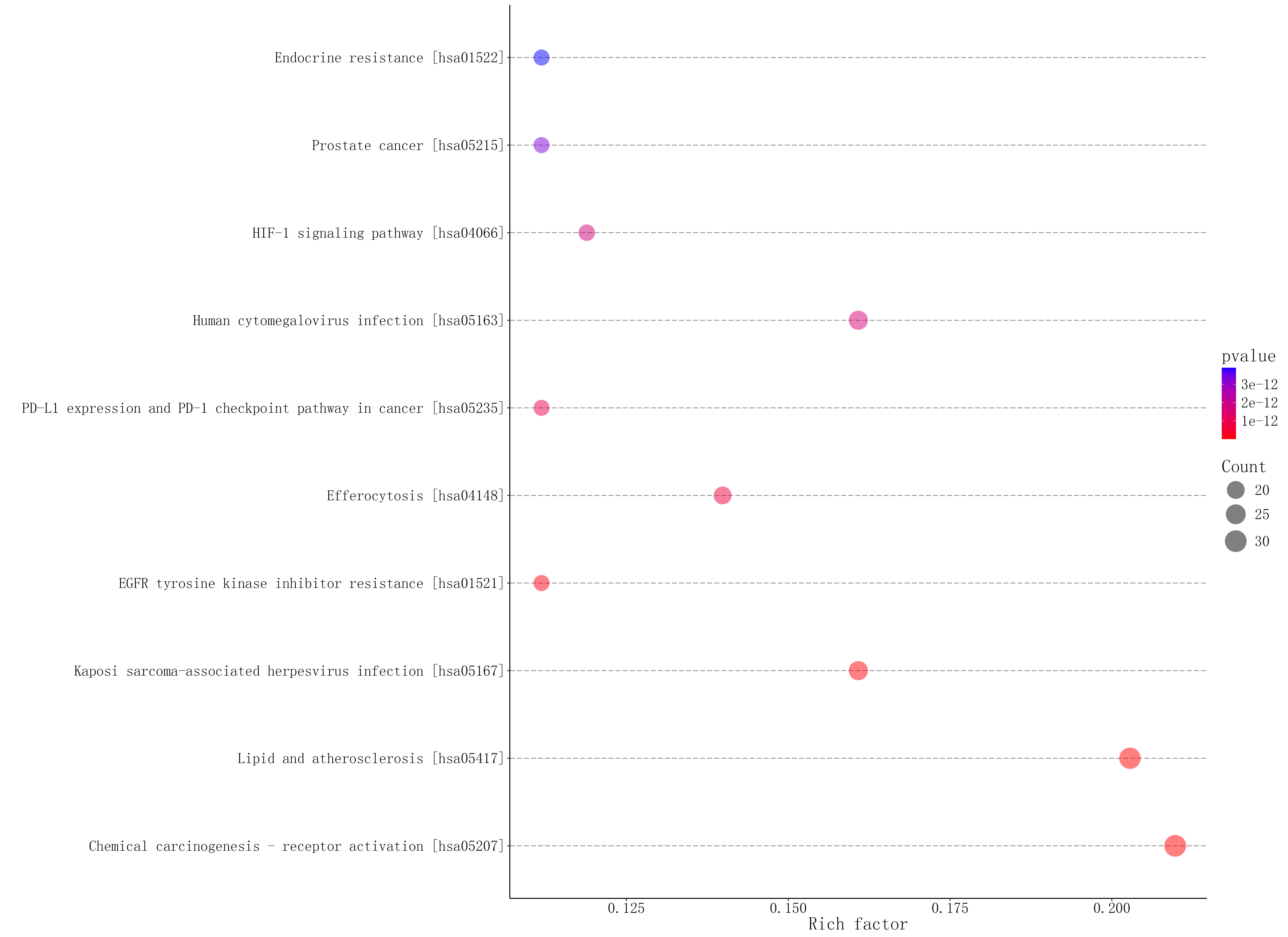

Supplement: S3 File — (ZIP) [file pone.0331570.s003.zip › Network pharmacology original diagram/Fig 2D.jpg]

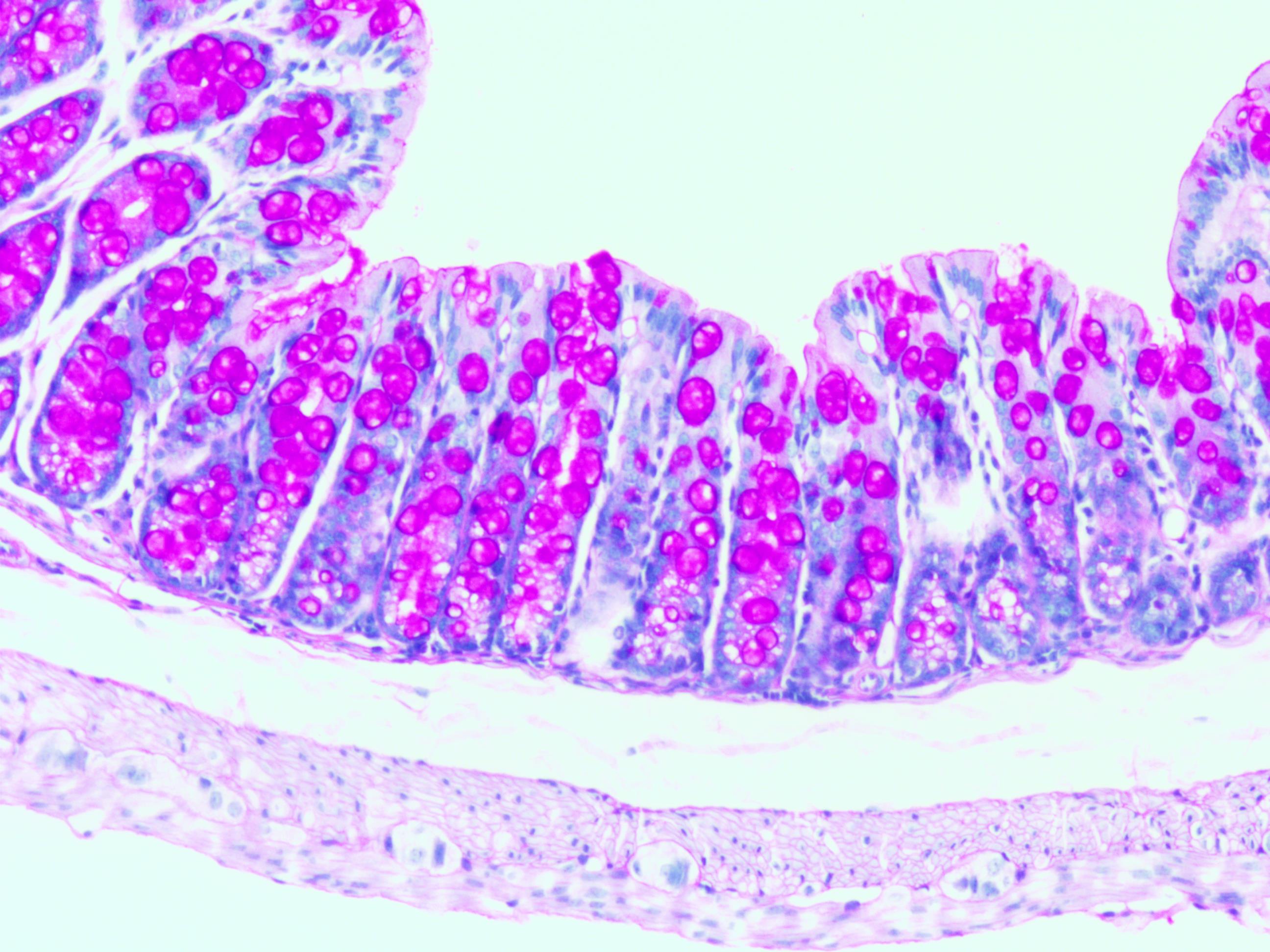

Supplement: S4 File — (ZIP) [file pone.0331570.s004.zip › Original image selected by PAS/ControlGroup100x.bmp]

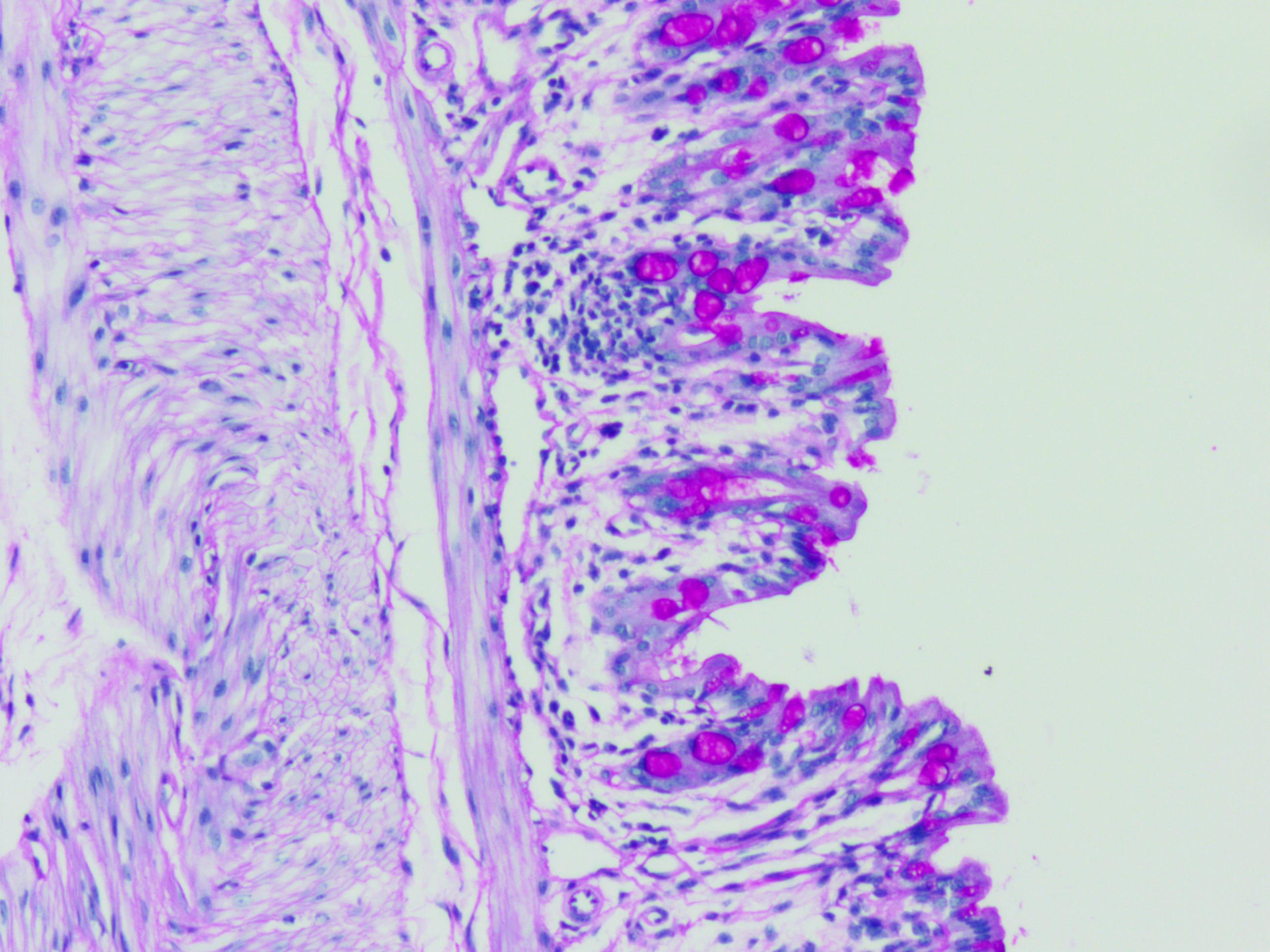

Supplement: S4 File — (ZIP) [file pone.0331570.s004.zip › Original image selected by PAS/DBGroup100x.bmp]

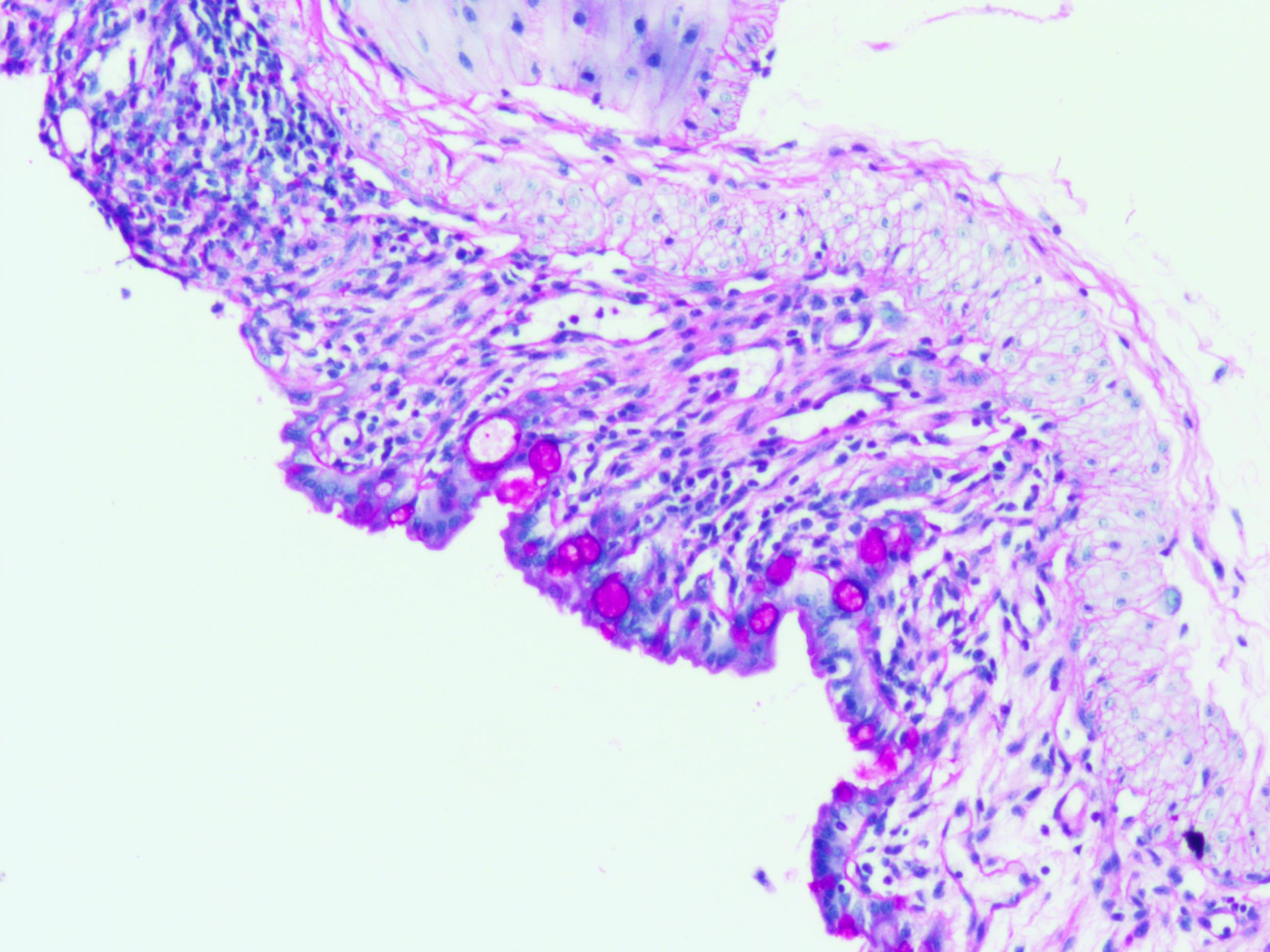

Supplement: S4 File — (ZIP) [file pone.0331570.s004.zip › Original image selected by PAS/ModelGroup100x.bmp]

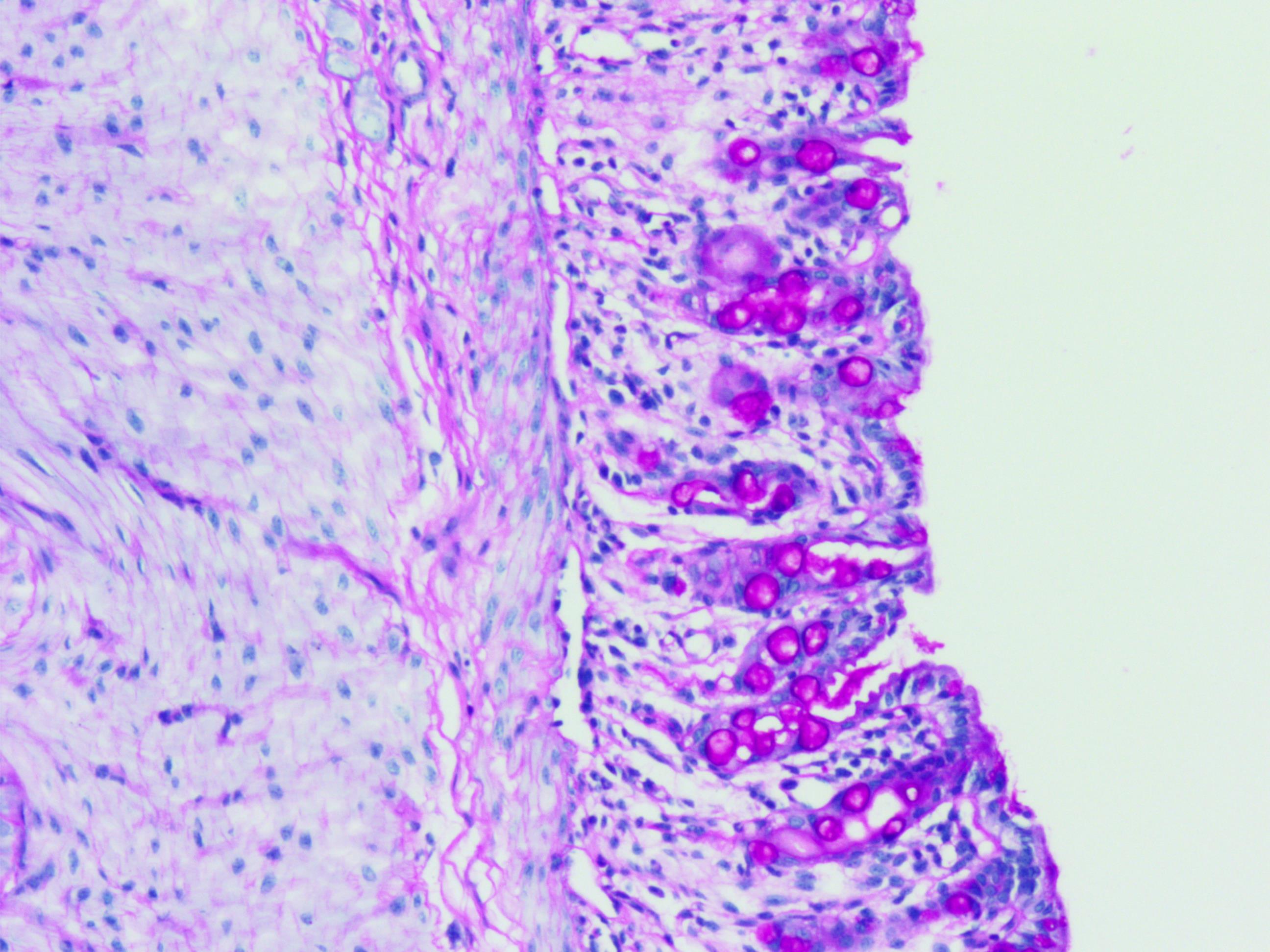

Supplement: S4 File — (ZIP) [file pone.0331570.s004.zip › Original image selected by PAS/ZVADFMKGroup 100x.bmp]

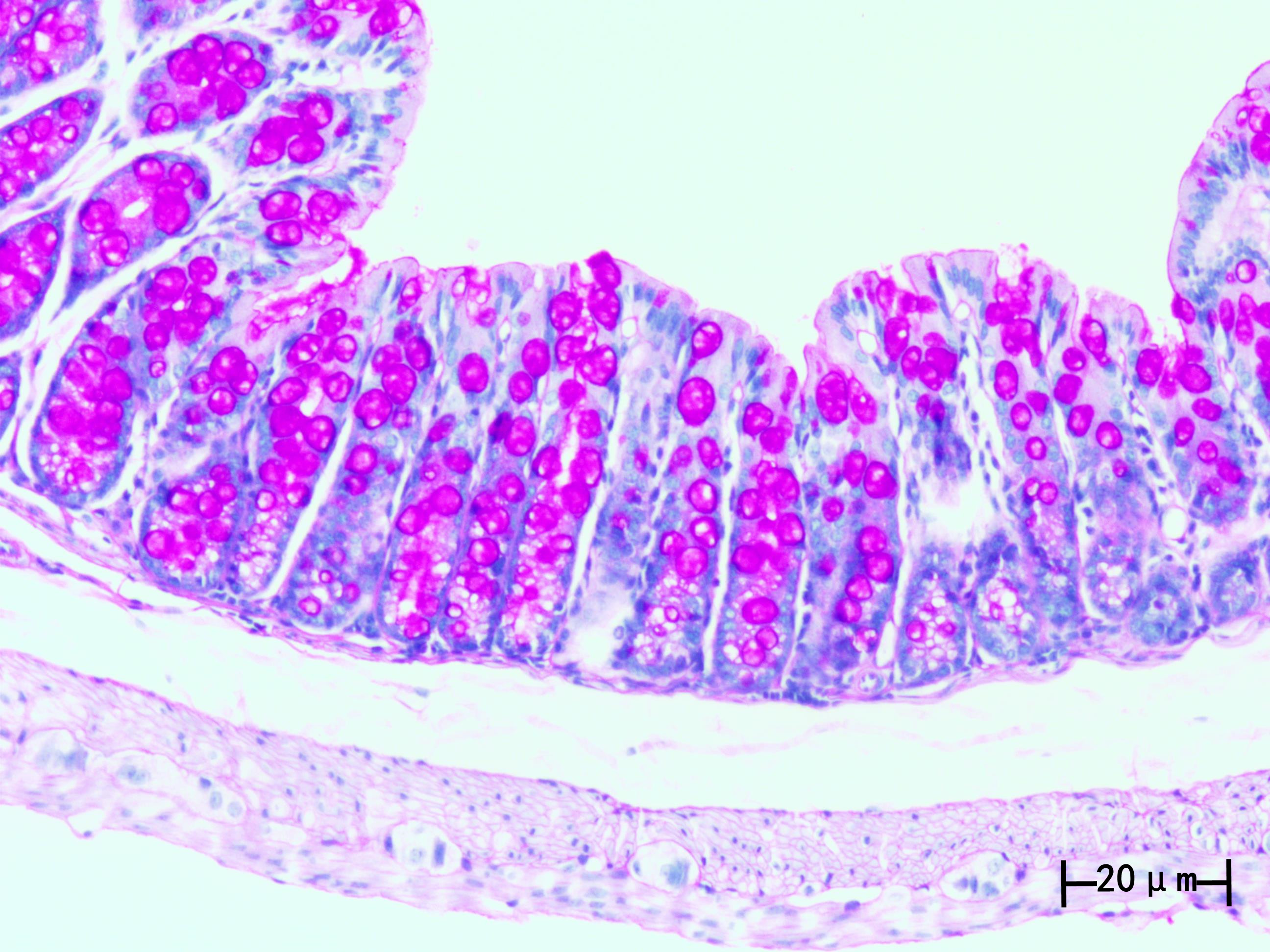

Supplement: S5 File — (ZIP) [file pone.0331570.s005.zip › PAS selected marked scale image/ControlGroup.jpg]

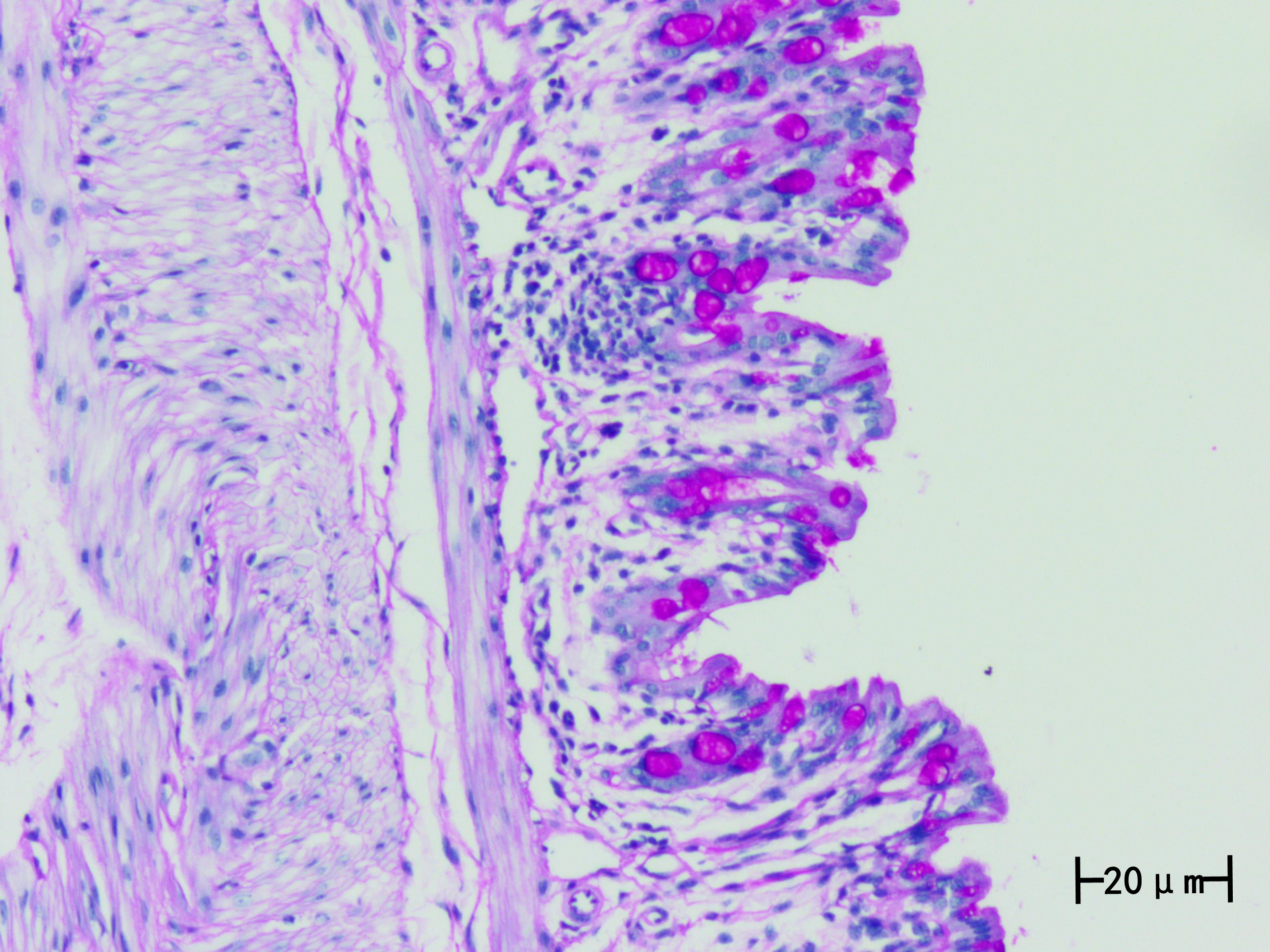

Supplement: S5 File — (ZIP) [file pone.0331570.s005.zip › PAS selected marked scale image/DBGroup.jpg]

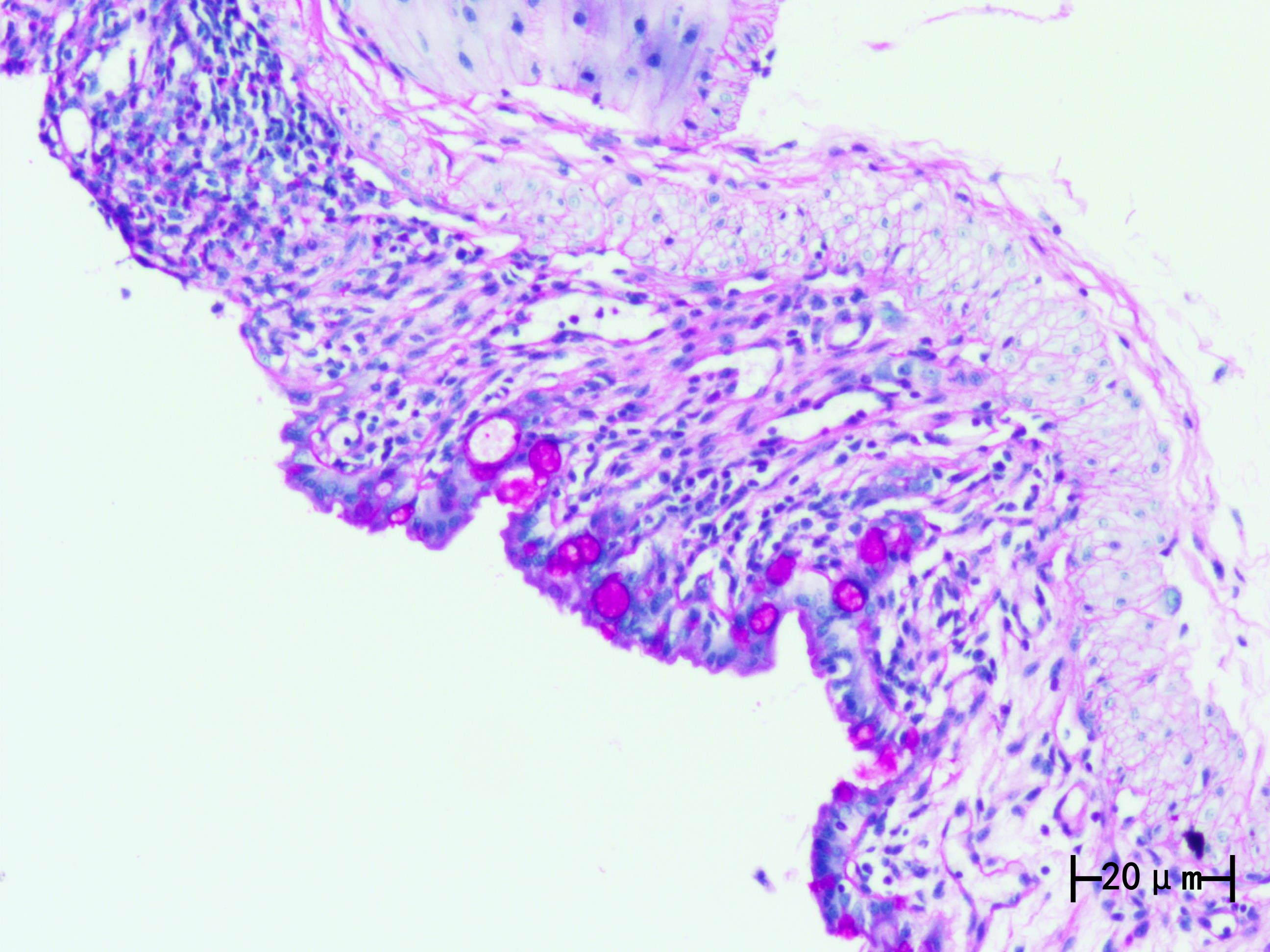

Supplement: S5 File — (ZIP) [file pone.0331570.s005.zip › PAS selected marked scale image/ModelGroup.jpg]

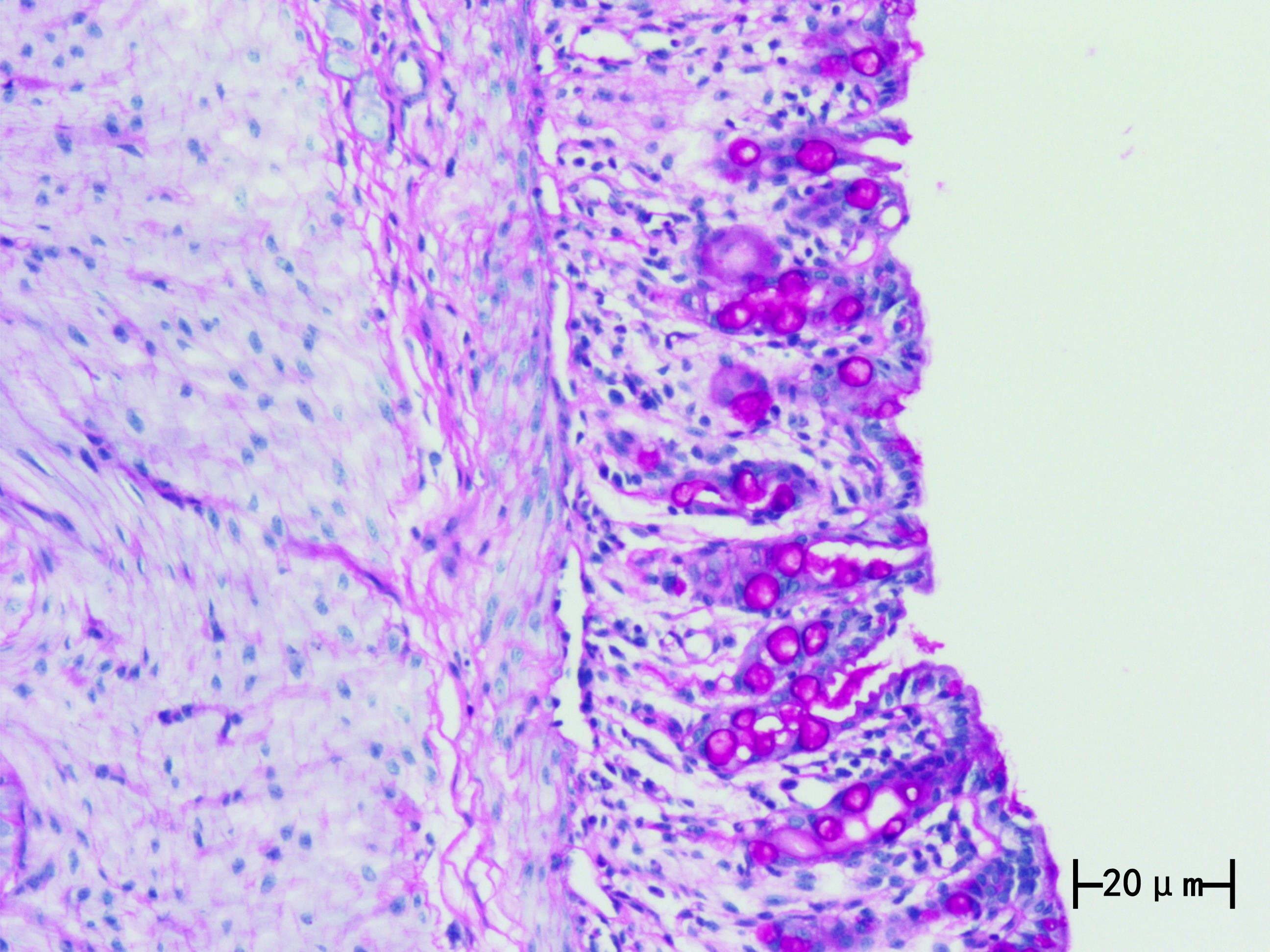

Supplement: S5 File — (ZIP) [file pone.0331570.s005.zip › PAS selected marked scale image/ZVADFMKGroup .jpg]

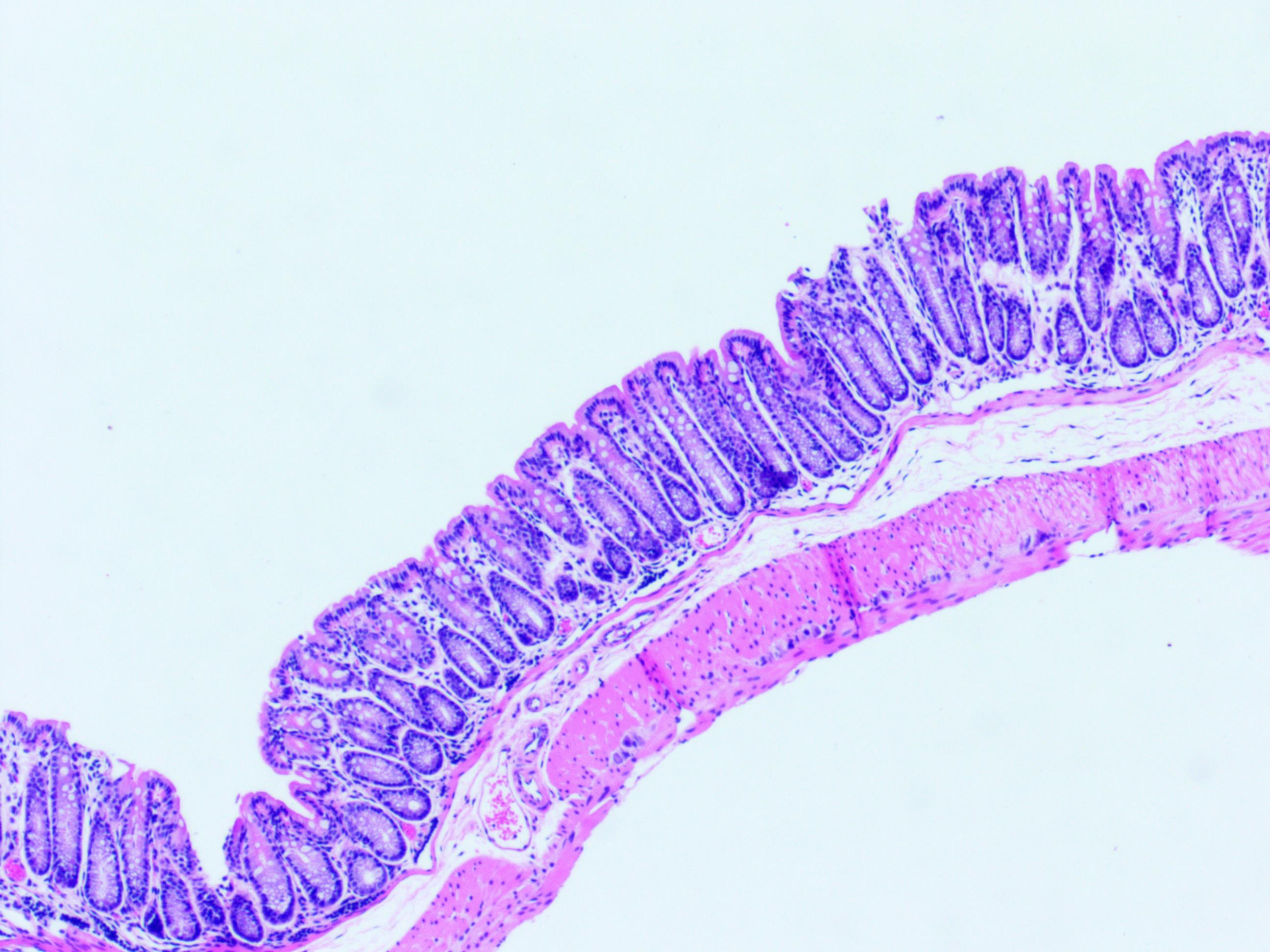

Supplement: S7 File — (ZIP) [file pone.0331570.s007.zip › Ulcerative colitis HE image 40x magnification/ControlGroup.bmp]

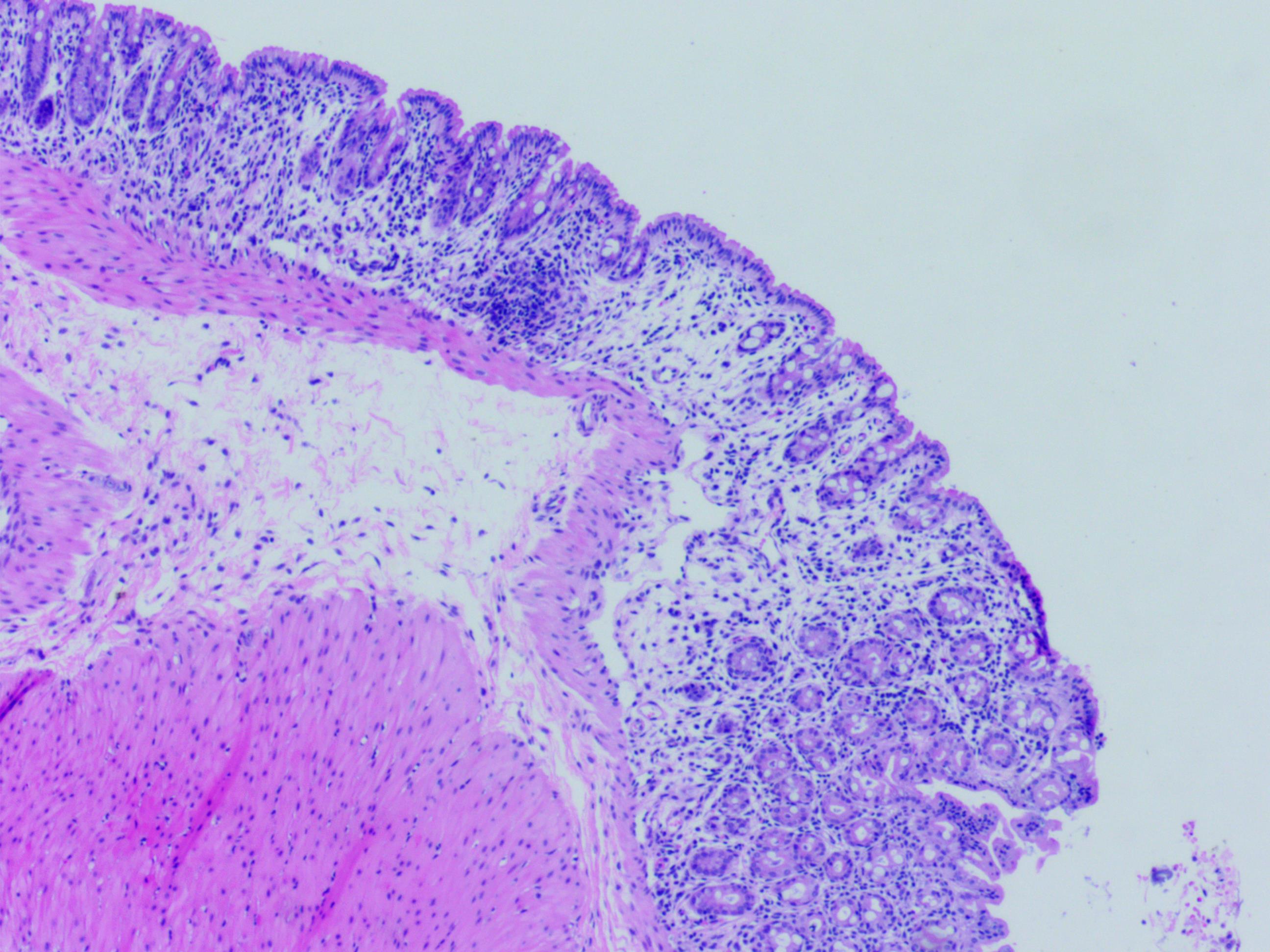

Supplement: S7 File — (ZIP) [file pone.0331570.s007.zip › Ulcerative colitis HE image 40x magnification/DBGroup.bmp]

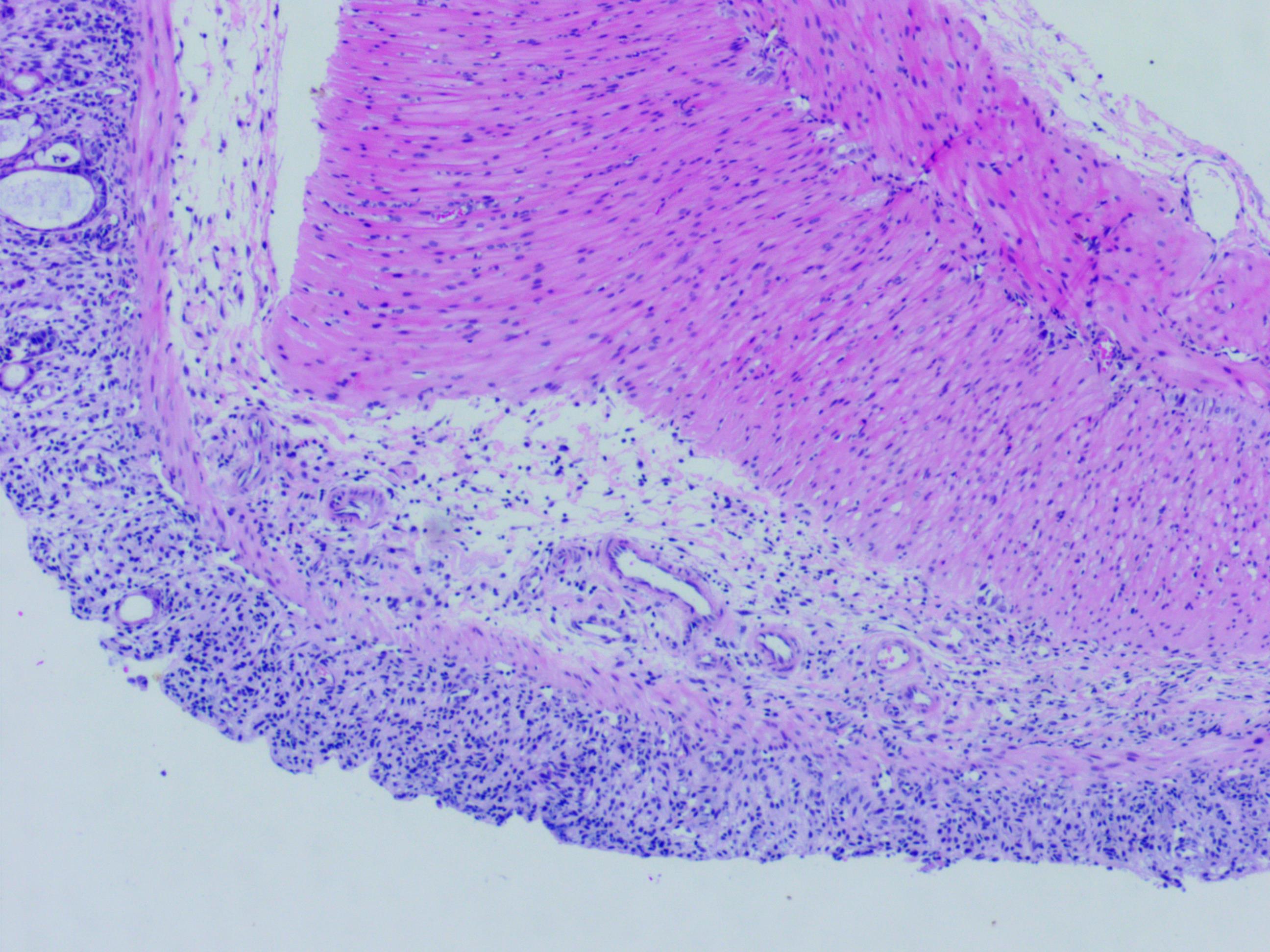

Supplement: S7 File — (ZIP) [file pone.0331570.s007.zip › Ulcerative colitis HE image 40x magnification/ModelGroup.bmp]

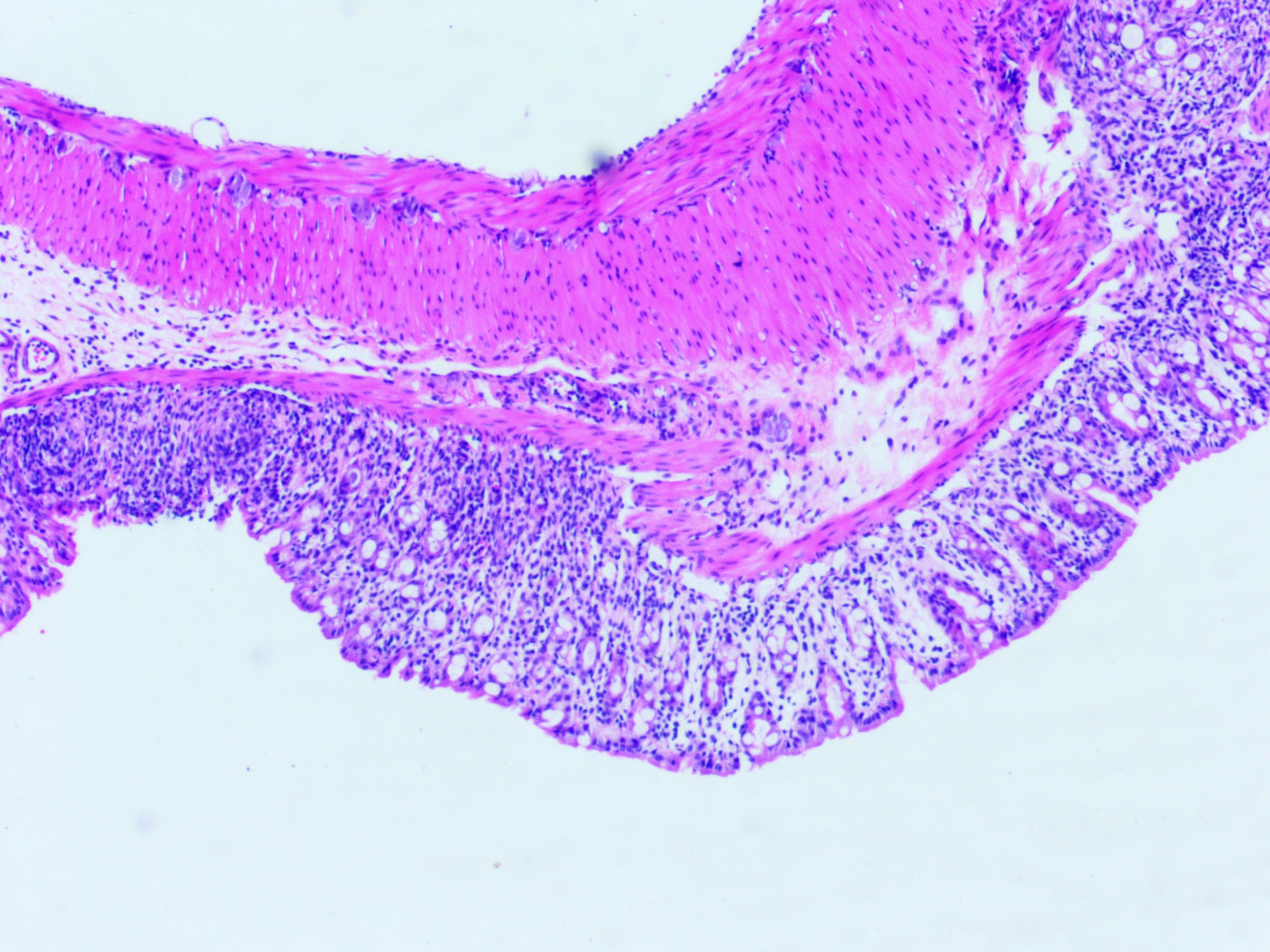

Supplement: S7 File — (ZIP) [file pone.0331570.s007.zip › Ulcerative colitis HE image 40x magnification/ZVADFMKGroup .bmp]
