# Supplementary material for: Molecular mechanisms of dragon’s blood in treating ulcerative colitis based on NF-κb/NLPR3/Caspase-1 pyroptosis signaling pathway
Source: PLoS One. 2025 Sep 19;20(9):e0331570. doi: 10.1371/journal.pone.0331570 (PMC12448351; doi:10.1371/journal.pone.0331570)
Supplement: S6 File — (PDF) [file pone.0331570.s006.pdf]

Caspase1 Blot 1

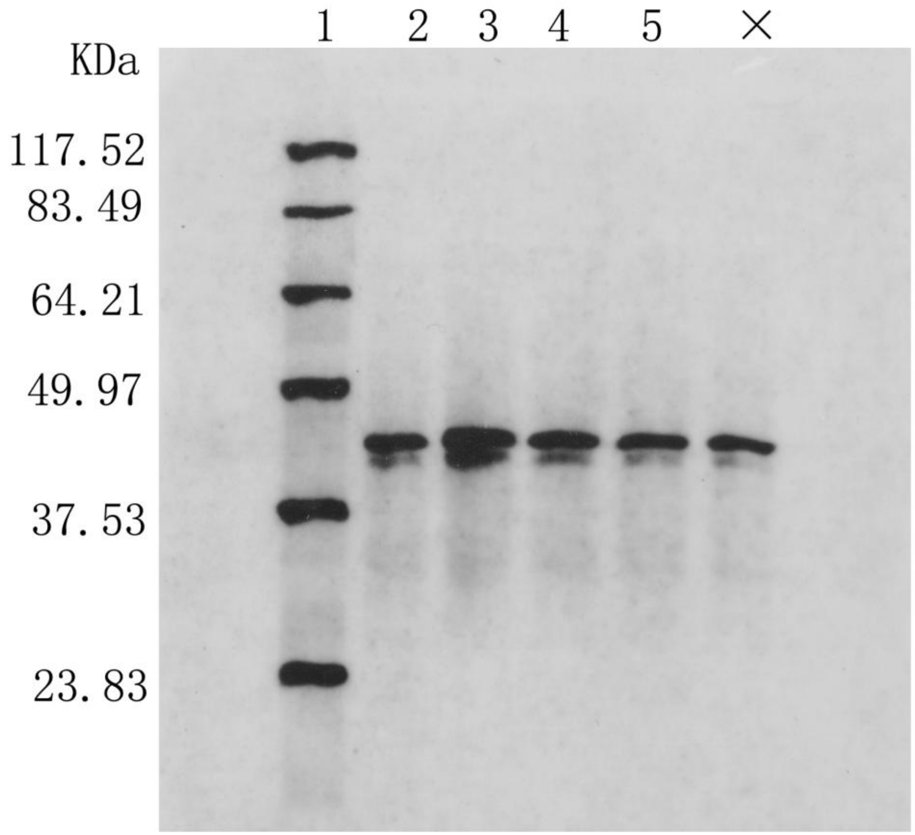

Lane 1:Marker

Lane 2:Control Group

Lane 3:Model Group

Lane 4:DB Group

Lane 5:Z-VAD-FMK Group

Caspase1 Blot 2

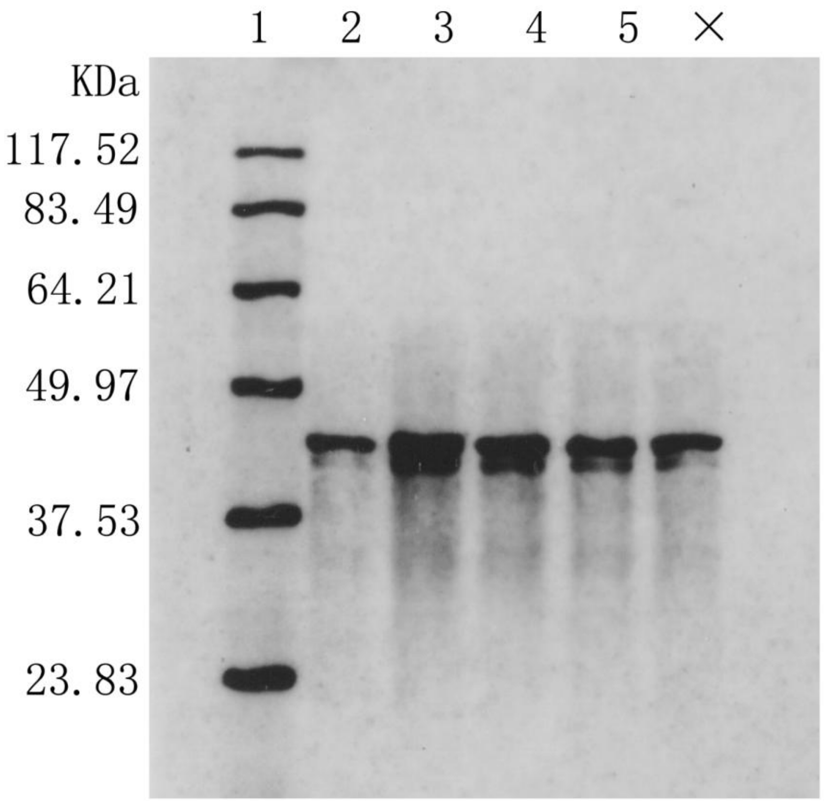

Lane 1:Marker

Lane 2:Control Group

Lane 3:Model Group

Lane 4:DB Group

Lane 5:Z-VAD-FMK Group

Caspase1 Blot 3

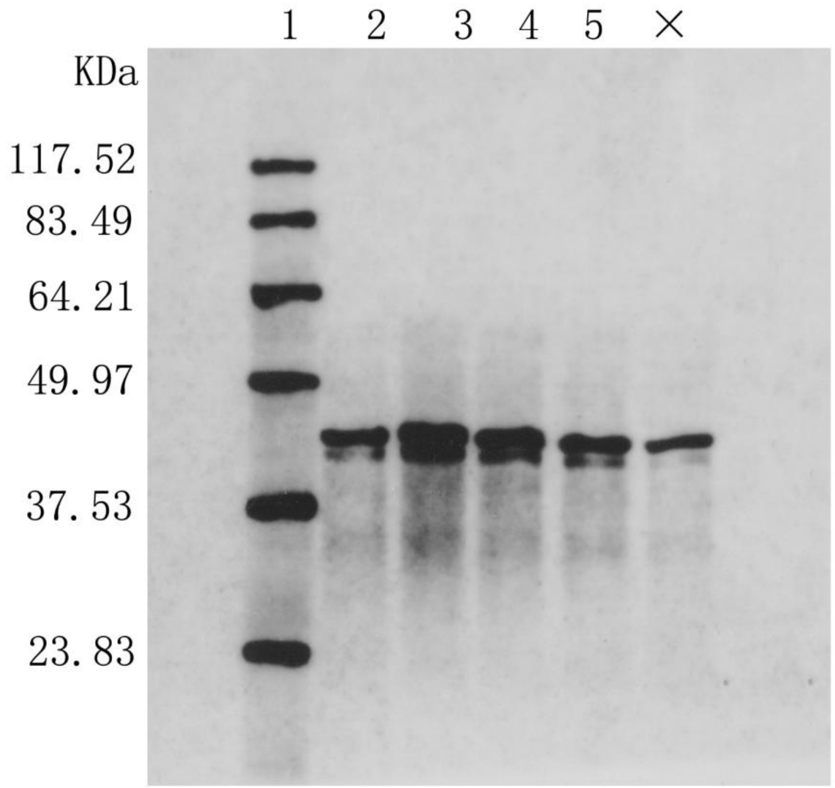

Lane 1:Marker

Lane 2:Control Group

Lane 3:Model Group

Lane 4:DB Group

Lane 5:Z-VAD-FMK Group

IL-1β Blot 1

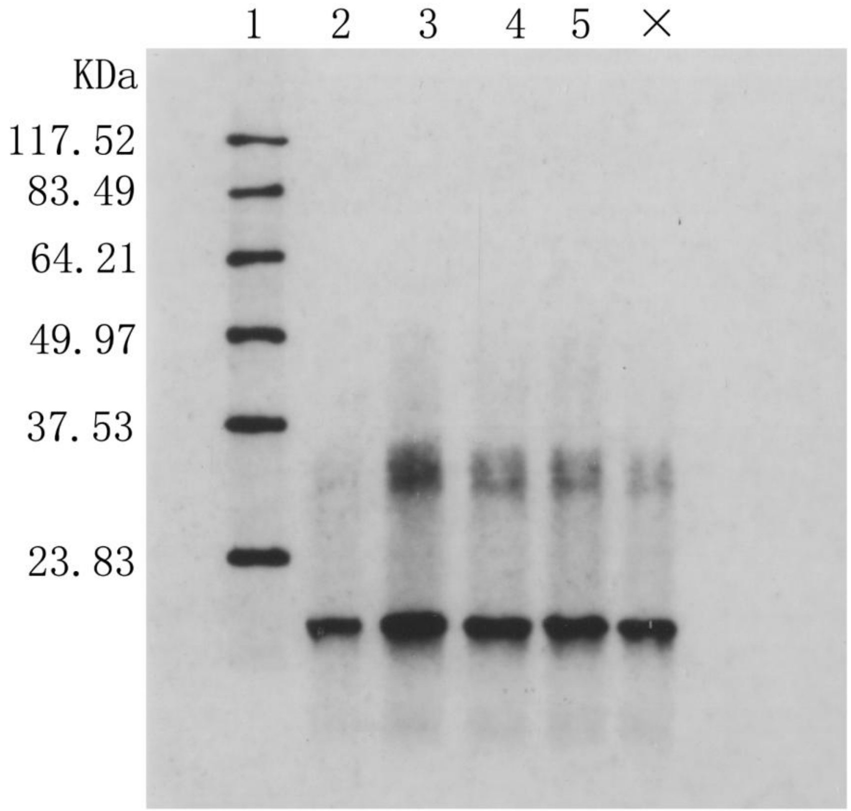

Lane 1:Marker

Lane 2:Control Group

Lane 3:Model Group

Lane 4:DB Group

Lane 5:Z-VAD-FMK Group

IL-1β Blot 2

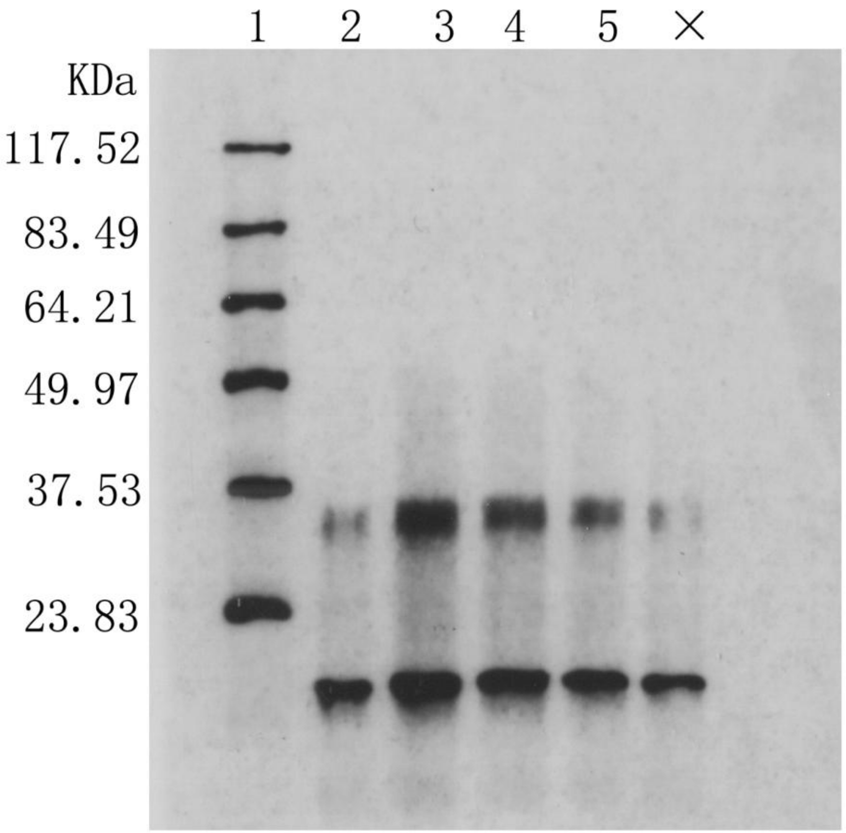

Lane 1:Marker

Lane 2:Control Group

Lane 3:Model Group

Lane 4:DB Group

Lane 5:Z-VAD-FMK Group

IL-1β Blot 3

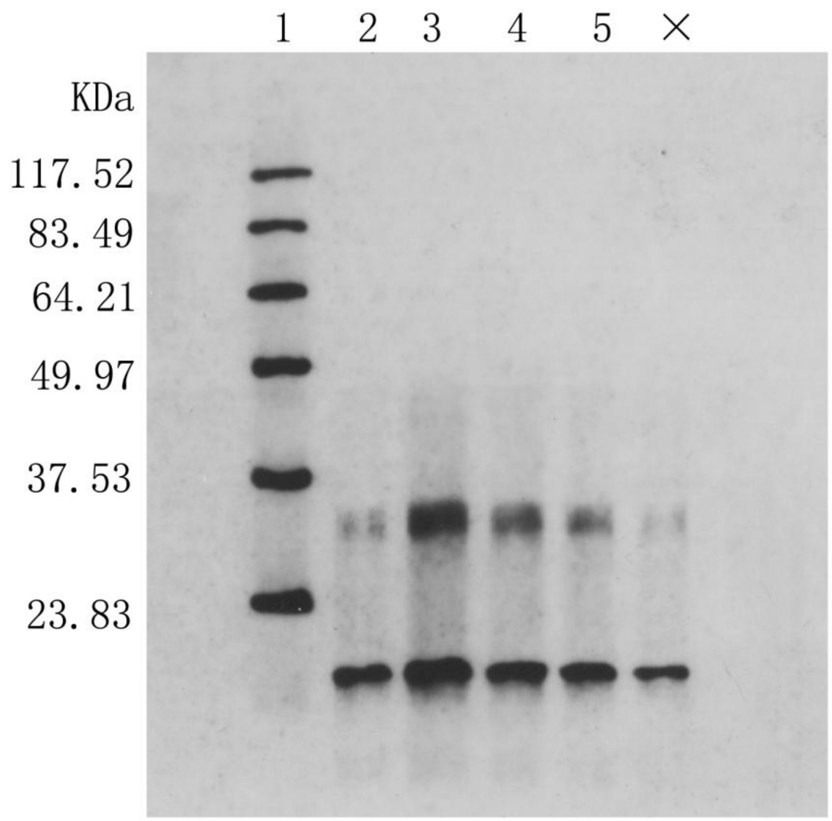

Lane 1:Marker

Lane 2:Control Group

Lane 3:Model Group

Lane 4:DB Group

Lane 5:Z-VAD-FMK Group

IL-18 Blot 1

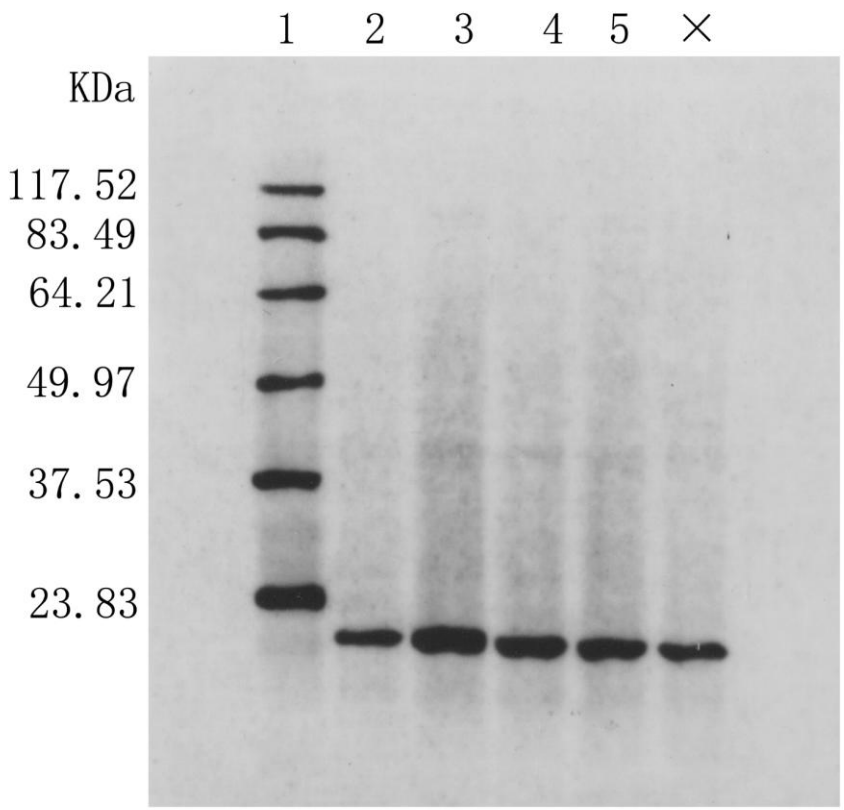

Lane 1:Marker

Lane 2:Control Group

Lane 3:Model Group

Lane 4:DB Group

Lane 5:Z-VAD-FMK Group

IL-18 Blot 2

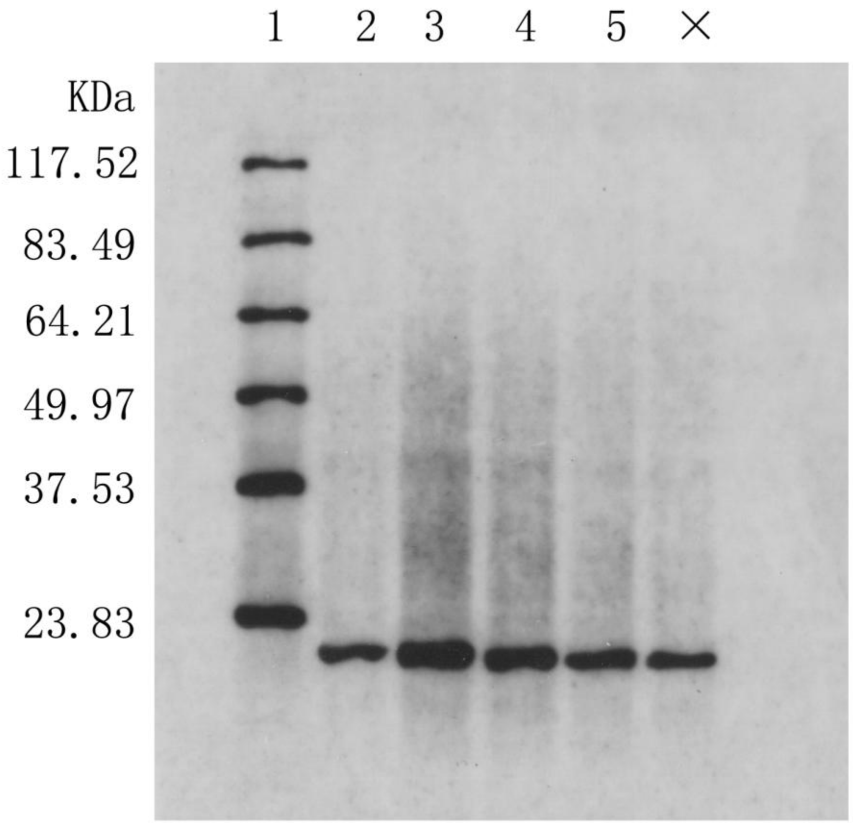

Lane 1:Marker

Lane 2:Control Group

Lane 3:Model Group

Lane 4:DB Group

Lane 5:Z-VAD-FMK Group

IL-18 Blot 3

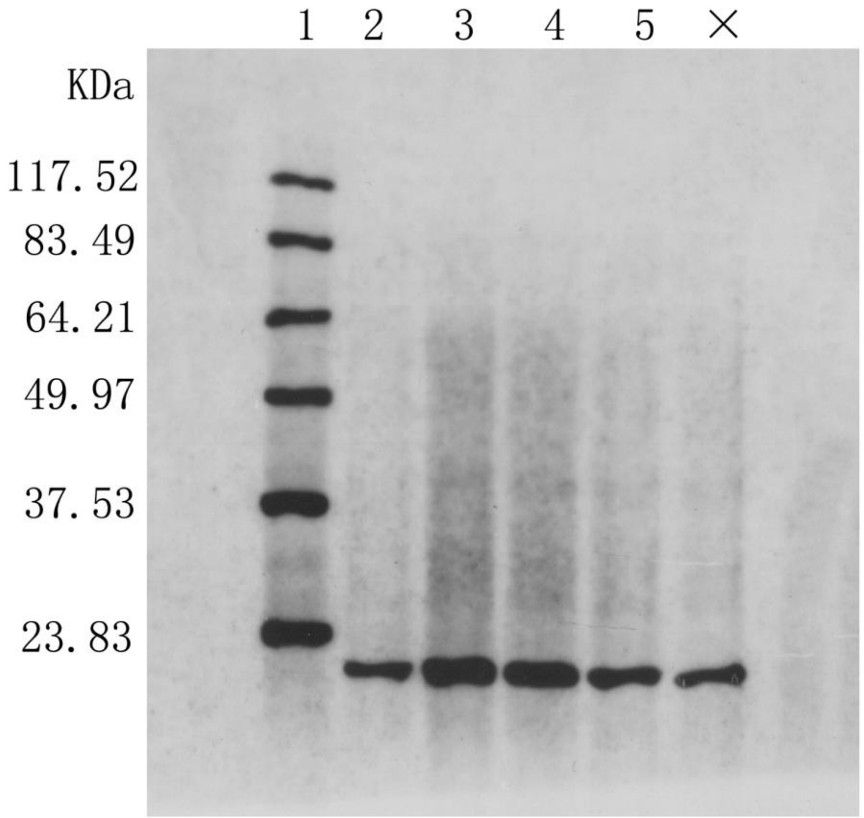

Lane 1:Marker

Lane 2:Control Group

Lane 3:Model Group

Lane 4:DB Group

Lane 5:Z-VAD-FMK Group

NLRP3 Blot 1

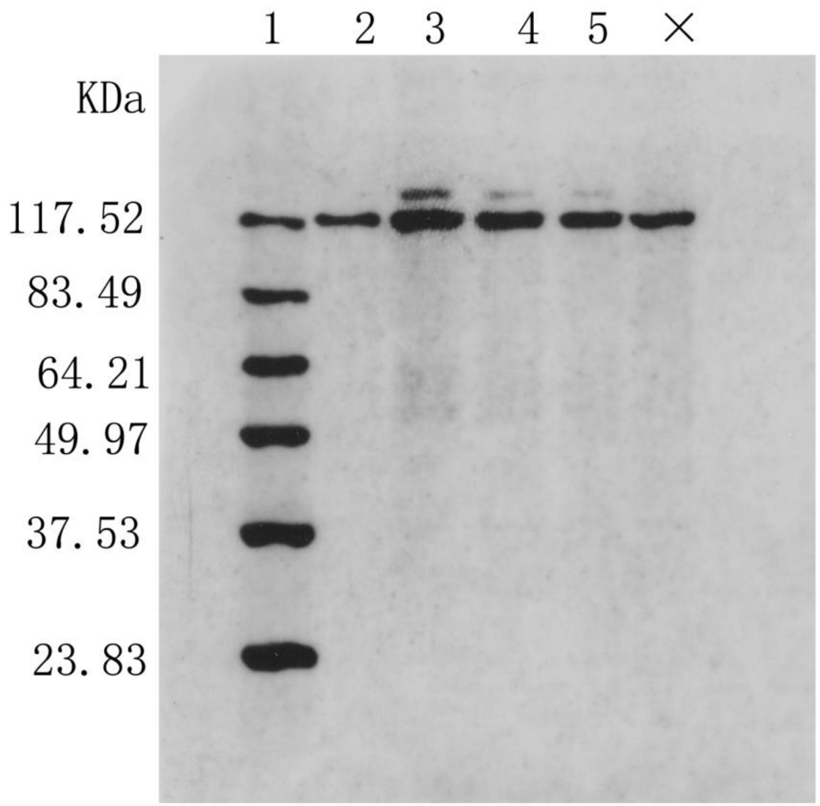

Lane 1:Marker

Lane 2:Control Group

Lane 3:Model Group

Lane 4:DB Group

Lane 5:Z-VAD-FMK Group

NLRP3 Blot 2

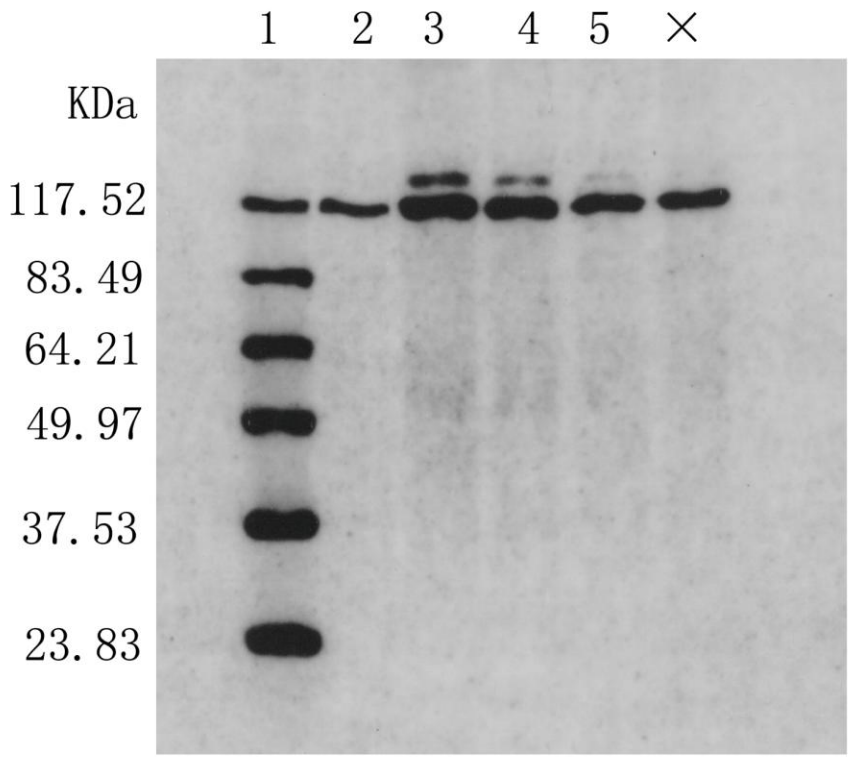

Lane 1:Marker

Lane 2:Control Group

Lane 3:Model Group

Lane 4:DB Group

Lane 5:Z-VAD-FMK Group

NLRP3 Blot 3

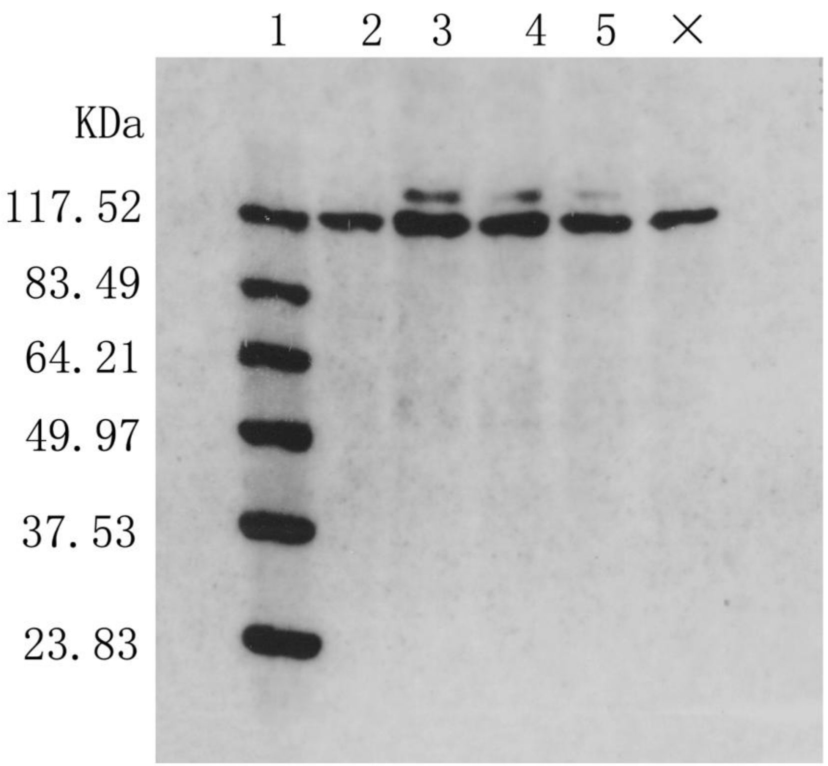

Lane 1:Marker

Lane 2:Control Group

Lane 3:Model Group

Lane 4:DB Group

Lane 5:Z-VAD-FMK Group

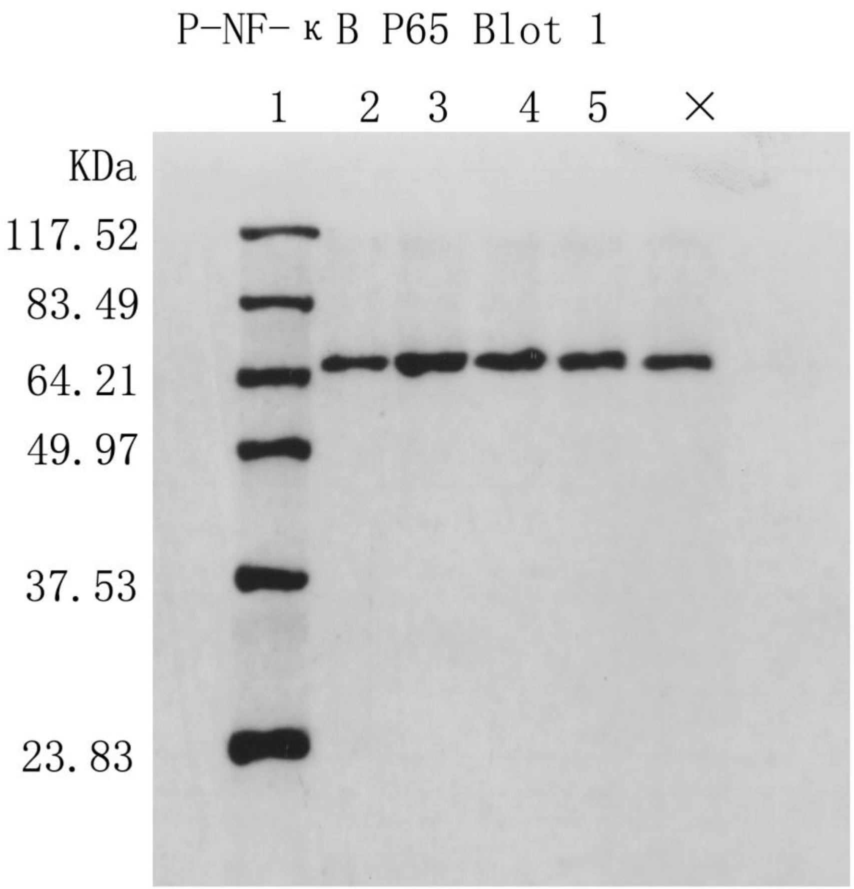

Lane 1:Marker

Lane 2:Control Group

Lane 3:Model Group

Lane 4:DB Group

Lane 5:Z-VAD-FMK Group

P-NF-κB P65 Blot 2

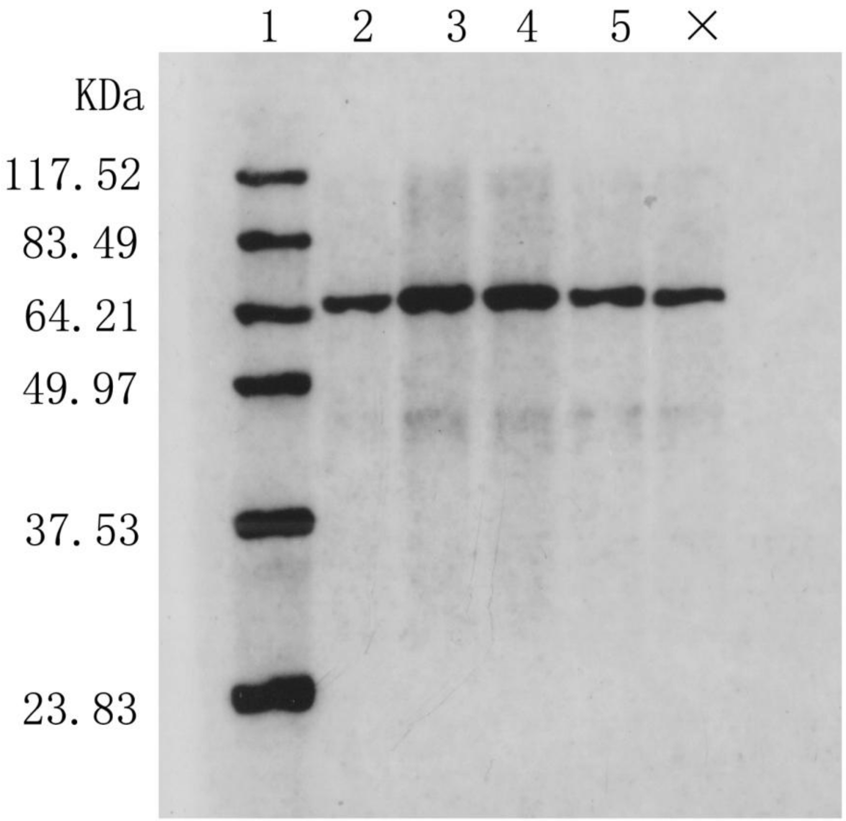

Lane 1:Marker

Lane 2:Control Group

Lane 3:Model Group

Lane 4:DB Group

Lane 5:Z-VAD-FMK Group

NF-κB P65 Blot 3

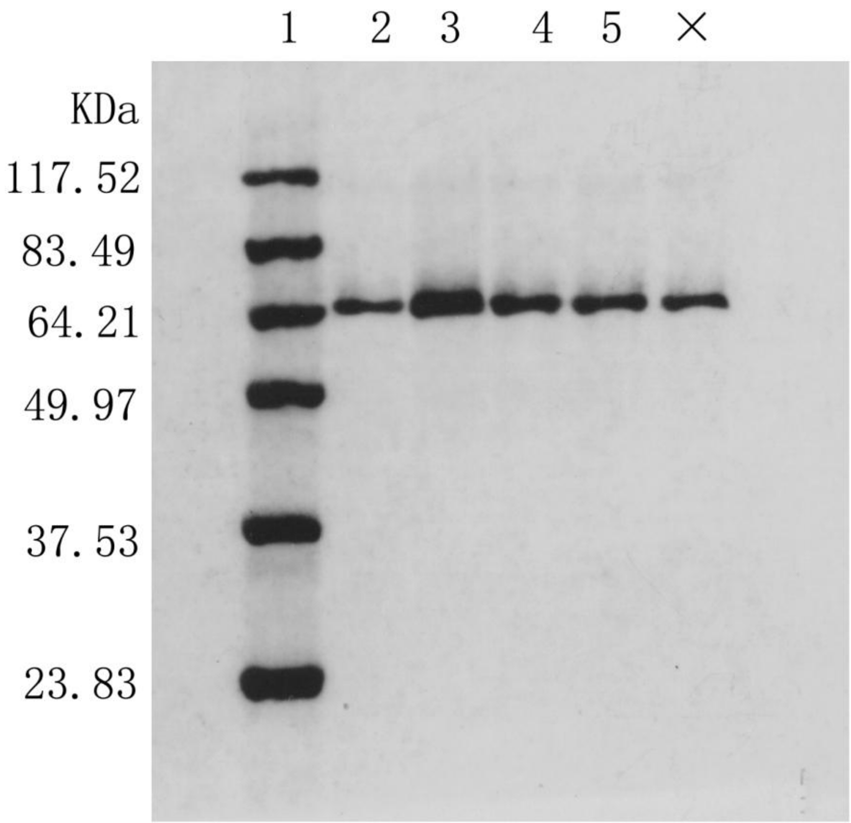

Lane 1:Marker

Lane 2:Control Group

Lane 3:Model Group

Lane 4:DB Group

Lane 5:Z-VAD-FMK Group

TLR4 Blot 1

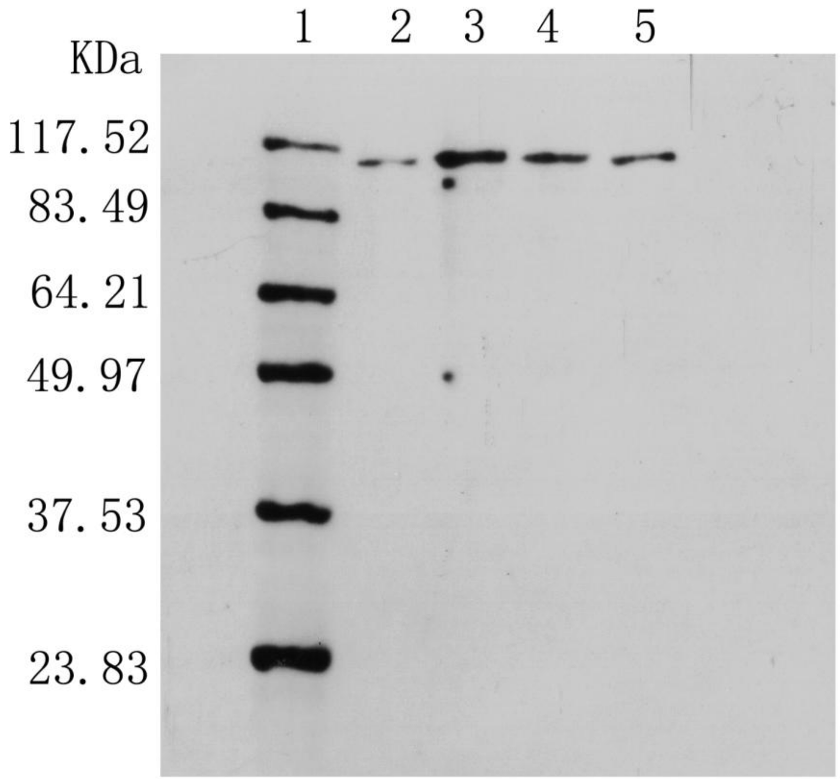

Lane 1:Marker

Lane 2:Control Group

Lane 3:Model Group

Lane 4:DB Group

Lane 5:Z-VAD-FMK Group

TLR4 Blot 2

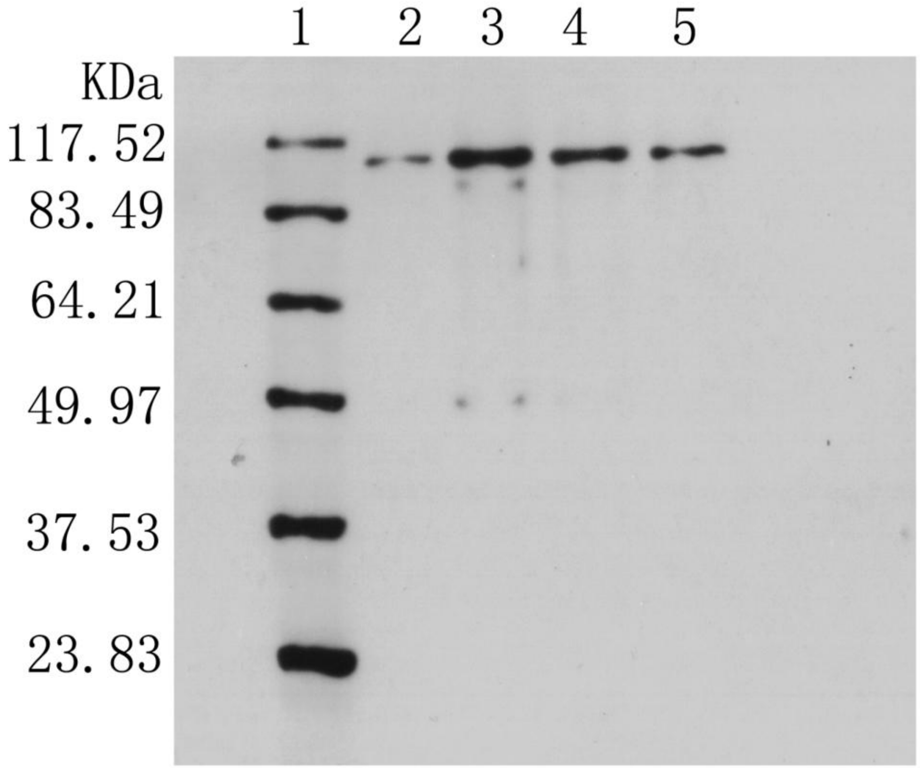

Lane 1:Marker

Lane 2:Control Group

Lane 3:Model Group

Lane 4:DB Group

Lane 5:Z-VAD-FMK Group

TLR4 Blot 3

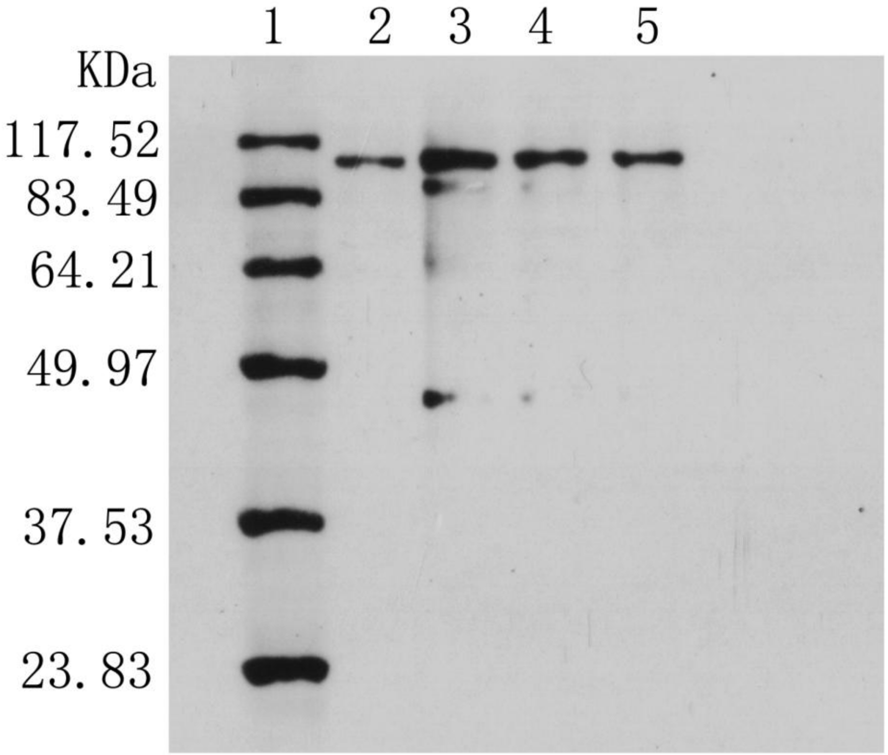

Lane 1:Marker

Lane 2:Control Group

Lane 3:Model Group

Lane 4:DB Group

Lane 5:Z-VAD-FMK Group

TNF-α Blot 1

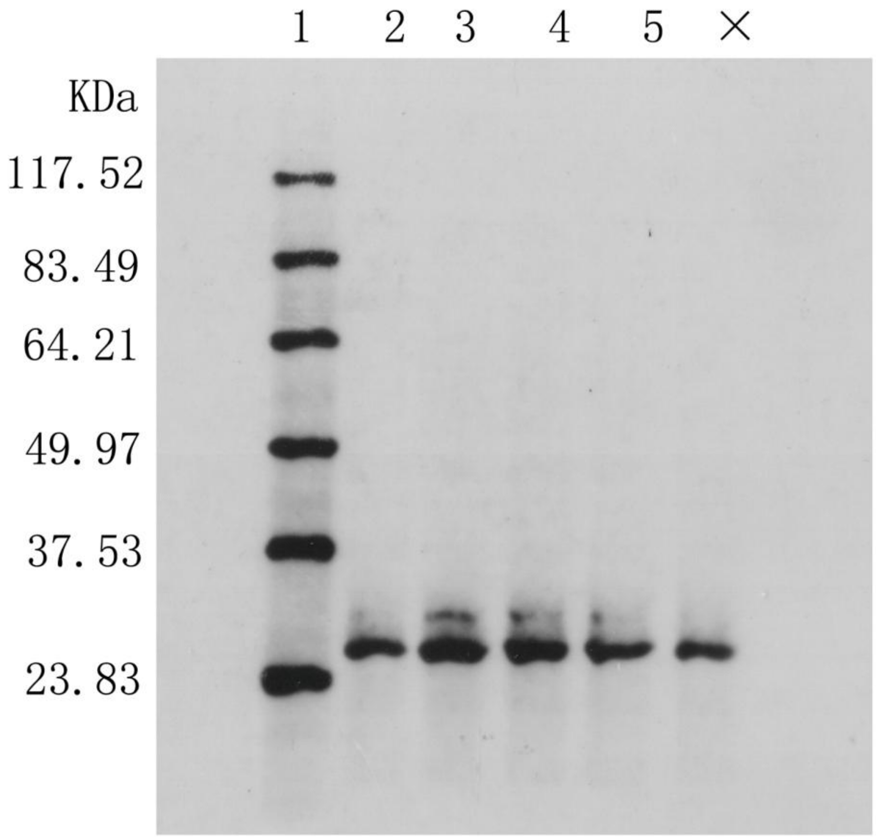

Lane 1:Marker

Lane 2:Control Group

Lane 3:Model Group

Lane 4:DB Group

Lane 5:Z-VAD-FMK Group

TNF-α Blot 2

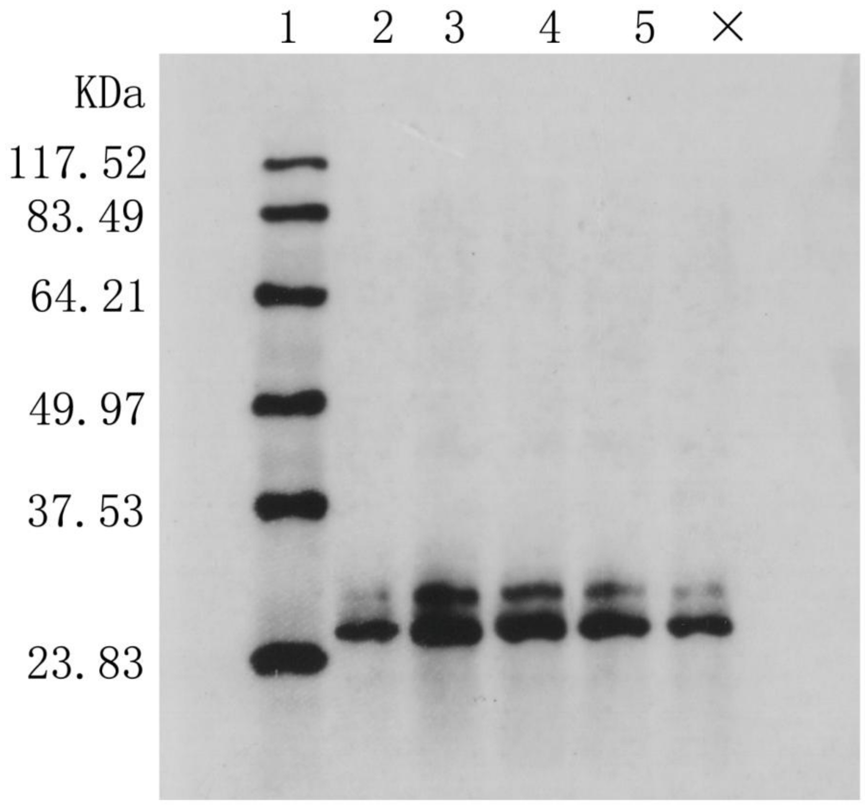

Lane 1:Marker

Lane 2:Control Group

Lane 3:Model Group

Lane 4:DB Group

Lane 5:Z-VAD-FMK Group

TNF-α Blot 3

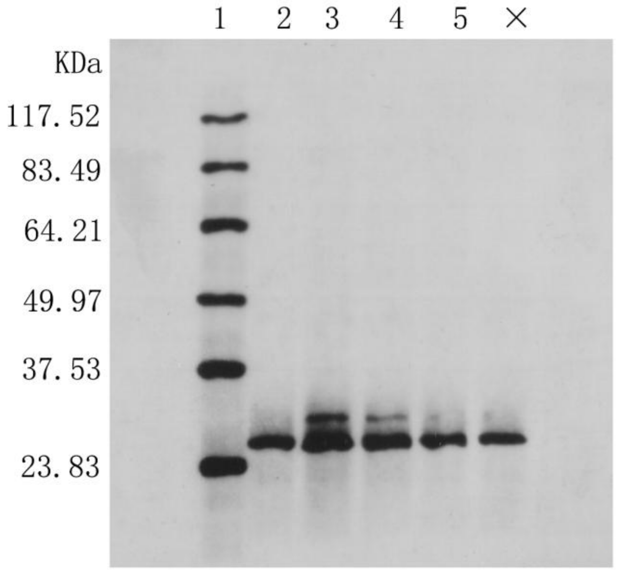

Lane 1:Marker

Lane 2:Control Group

Lane 3:Model Group

Lane 4:DB Group

Lane 5:Z-VAD-FMK Group

β-actin Blot 1 of TLR4

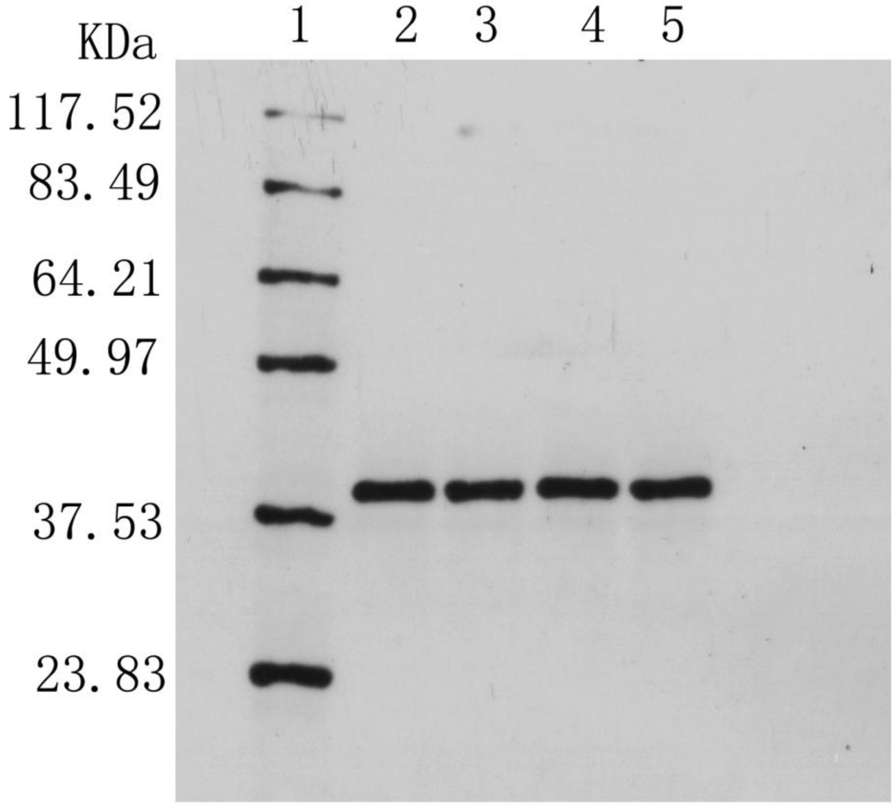

Lane 1:Marker

Lane 2:Control Group

Lane 3:Model Group

Lane 4:DB Group

Lane 5:Z-VAD-FMK Group

β-actin Blot 1

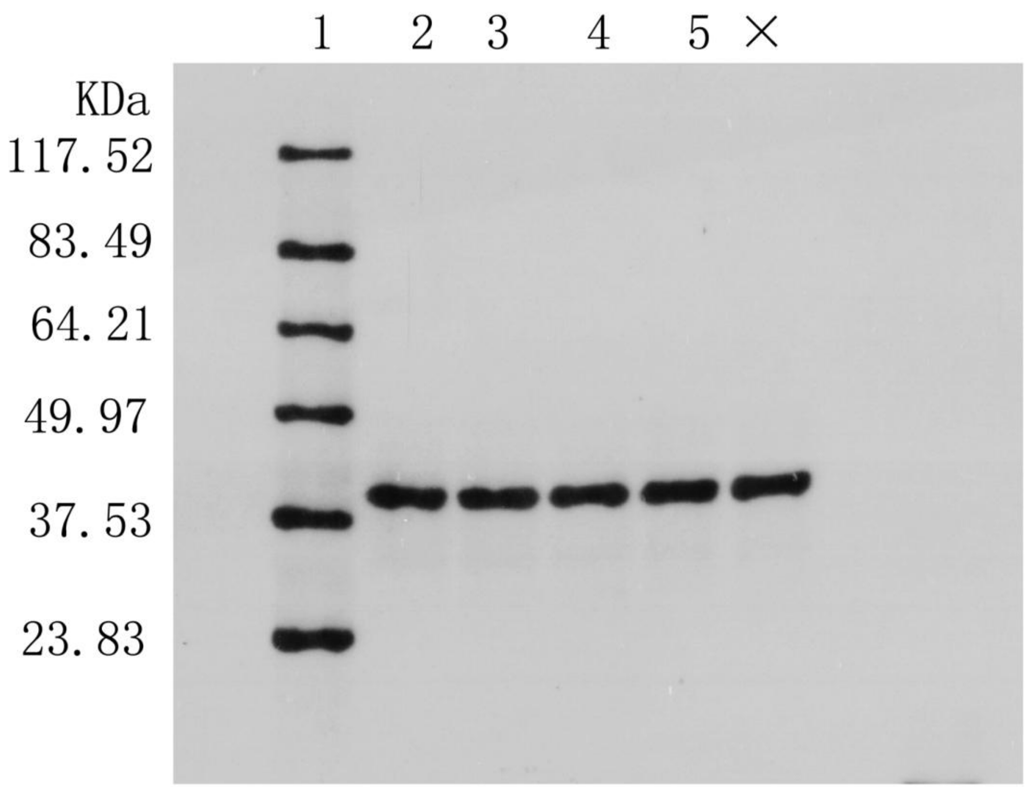

Lane 1:Marker

Lane 2:Control Group

Lane 3:Model Group

Lane 4:DB Group

Lane 5:Z-VAD-FMK Group

β-actin Blot 2 of TLR4

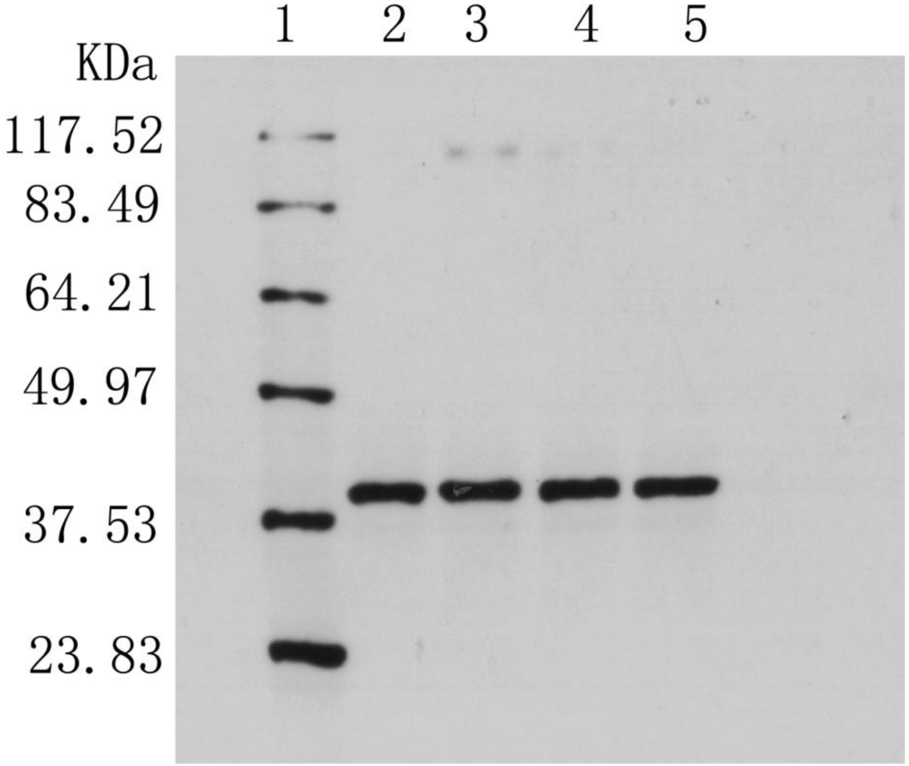

Lane 1:Marker

Lane 2:Control Group

Lane 3:Model Group

Lane 4:DB Group

Lane 5:Z-VAD-FMK Group

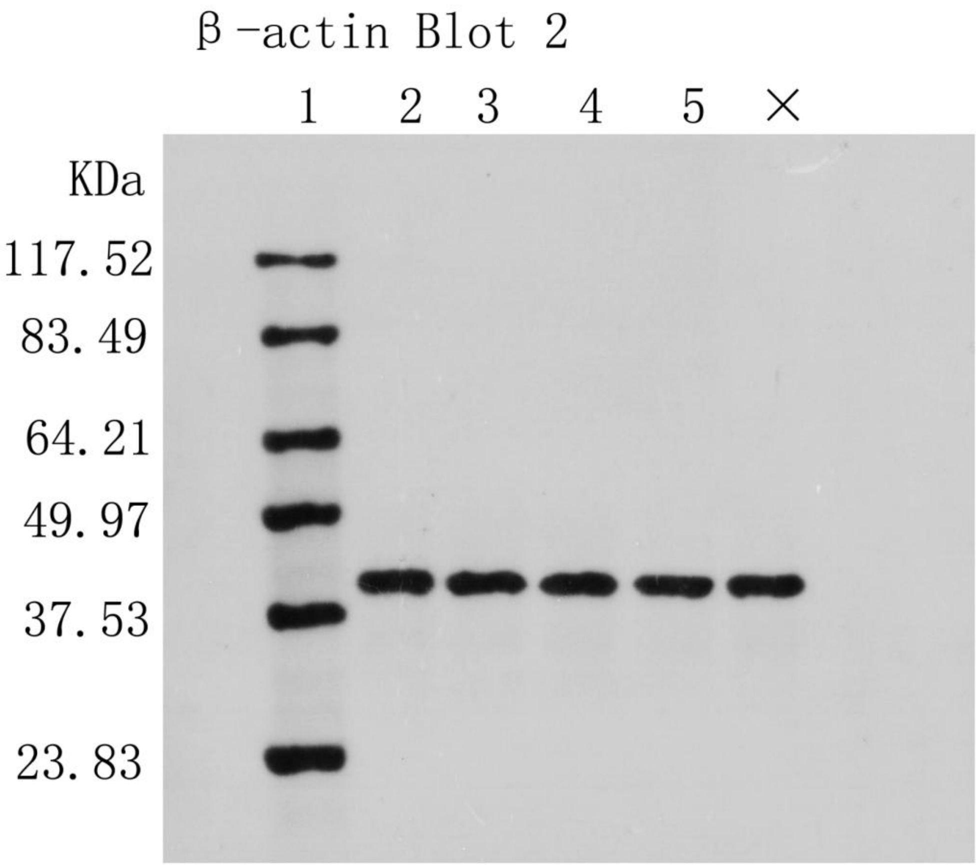

Lane 1:Marker

Lane 2:Control Group

Lane 3:Model Group

Lane 4:DB Group

Lane 5:Z-VAD-FMK Group

β-actin Blot 3 of TLR4

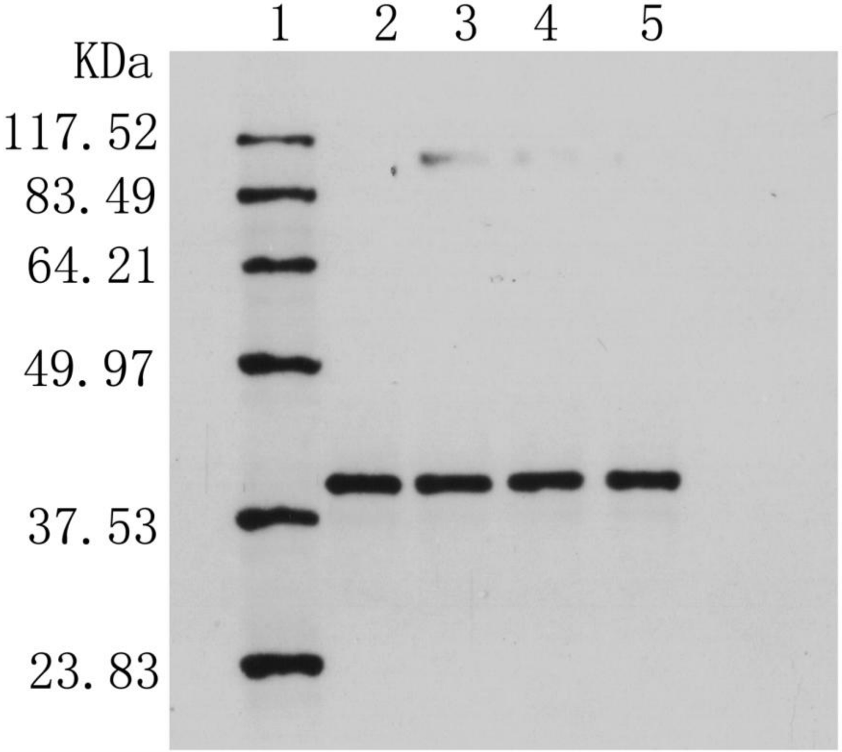

Lane 1:Marker

Lane 2:Control Group

Lane 3:Model Group

Lane 4:DB Group

Lane 5:Z-VAD-FMK Group

β-actin Blot 3

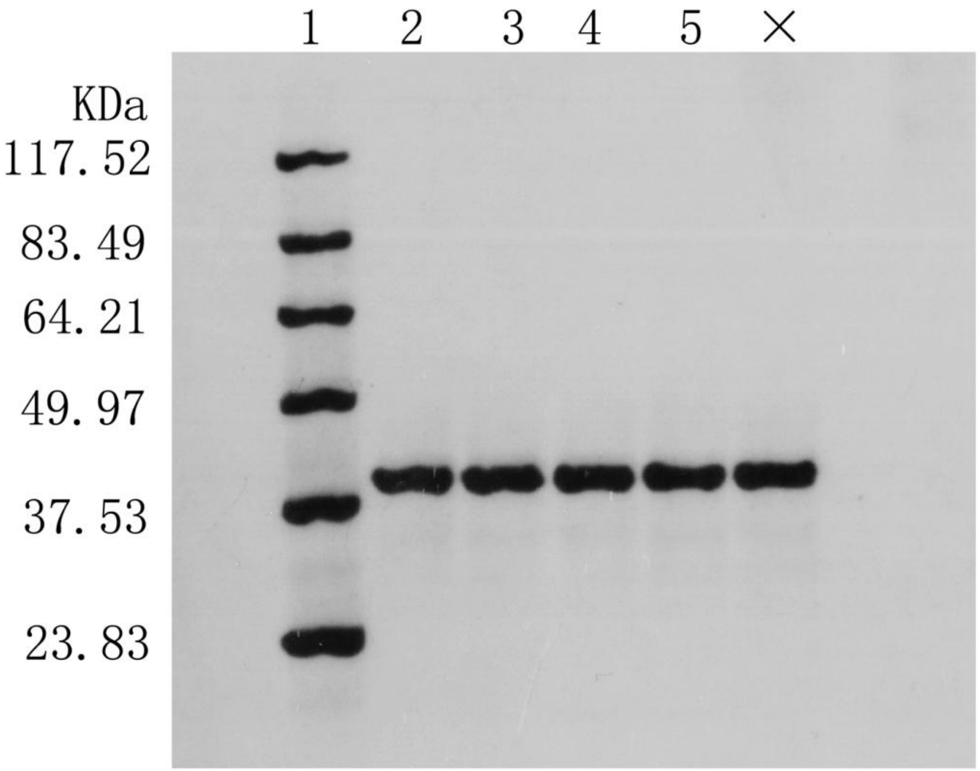

Lane 1:Marker

Lane 2:Control Group

Lane 3:Model Group

Lane 4:DB Group

Lane 5:Z-VAD-FMK Group
